# Supplementary material for: Modifiable and non-modifiable risk factors for COVID-19, and comparison to risk factors for influenza and pneumonia: results from a UK Biobank prospective cohort study
Source: BMJ Open. 2020 Nov 19;10(11):e040402. doi: 10.1136/bmjopen-2020-040402 (PMC7678347; doi:10.1136/bmjopen-2020-040402)

Modifiable and non-modifiable risk factors for COVID-19, and comparison to risk factors for influenza and pneumonia: results from a UK Biobank prospective cohort study

Supplementary Material

Frederick K Ho*, Carlos A Celis-Morales*, Stuart R Gray, S Vittal Katikireddi, Claire L Niedzwiedz, Claire Hastie, Lyn D Ferguson, Colin Berry, Daniel F Mackay, Jason MR Gill, Jill P Pell**, Naveed Sattar MD**, Paul Welsh**

*Joint first authors

**Joint senior

Ho FK, *et al. BMJ Open* 2020; 10:e040402. doi: 10.1136/bmjopen-2020-040402

SUPPLEMENTARY MATERIAL

Supplementary Table 1. Univariable association of baseline risk factors pneumonia and influenza occurring any time after baseline

	COVID-19 in all settings				Pneumonia since baseline				Influenza since baseline			
	No n=235410	Yes n=518	RR*	P	No n=94015	Yes n=2799	RR*	P	No n=95452	Yes n=1362	RR*	P
Age (years)	66.53 (8.09)	67.04 (8.93)	1.07	0.15	66.51 (8.06)	67.24 (8.18)	1.09	< 0.0001	66.54 (8.06)	65.63 (8.17)	0.90	< 0.0001
Age categories (years)												
<60	58639 (24.91)	146 (28.19)	1	REF	23107 (24.58)	631 (22.54)	1	REF	23349 (24.46)	389 (28.56)	1	REF
60-64	37569 (15.96)	62 (11.97)	0.66	0.007	14884 (15.83)	370 (13.22)	0.91	0.16	15027 (15.74)	227 (16.67)	0.91	0.25
65-69	42802 (18.18)	68 (13.13)	0.64	0.002	17128 (18.22)	523 (18.69)	1.11	0.06	17392 (18.22)	259 (19.02)	0.90	0.17
70-74	55711 (23.67)	108 (20.85)	0.78	0.049	22584 (24.02)	712 (25.44)	1.15	0.010	23000 (24.10)	296 (21.73)	0.78	0.0009
≥75	40689 (17.28)	134 (25.87)	1.32	0.02	16312 (17.35)	563 (20.11)	1.26	< 0.0001	16684 (17.48)	191 (14.02)	0.69	< 0.0001
Male sex, n (%)	111029 (47.16)	285 (55.02)	1.37	0.0004	44786 (47.64)	834 (29.80)	0.48	< 0.0001	45041 (47.19)	579 (42.51)	0.83	0.0006
Ethnicity, n (%)												
White	224116 (95.20)	455 (87.84)	1	REF	90008 (95.74)	2670 (95.39)	1	REF	91423 (95.78)	1255 (92.14)	1	REF
Black	3045 (1.29)	26 (5.02)	4.18	< 0.0001	879 (0.93)	32 (1.14)	1.22	0.26	899 (0.94)	12 (0.88)	0.97	0.92
South Asian	3502 (1.49)	18 (3.47)	2.52	0.0001	1546 (1.64)	62 (2.22)	1.34	0.02	1560 (1.63)	48 (3.52)	2.20	< 0.0001
Others	4747 (2.02)	19 (3.67)	1.97	0.004	1582 (1.68)	35 (1.25)	0.75	0.09	1570 (1.64)	47 (3.45)	2.15	< 0.0001
Deprivation index (score)	-1.44 (2.98)	-0.50 (3.39)	1.32	< 0.0001	-1.49 (2.89)	-1.19 (3.07)	1.10	< 0.0001	-1.49 (2.89)	-0.98 (3.16)	1.18	< 0.0001
Current/former smoker, n (%)	106773 (45.36)	280 (54.05)	1.42	< 0.0001	42567 (45.28)	1369 (48.91)	1.15	0.0001	43261 (45.32)	675 (49.56)	1.18	0.002
Alcohol drinking status, n (%)												
Never	8885 (3.77)	36 (6.95)	1	REF	3573 (3.80)	154 (5.50)	1	REF	3668 (3.84)	59 (4.33)	1	REF
Former	7464 (3.17)	24 (4.63)	0.79	0.38	2963 (3.15)	140 (5.00)	1.09	0.44	3045 (3.19)	58 (4.26)	1.18	0.37
Current	219061 (93.06)	458 (88.42)	0.52	0.0001	87479 (93.05)	2505 (89.50)	0.67	< 0.0001	88739 (92.97)	1245 (91.41)	0.87	0.31
BMI (kg/m²)	27.31 (4.56)	29.15 (5.29)	1.40	< 0.0001	27.40 (4.55)	27.88 (5.01)	1.10	< 0.0001	27.41 (4.56)	27.74 (4.81)	1.07	0.009
BMI categories, n (%)												
Underweight	787 (0.33)	1 (0.19)	0.87	0.89	295 (0.31)	11 (0.39)	1.29	0.40	306 (0.32)	0 (0.00)	0.00	0.92
Normal	78044 (33.15)	114 (22.01)	1	REF	30371 (32.30)	873 (31.19)	1	REF	30821 (32.29)	423 (31.06)	1	REF
Overweight	101703 (43.20)	220 (42.47)	1.48	0.0007	40839 (43.44)	1108 (39.59)	0.95	0.21	41388 (43.36)	559 (41.04)	0.98	0.80
Obese	54876 (23.31)	183 (35.33)	2.28	< 0.0001	22510 (23.94)	807 (28.83)	1.24	< 0.0001	22937 (24.03)	380 (27.90)	1.20	0.008
Body fat-free mass (Kg)	53.76 (11.47)	56.39 (11.55)	1.25	< 0.0001	53.84 (11.50)	50.54 (10.66)	0.74	< 0.0001	53.75 (11.50)	52.98 (11.20)	0.93	0.01
Body fat mass (Kg)	24.49 (9.19)	27.48 (10.59)	1.32	< 0.0001	24.64 (9.19)	26.67 (9.89)	1.21	< 0.0001	24.69 (9.21)	25.47 (9.56)	1.08	0.002
Body fat proportion (percent)	31.01 (8.40)	32.27 (8.56)	1.16	0.0007	31.11 (8.40)	34.05 (8.28)	1.40	< 0.0001	31.18 (8.41)	32.09 (8.32)	1.11	< 0.0001
Systolic blood pressure (mmHg)	137.24 (18.07)	138.40 (18.89)	1.07	0.14	137.60 (18.11)	135.90 (17.67)	0.91	< 0.0001	137.59 (18.10)	135.00 (17.69)	0.86	< 0.0001
Diastolic blood pressure (mmHg)	82.07 (9.90)	83.13 (10.49)	1.11	0.02	82.22 (9.90)	81.22 (9.60)	0.91	< 0.0001	82.20 (9.89)	81.31 (10.16)	0.91	0.0010
FEV1 (litres)	2.86 (0.77)	2.75 (0.78)	0.86	0.0007	2.87 (0.78)	2.57 (0.73)	0.67	< 0.0001	2.86 (0.78)	2.73 (0.78)	0.84	< 0.0001
FVC (litres)	3.79 (0.98)	3.68 (0.97)	0.89	0.01	3.80 (0.99)	3.44 (0.91)	0.68	< 0.0001	3.79 (0.99)	3.63 (0.96)	0.85	< 0.0001
FEV1/FVC	0.76 (0.06)	0.75 (0.07)	0.88	0.002	0.76 (0.06)	0.75 (0.07)	0.89	< 0.0001	0.76 (0.06)	0.75 (0.07)	0.95	0.045
Walking pace, n (%)												
Slow	14872 (6.32)	74 (14.29)	2.15	< 0.0001	5895 (6.27)	324 (11.58)	1.74	< 0.0001	6098 (6.39)	121 (8.88)	1.35	0.002
Average	123516 (52.47)	285 (55.02)	1	REF	49425 (52.57)	1529 (54.63)	1	REF	50222 (52.61)	732 (53.74)	1	REF

Ho FK, *et al. BMJ Open* 2020; 10:e040402. doi: 10.1136/bmjopen-2020-040402

Brisk	97022 (41.21)	159 (30.69)	0.71	0.0006	38695 (41.16)	946 (33.80)	0.80	< 0.0001	39132 (41.00)	509 (37.37)	0.89	0.050
Grip strength (Kg)	31.31 (10.93)	31.44 (10.39)	1.01	0.79	31.31 (10.97)	27.46 (9.99)	0.69	< 0.0001	31.22 (10.96)	29.99 (10.96)	0.89	< 0.0001
Prevalent disease at baseline, n (%)												
Longstanding illness	68156 (28.95)	208 (40.15)	1.64	< 0.0001	27777 (29.55)	1211 (43.27)	1.78	< 0.0001	28500 (29.86)	488 (35.83)	1.31	< 0.0001
Diabetes	10959 (4.66)	48 (9.27)	2.09	< 0.0001	4386 (4.67)	209 (7.47)	1.62	< 0.0001	4510 (4.72)	85 (6.24)	1.34	0.009
CVD	11229 (4.77)	44 (8.49)	1.85	< 0.0001	4689 (4.99)	199 (7.11)	1.44	< 0.0001	4805 (5.03)	83 (6.09)	1.22	0.08
Cancer	15841 (6.75)	40 (7.77)	1.16	0.36	6339 (6.76)	245 (8.80)	1.32	< 0.0001	6480 (6.81)	104 (7.66)	1.13	0.22
Depression	45765 (19.44)	84 (16.22)	0.80	0.06	18180 (19.34)	505 (18.04)	0.92	0.09	18417 (19.29)	268 (19.68)	1.02	0.72
CKD stages 3-5	291 (0.12)	4 (0.77)	6.22	0.0003	122 (0.13)	6 (0.21)	1.62	0.23	125 (0.13)	3 (0.22)	1.67	0.37
SLE	274 (0.12)	0 (0.00)	0.00	0.96	115 (0.12)	10 (0.36)	2.77	0.001	123 (0.13)	2 (0.15)	1.14	0.85
Asthma	27705 (11.77)	71 (13.71)	1.19	0.17	10897 (11.59)	570 (20.36)	1.90	< 0.0001	11250 (11.79)	217 (15.93)	1.41	< 0.0001
Sleep apnoea	718 (0.30)	4 (0.77)	2.54	0.06	301 (0.32)	16 (0.57)	1.75	0.02	311 (0.33)	6 (0.44)	1.35	0.46
COPD	535 (0.23)	4 (0.77)	3.40	0.01	220 (0.23)	21 (0.75)	3.03	< 0.0001	231 (0.24)	10 (0.73)	2.96	0.0006
Bronchitis	2514 (1.07)	9 (1.74)	1.64	0.14	1011 (1.08)	80 (2.86)	2.58	< 0.0001	1061 (1.11)	30 (2.20)	1.98	0.0002
Pneumonia	2935 (1.25)	11 (2.12)	1.72	0.08	1115 (1.19)	41 (1.46)	1.23	0.18	1140 (1.19)	16 (1.17)	0.98	0.95
Other respiratory disease	1242 (0.53)	4 (0.77)	1.47	0.45	489 (0.52)	23 (0.82)	1.56	0.03	508 (0.53)	4 (0.29)	0.55	0.23
Medication at baseline, n (%)												
Statin	36295 (15.42)	117 (22.59)	1.60	< 0.0001	14836 (15.78)	561 (20.04)	1.33	< 0.0001	15161 (15.88)	236 (17.33)	1.11	0.15
BP medication	37560 (15.96)	135 (26.06)	1.85	< 0.0001	15249 (16.22)	504 (18.01)	1.13	0.01	15504 (16.24)	249 (18.28)	1.15	0.04
Steroid	876 (0.37)	1 (0.19)	0.52	0.51	334 (0.36)	21 (0.75)	2.05	0.0009	352 (0.37)	3 (0.22)	0.60	0.37
Biomarker at baseline												
Total cholesterol (mmol/L)	5.62 (4.92-6.34)	5.45 (4.71-6.20)	0.84	< 0.0001	5.63 (4.91-6.35)	5.52 (4.80-6.26)	0.92	< 0.0001	5.63 (4.91-6.34)	5.51 (4.83-6.26)	0.92	0.0010
HDL cholesterol (mmol/L)	1.40 (1.17-1.67)	1.29 (1.09-1.53)	0.71	< 0.0001	1.39 (1.17-1.67)	1.42 (1.18-1.70)	1.05	0.004	1.40 (1.17-1.67)	1.37 (1.15-1.65)	0.93	0.01
Cystatin C (mg/L)	0.88 (0.80-0.97)	0.93 (0.83-1.02)	1.35	< 0.0001	0.88 (0.80-0.97)	0.88 (0.79-0.99)	1.05	0.01	0.88 (0.80-0.97)	0.89 (0.80-0.99)	1.08	0.004
HbA1c (mmol/mol)	35.10 (32.60-37.60)	35.90 (33.40-39.38)	1.26	< 0.0001	35.10 (32.60-37.60)	35.60 (33.10-38.50)	1.16	< 0.0001	35.10 (32.60-37.60)	35.30 (32.80-38.20)	1.08	0.0005
CRP (mg/L)	1.25 (0.63-2.56)	1.67 (0.87-3.23)	1.26	< 0.0001	1.26 (0.63-2.57)	1.54 (0.78-3.19)	1.16	< 0.0001	1.26 (0.64-2.58)	1.46 (0.71-3.04)	1.08	0.0005
Rheumatoid factor (IU/ml)	3.16 (3.16-3.16)	3.16 (3.16-3.16)	1.15	< 0.0001	3.16 (3.16-3.16)	3.16 (3.16-3.16)	1.13	< 0.0001	3.16 (3.16-3.16)	3.16 (3.16-3.16)	1.11	< 0.0001
Red cell distribution width (%)	13.30 (12.90-13.80)	13.34 (12.97-13.94)	1.03	0.50	13.30 (12.89-13.80)	13.39 (12.93-13.91)	1.04	0.03	13.30 (12.89-13.80)	13.30 (12.90-13.80)	1.04	0.07
White cell count (10^9 cells/Litre)	6.60 (5.61-7.80)	6.82 (5.82-8.20)	1.14	0.0005	6.62 (5.63-7.80)	6.89 (5.83-8.18)	1.11	< 0.0001	6.63 (5.64-7.80)	6.86 (5.75-8.07)	1.02	0.39
Neutrophil count (10^9 cells/Litre)	4.00 (3.25-4.90)	4.11 (3.30-5.11)	1.15	0.0007	4.00 (3.26-4.91)	4.20 (3.42-5.10)	1.16	< 0.0001	4.01 (3.26-4.92)	4.19 (3.32-5.07)	1.12	< 0.0001
Lymphocyte count (10^9 cells/Litre)	1.88 (1.52-2.29)	1.90 (1.55-2.40)	1.11	0.01	1.88 (1.52-2.29)	1.92 (1.55-2.39)	1.14	< 0.0001	1.88 (1.52-2.29)	1.90 (1.53-2.36)	1.10	0.0003
Monocyte count (10^9 cells/Litre)	0.45 (0.36-0.56)	0.48 (0.38-0.58)	1.11	0.01	0.45 (0.37-0.57)	0.45 (0.37-0.57)	1.10	< 0.0001	0.45 (0.37-0.57)	0.46 (0.37-0.58)	1.07	0.01

Numbers represent number (%) for categorical variables, mean (SD) for gaussian continuous variables, and median (25-75th percentiles) for skewed variables

*Univariable relative risk ratio per 1 standard deviation for continuous variables and using comparator group for categorical variables.

Ho FK, *et al. BMJ Open* 2020; 10:e040402. doi: 10.1136/bmjopen-2020-040402

Supplementary table 2. Sensitivity analysis for pneumonia occurring after 2015.

	**Pneumonia (after 2015)**				**Model 1**		**Model 2**	
	**No n=117181**	**Yes n=767**	**RR**	**P**	**RR (95% CI)**	**P**	**RR (95% CI)**	**P**
Mean (SD) Age	66.52 (8.06)	68.44 (7.87)	1.28	< 0.0001	1.18 (1.12, 1.24)	< 0.0001	1.10 (1.04, 1.17)	0.001
Age categories								
<50	23642 (24.57)	96 (15.79)	1	REF	-	-	-	-
50-54	15174 (15.77)	80 (13.16)	1.30	0.09	-	-	-	-
55-59	17523 (18.21)	128 (21.05)	1.79	< 0.0001	-	-	-	-
60-64	23142 (24.05)	154 (25.33)	1.63	0.0002	-	-	-	-
≥65	16725 (17.38)	150 (24.67)	2.20	< 0.0001	-	-	-	-
Male	45416 (47.21)	204 (33.55)	0.57	< 0.0001	0.56 (0.47, 0.66)	< 0.0001	0.72 (0.58, 0.89)	0.003
Ethnicity								
White	92091 (95.72)	587 (96.55)	1	REF	1 (Reference)	-	1 (Reference)	-
Black	906 (0.94)	5 (0.82)	0.87	0.75	0.88 (0.36, 2.13)	0.78	0.72 (0.29, 1.75)	0.47
South Asian	1600 (1.66)	8 (1.32)	0.79	0.50	0.85 (0.42, 1.71)	0.65	0.66 (0.32, 1.34)	0.25
Others	1609 (1.67)	8 (1.32)	0.78	0.49	0.82 (0.41, 1.66)	0.59	0.72 (0.36, 1.45)	0.36
Mean (SD) deprivation index	-1.49 (2.90)	-1.22 (3.01)	1.09	0.03	1.12 (1.04, 1.22)	0.004	1.07 (0.98, 1.16)	0.11
Current/former smoker	43642 (45.36)	294 (48.36)	1.13	0.14	0.95 (0.72, 1.25)	0.71	1.07 (0.91, 1.26)	0.43
Alcohol drinking status								
Never	3696 (3.84)	31 (5.10)	1	REF	1 (Reference)	-	-	-
Former	3077 (3.20)	26 (4.28)	1.01	0.98	1.28 (0.58, 2.79)	0.54	-	-
Current	89433 (92.96)	551 (90.62)	0.74	0.10	0.66 (0.37, 1.18)	0.16	-	-
Mean (SD) BMI	27.41 (4.56)	28.16 (5.11)	1.16	< 0.0001	1.25 (1.11, 1.41)	0.0002	1.09 (1.01, 1.18)	0.03
BMI categories								
Underweight	304 (0.32)	2 (0.33)	1.10	0.90	1.43 (0.20, 10.4)	0.73	-	-
Normal	31058 (32.28)	186 (30.59)	1	REF	1 (Reference)	-	-	-
Overweight	41705 (43.35)	242 (39.80)	0.97	0.75	1.01 (0.72, 1.42)	0.94	-	-
Obese	23139 (24.05)	178 (29.28)	1.28	0.02	1.44 (1.01, 2.05)	0.04	-	-
Mean (SD) Body fat-free mass	53.76 (11.49)	51.26 (11.04)	0.80	< 0.0001	1.42 (1.10, 1.84)	0.008	-	-
Mean (SD) Body fat mass	24.68 (9.21)	26.95 (10.05)	1.24	< 0.0001	1.27 (1.12, 1.44)	0.0002	-	-

Ho FK, *et al. BMJ Open* 2020; 10:e040402. doi: 10.1136/bmjopen-2020-040402

Mean (SD) Body fat percent	31.18 (8.41)	34.01 (8.33)	1.39	< 0.0001	1.34 (1.11, 1.62)	0.002	-	-
Mean (SD) Systolic blood pressure	137.56 (18.10)	137.18 (18.02)	0.98	0.61	0.94 (0.81, 1.08)	0.39	-	-
Mean (SD) Diastolic blood pressure	82.20 (9.90)	81.48 (9.76)	0.93	0.08	0.90 (0.79, 1.04)	0.16	-	-
Mean (SD) FEV1	2.86 (0.78)	2.56 (0.75)	0.66	< 0.0001	0.70 (0.58, 0.86)	0.0005	0.79 (0.70, 0.89)	< 0.0001
Mean (SD) FVC	3.79 (0.99)	3.43 (0.94)	0.67	< 0.0001	0.72 (0.59, 0.90)	0.003	-	-
Mean (SD) FEV1/FVC	0.76 (0.06)	0.74 (0.07)	0.86	< 0.0001	0.84 (0.74, 0.95)	0.007	-	-
Walking pace								
Slow	50630 (52.63)	324 (53.29)	1.90	< 0.0001	1.91 (1.26, 2.90)	0.002	1.53 (1.18, 2.00)	0.001
Average	6144 (6.39)	75 (12.34)	1	REF	1 (Reference)	-	1 (Reference)	-
Brisk	39432 (40.99)	209 (34.38)	0.83	0.03	0.78 (0.57, 1.06)	0.11	0.96 (0.80, 1.15)	0.65
Mean (SD) grip strength	31.22 (10.96)	27.84 (10.44)	0.72	< 0.0001	0.85 (0.68, 1.07)	0.16	0.96 (0.76, 1.22)	0.76
Prevalent disease at baseline								
Longstanding illness	28729 (29.86)	259 (42.60)	1.74	< 0.0001	1.65 (1.25, 2.18)	0.0004	1.40 (1.04, 1.88)	0.03
Diabetes	4549 (4.73)	46 (7.57)	1.64	0.001	2.06 (1.29, 3.30)	0.002	1.59 (0.98, 2.59)	0.06
CVD	4842 (5.03)	46 (7.57)	1.54	0.005	1.64 (1.00, 2.70)	0.052	1.35 (0.81, 2.24)	0.25
Cancer	6525 (6.80)	59 (9.80)	1.48	0.004	1.72 (1.14, 2.60)	0.009	1.72 (1.14, 2.60)	0.01
Depression	18587 (19.32)	98 (16.12)	0.80	0.047	0.81 (0.56, 1.18)	0.27	0.86 (0.59, 1.25)	0.42
Asthma	11342 (11.79)	125 (20.56)	1.93	< 0.0001	1.44 (0.99, 2.09)	0.054	1.25 (0.85, 1.82)	0.25
COPD	238 (0.25)	3 (0.49)	1.99	0.23	-	-	-	-
Bronchitis	1074 (1.12)	17 (2.80)	2.52	0.0002	2.23 (0.98, 5.06)	0.055	1.77 (0.77, 4.07)	0.18
Pneumonia	1144 (1.19)	12 (1.97)	1.67	0.08	1.53 (0.57, 4.14)	0.40	1.47 (0.54, 3.99)	0.45
Other respiratory disease	507 (0.53)	5 (0.82)	1.56	0.32	0.84 (0.12, 6.04)	0.86	0.76 (0.10, 5.52)	0.78
Medication at baseline								
Statin	15265 (15.87)	132 (21.71)	1.47	< 0.0001	1.08 (0.75, 1.55)	0.69	0.91 (0.62, 1.32)	0.62
BP medication	15636 (16.25)	117 (19.24)	1.23	0.047	0.96 (0.66, 1.41)	0.85	0.82 (0.55, 1.21)	0.31
Steroid	352 (0.37)	3 (0.49)	1.35	0.61	1.20 (0.17, 8.63)	0.86	1.03 (0.14, 7.53)	0.97
Median (IQR) Biomarker at baseline								
Total cholesterol	5.62 (4.91-6.34)	5.55 (4.80-6.26)	0.94	0.10	0.94 (0.82, 1.08)	0.40	0.98 (0.85, 1.12)	0.76
HDL cholesterol	1.39 (1.17-1.67)	1.41 (1.17-1.70)	1.05	0.20	0.88 (0.76, 1.03)	0.11	0.98 (0.84, 1.16)	0.84

Cystatin C	0.88 (0.80-0.97)	0.90 (0.80-1.01)	1.17	< 0.0001	1.08 (0.94, 1.24)	0.29	0.96 (0.83, 1.12)	0.59
HbA1c	35.10 (32.60-37.60)	35.70 (33.38-38.60)	1.18	< 0.0001	1.19 (1.07, 1.33)	0.001	1.11 (0.99, 1.25)	0.07
CRP	1.27 (0.64-2.59)	1.50 (0.81-3.05)	1.14	< 0.0001	1.11 (1.00, 1.24)	0.04	1.03 (0.91, 1.16)	0.67
Rheumatoid factor	3.16 (3.16-3.16)	3.16 (3.16-3.16)	1.04	0.22	1.03 (0.91, 1.15)	0.68	1.02 (0.90, 1.15)	0.76
Red cell distribution width	13.30 (12.89-13.80)	13.37 (12.95-13.91)	1.10	0.01	1.12 (0.99, 1.27)	0.07	1.07 (0.95, 1.22)	0.27
White cell count	6.63 (5.64-7.80)	6.93 (5.90-8.10)	1.15	0.0002	1.15 (1.01, 1.32)	0.03	1.07 (0.93, 1.23)	0.32
Neutrophil count	4.01 (3.26-4.92)	4.19 (3.52-5.13)	1.12	0.003	1.11 (0.97, 1.27)	0.12	1.04 (0.91, 1.19)	0.57
Lymphocyte count	1.88 (1.52-2.29)	1.92 (1.60-2.35)	1.09	0.02	1.07 (0.94, 1.23)	0.28	1.02 (0.89, 1.17)	0.76
Monocyte count	0.45 (0.37-0.57)	0.46 (0.37-0.58)	1.06	0.13	1.11 (0.97, 1.28)	0.12	1.06 (0.92, 1.21)	0.43

Variables with associations denoted "-" are excluded from the model due to non-significance in previous model and /or collinearity with another variable.

Model 1 adjusts for age, sex, ethnicity, deprivation

Model 2 adjusted for model 1 plus BMI, FEV1, and walking pace

Ho FK, *et al. BMJ Open* 2020; 10:e040402. doi: 10.1136/bmjopen-2020-040402

Supplementary table 3. Linearity of continuous variables with COVID-19 and influenza.

	Covid-19			Pneumonia (after 2016)			Influenza (after 2016)		
	χ^2	df	$P_{nonlinear}$	χ^2	df	$P_{nonlinear}$	χ^2	df	$P_{nonlinear}$
Age	16.32	2.00	0.0003	2.36	1.99	0.30	1.99	2.00	0.37
Deprivation index	0.01	0.07	0.15	2.63	2.00	0.27	2.01	2.00	0.37
BMI	0.85	1.98	0.65	4.93	2.00	0.08	0.00	0.03	0.08
Body fat-free mass	5.44	1.98	0.06	5.45	1.92	0.06	1.32	2.00	0.52
Body fat mass	2.74	1.99	0.25	2.30	1.99	0.31	0.96	2.00	0.62
Body fat percent	0.66	1.97	0.71	1.00	2.00	0.61	0.00	0.02	0.09
Systolic blood pressure	1.65	2.00	0.44	0.84	1.99	0.65	2.24	2.00	0.33
Diastolic blood pressure	2.55	1.99	0.28	0.14	1.51	0.86	1.60	1.97	0.44
FEV1	1.04	1.98	0.59	8.50	1.92	0.01	4.07	1.94	0.12
FVC	0.00	0.03	0.08	8.95	1.95	0.01	1.42	1.99	0.49
FEV1/FVC	3.23	1.98	0.20	3.10	1.83	0.19	0.62	2.00	0.73
Grip strength	7.05	2.00	0.03	2.75	1.93	0.24	0.37	2.00	0.83
Biomarker at baseline									
Total cholesterol	7.40	1.95	0.02	0.94	1.99	0.62	2.50	1.99	0.28
HDL cholesterol	0.00	0.03	0.08	2.69	1.94	0.25	1.89	1.98	0.38
Cystatin C	3.02	1.99	0.22	1.31	1.99	0.52	1.05	1.99	0.59
HbA1c	6.23	2.00	0.04	3.71	2.00	0.16	4.93	2.00	0.08
CRP	11.86	1.99	0.003	7.84	1.99	0.02	5.08	2.00	0.08
Rheumatoid factor	2.22	2.00	0.33	4.24	2.00	0.12	1.70	1.99	0.43
Red cell distribution width	0.55	1.99	0.76	5.07	2.00	0.08	3.64	1.95	0.16
White cell count	5.25	2.00	0.07	1.43	2.00	0.49	0.00	0.03	0.08
Neutrophil count	3.51	1.97	0.17	1.33	2.00	0.51	2.44	1.94	0.28
Lymphocyte count	2.85	1.91	0.23	1.13	2.00	0.57	13.41	2.00	0.001
Monocyte count	0.01	0.04	0.11	1.14	1.76	0.50	9.36	1.91	0.008

Supplementary table 4. Association of risk factors for COVID-19 and pneumonia in UK Biobank with body fat percent replacing BMI in Model 2

	COVID-19		Pneumonia (after 2016)	
	RR (95% CI)	P	RR (95% CI)	P
Age	1.02 (0.95, 1.09)	0.63	1.11 (1.00, 1.23)	0.048
Male	2.72 (1.89, 3.93)	< 0.0001	1.05 (0.67, 1.63)	0.84
Ethnicity				
White	1 (Reference)		1 (Reference)	
Black	2.01 (1.13, 3.55)	0.02	1.22 (0.38, 3.94)	0.74
South Asian	1.60 (0.87, 2.93)	0.13	0.63 (0.19, 2.03)	0.44
Others	1.67 (0.98, 2.86)	0.06	0.51 (0.12, 2.12)	0.36
Deprivation index	1.18 (1.07, 1.29)	0.0009	1.04 (0.90, 1.20)	0.57
Current/former smoker	1.38 (1.12, 1.69)	0.002	0.89 (0.67, 1.18)	0.42
Body fat percent	1.42 (1.23, 1.65)	< 0.0001	1.20 (0.98, 1.46)	0.08
FEV1	0.84 (0.73, 0.97)	0.02	0.75 (0.61, 0.92)	0.005
Walking pace				
Slow	1.61 (1.18, 2.18)	0.002	1.66 (1.08, 2.55)	0.02
Average	1 (Reference)		1 (Reference)	
Brisk	0.95 (0.75, 1.19)	0.64	0.87 (0.63, 1.20)	0.39
grip strength	0.97 (0.83, 1.13)	0.71	0.98 (0.78, 1.24)	0.88
Prevalent disease at baseline				
Longstanding illness	1.16 (0.93, 1.44)	0.18	1.40 (1.04, 1.89)	0.02
Diabetes	1.30 (0.91, 1.85)	0.15	1.65 (1.02, 2.68)	0.04
CVD	1.22 (0.84, 1.76)	0.29	1.35 (0.81, 2.25)	0.25
Cancer	1.20 (0.83, 1.74)	0.33	1.72 (1.13, 2.60)	0.01
Depression	1.01 (0.77, 1.32)	0.94	0.86 (0.59, 1.25)	0.42
Asthma	1.05 (0.78, 1.41)	0.76	1.25 (0.86, 1.83)	0.25
COPD	1.77 (0.55, 5.67)	0.34	-	-
Bronchitis	1.24 (0.60, 2.55)	0.56	1.75 (0.76, 4.03)	0.19
Pneumonia	1.50 (0.74, 3.06)	0.26	1.46 (0.54, 3.98)	0.46
Other respiratory disease	1.20 (0.38, 3.79)	0.76	0.75 (0.10, 5.48)	0.78
Medication at baseline				
Statin	1.25 (0.98, 1.61)	0.08	0.92 (0.63, 1.34)	0.67
BP medication	1.35 (1.05, 1.72)	0.02	0.83 (0.56, 1.23)	0.35
Steroid	0.52 (0.07, 3.82)	0.52	1.03 (0.14, 7.51)	0.98
Biomarker				
Total cholesterol	0.92 (0.83, 1.01)	0.09	0.97 (0.85, 1.11)	0.67
HDL cholesterol	0.82 (0.72, 0.93)	0.002	0.97 (0.83, 1.14)	0.70
Cystatin C	1.14 (1.03, 1.26)	0.009	0.97 (0.84, 1.13)	0.72
HbA1c	1.15 (1.06, 1.24)	0.0003	1.13 (1.00, 1.26)	0.04
CRP	1.04 (0.95, 1.13)	0.38	1.04 (0.92, 1.17)	0.56
Rheumatoid factor	1.01 (0.92, 1.11)	0.85	1.02 (0.90, 1.15)	0.77
Red cell distribution width	1.04 (0.95, 1.14)	0.43	1.08 (0.95, 1.23)	0.23
White cell count	1.04 (0.94, 1.15)	0.43	1.08 (0.94, 1.23)	0.29
Neutrophil count	1.03 (0.93, 1.13)	0.62	1.04 (0.91, 1.20)	0.54
Lymphocyte count	1.03 (0.94, 1.14)	0.51	1.03 (0.90, 1.17)	0.71
Monocyte count	1.01 (0.92, 1.12)	0.77	1.06 (0.92, 1.22)	0.41

Data presented as risk ratios and their 95% CI. continuous exposures were standardised and presented as 1-standard deviation (SD) increment.

Adjusted for age, sex, ethnicity, deprivation, body fat %, FEV1, and walking pace

Supplementary Figure 1. CONSORT diagram.

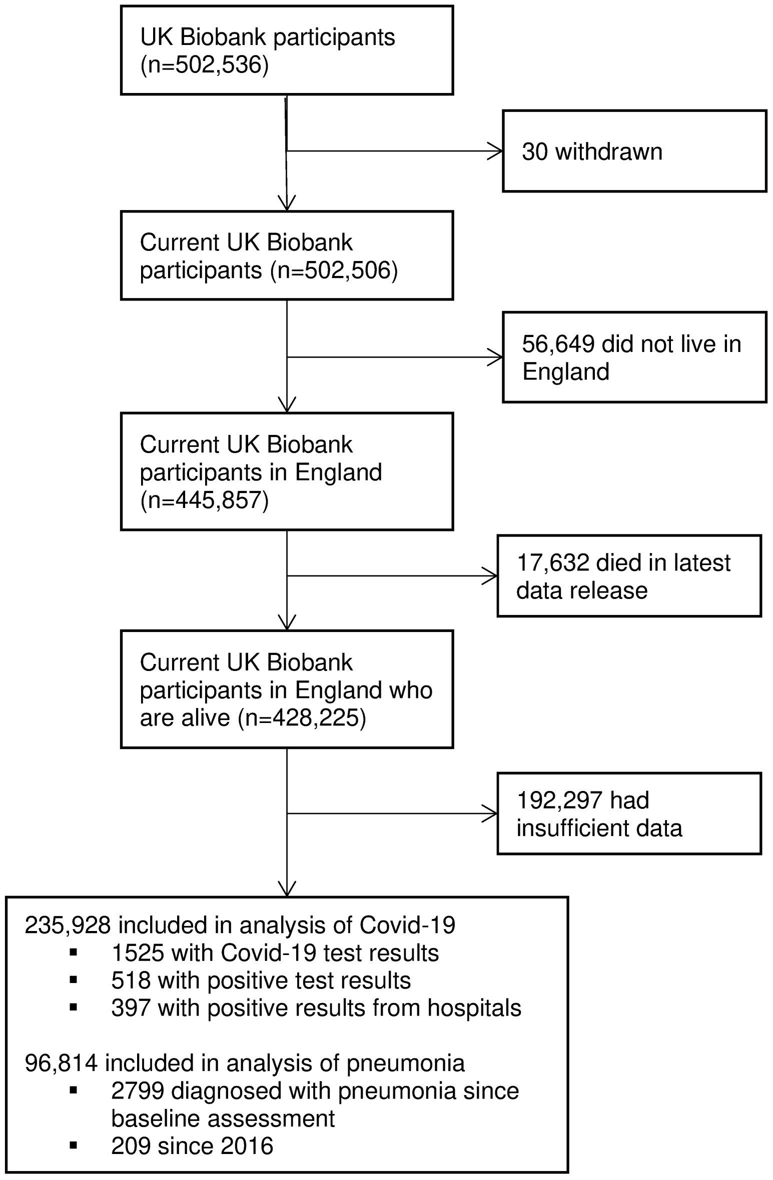

Supplement: Supplementary data [file bmjopen-2020-040402supp001.pdf]
