# Supplementary material for: Modifiable and non-modifiable risk factors for COVID-19, and comparison to risk factors for influenza and pneumonia: results from a UK Biobank prospective cohort study
Source: BMJ Open. 2020 Nov 19;10(11):e040402. doi: 10.1136/bmjopen-2020-040402 (PMC7678347; doi:10.1136/bmjopen-2020-040402)
Supplement: Author's manuscript [file bmjopen-2020-040402.draft_revisions.pdf]

BMJ Open is committed to open peer review. As part of this commitment we make the peer review history of every article we publish publicly available.

When an article is published we post the peer reviewers' comments and the authors' responses online. We also post the versions of the paper that were used during peer review. These are the versions that the peer review comments apply to.

The versions of the paper that follow are the versions that were submitted during the peer review process. They are not the versions of record or the final published versions. They should not be cited or distributed as the published version of this manuscript.

BMJ Open is an open access journal and the full, final, typeset and author-corrected version of record of the manuscript is available on our site with no access controls, subscription charges or pay-per-view fees (http://bmjopen.bmj.com).

If you have any questions on BMJ Open's open peer review process please email info.bmjopen@bmj.com

BMJ Open

Modifiable and non-modifiable risk factors for COVID-19: results from UK Biobank

Journal:	*BMJ Open*
Manuscript ID	bmjopen-2020-040402
Article Type:	Original research
Date Submitted by the Author:	12-May-2020

Complete List of Authors:	Ho, Frederick; University of Glasgow, Institute of Health and Wellbeing<br>Celis-Morales, Carlos; University of Glasgow Institute of Cardiovascular and Medical Sciences<br>Gray, Stuart; University of Glasgow, Institute of Cardiovascular and Medical Sciences, BHF Glasgow Cardiovascular Research Centre<br>Katikireddi, Srinivasa; University of Glasgow School of Life Sciences, MRC/CSO Social & Public Health Sciences Unit<br>Niedzwiedz, Claire; University of Glasgow, Institute of Health & Wellbeing<br>Hastie, Claire; University of Glasgow, Institute of Health and Wellbeing<br>Ferguson, Lyn; University of Glasgow, Institute of Cardiovascular and Medical Sciences<br>Berry, Colin; University of Glasgow, Institute of Cardiovascular and Medical Sciences, British Heart Foundation Glasgow Cardiovascular Research Centre<br>Mackay, Daniel; University of Glasgow, Public Health Unit<br>Gill, Jason; University of Glasgow, Institute of Cardiovascular and Medical Sciences<br>Pell, J. P.; University of Glasgow, Public Health Unit<br>Sattar, Naveed; University of Glasgow, Institute of Cardiovascular and Medical Sciences<br>Welsh, Paul; University of Glasgow, Institute of Cardiovascular and Medical Sciences

Keywords:	Epidemiology < INFECTIOUS DISEASES, Cardiology < INTERNAL MEDICINE, Diabetes & endocrinology < INTERNAL MEDICINE

SCHOLARONE™
Manuscripts

I, the Submitting Author has the right to grant and does grant on behalf of all authors of the Work (as defined in the below author licence), an exclusive licence and/or a non-exclusive licence for contributions from authors who are: i) UK Crown employees; ii) where BMJ has agreed a CC-BY licence shall apply, and/or iii) in accordance with the terms applicable for US Federal Government officers or employees acting as part of their official duties; on a worldwide, perpetual, irrevocable, royalty-free basis to BMJ Publishing Group Ltd ("BMJ") its licensees and where the relevant Journal is co-owned by BMJ to the co-owners of the Journal, to publish the Work in this journal and any other BMJ products and to exploit all rights, as set out in our licence.

The Submitting Author accepts and understands that any supply made under these terms is made by BMJ to the Submitting Author unless you are acting as an employee on behalf of your employer or a postgraduate student of an affiliated institution which is paying any applicable article publishing charge ("APC") for Open Access articles. Where the Submitting Author wishes to make the Work available on an Open Access basis (and intends to pay the relevant APC), the terms of reuse of such Open Access shall be governed by a Creative Commons licence – details of these licences and which Creative Commons licence will apply to this Work are set out in our licence referred to above.

Other than as permitted in any relevant BMJ Author's Self Archiving Policies, I confirm this Work has not been accepted for publication elsewhere, is not being considered for publication elsewhere and does not duplicate material already published. I confirm all authors consent to publication of this Work and authorise the granting of this licence.

For peer review only

Modifiable and non-modifiable risk factors for COVID-19: results from UK Biobank

Frederick K Ho* (0000-0001-7190-9025), Carlos A Celis-Morales* (0000-0003-2612-3917), Stuart R Gray (0000-0001-8969-9636), S Vittal Katikireddi (0000-0001-6593-9092), Claire L Niedzwiedz (0000-0001-6133-4168), Claire Hastie (0000-0002-4604-3319), Lyn D Ferguson (0000-0002-6136-8349), Colin Berry (0000-0002-4547-8636), Daniel F Mackay (0000-0001-5381-7098), Jason MR Gill (0000-0003-3615-0986), Jill P Pell** (0000-0002-8898-7035), Naveed Sattar MD** (0000-0002-1604-2593), Paul Welsh** (0000-0002-7970-3643)

*Joint first authors

**Joint senior

Affiliations

Institute of Health and Wellbeing, University of Glasgow, 1 Lilybank Gardens, Glasgow, G12 8RZ, United Kingdom Frederick K Ho research associate, S Vittal Katikireddi clinical senior research fellow, Claire L Niedzwiedz RCUK innovation fellow, Claire Hastie research associate, Daniel F Mackay professor of public health informatics, Jill P Pell director of institute

Institute of Cardiovascular and Medical Sciences, University of Glasgow, BHF Glasgow Cardiovascular Research Centre, 126 University Place, Glasgow, G12 8TA, United Kingdom Carlos A Celis-Morales research fellow, Stuart R Gray senior lecturer, Lyn D Ferguson clinical lecturer, Colin Berry professor of cardiology and imaging, Jason MR Gill professor of cardiometabolic health, Naveed Sattar professor of metabolic medicine, Paul Welsh senior lecturer

Correspondence to:

Paul Welsh

Institute of Cardiovascular and Medical Sciences, University of Glasgow, BHF Glasgow Cardiovascular Research Centre, 126 University Place, Glasgow G12 8TA, UK.

Tel: +44 (0)141 330 1722, Email address: paul.welsh@glasgow.ac.uk

ORCID ID: 0000-0002-7970-3643

For peer review only - http://bmjopen.bmj.com/site/about/guidelines.xhtml

Abstract

Objectives

We aimed to investigate demographic, lifestyle, socioeconomic, and clinical risk factors, and compared them to risk factors for pneumonia and influenza in UK Biobank.

Design

Cohort study

Setting

UK Biobank

Participants

49-83 year olds (in 2020) from a general population study

Main Outcome Measures

Confirmed COVID-19 infection (positive SARS-CoV-2 test). Incident influenza and pneumonia were obtained from primary care data. Poisson regression was used to study the association of exposure variables with outcomes.

Results

Among 235,928 participants, 397 had confirmed COVID-19. After multivariable adjustment, modifiable risk factors were higher body mass index and higher HbA1C (RR 1.28 and RR 1.14 per SD increase, respectively), lower FEV1 (RR 0.84 per SD increase), smoking (RR 1.39), slow walking pace as a proxy for physical fitness (RR 1.53), HDL-cholesterol (RR 0.83 per SD increase), and use of blood pressure medications as a proxy for hypertension (RR 1.33). Non-modifiable risk factors included male sex (RR 1.72), black ethnicity (RR 2.00), socioeconomic deprivation (RR 1.17 per SD increase in Townsend Index), and high cystatin C (RR 1.13 per SD increase). The risk factors overlapped with pneumonia somewhat; less so for influenza. The associations with modifiable risk factors were generally stronger for COVID-19, than pneumonia or influenza.

Conclusion

These findings suggest that modification of lifestyle may help to reduce the risk of COVID-19 and could be a useful adjunct to other interventions, such as social distancing and shielding of high risk.

Funding

British Heart Foundation, Medical Research Council, Chief Scientist Office.

For peer review only - http://bmjopen.bmj.com/site/about/guidelines.xhtml

For peer review only

Strengths and limitations

- Large cohort from a general population, as opposed to a cohort of hospitalised patients, at an age relevant to more severe COVID-19 symptoms.
- Biochemistry assays performed in a single dedicated central laboratory.
- Extensively emerging and novel risk factors for COVID-19.
- UK Biobank is not representative of the whole UK population.
- Exposures were measured several years before the development of the outcomes, and misclassification of the exposures will likely bias our results to the null.

For peer review only

For peer review only - http://bmjopen.bmj.com/site/about/guidelines.xhtml

Introduction

COVID-19, caused by SARS-CoV-2 infection, includes a spectrum of morbidity from asymptomatic infection[1] to severe pneumonia in patients presenting for medical care.[2] The COVID-19 pandemic[3] has led to concerted research efforts to identify people at greatest risk of developing the infection and progressing to critical illness and dying. Predictors of disease severity include older age, smoking, diabetes, hypertension, kidney disease, COPD, and previous cardiovascular disease.[4–7] In addition it is becoming clear that other risk factors might include obesity and low physical fitness.[8,9] However, many studies investigate risk factors for disease progression to death or critical illness among hospitalised patients[7] as opposed to healthy comparators.

Identifying the risk factors for COVID-19 is important in terms of identifying factors that can be modified to reduce risk, as well as identifying non-modifiable risk factors that can help identify high risk groups who require shielding and targeting for testing, and eventual vaccine and anti-viral therapies. Pneumonia is a life-threatening complication of COVID-19 infection.[10] Established major risk factors for community-acquired pneumonia include many of the emerging risk factors for COVID-19.[11] As COVID-19 is caused by SARS-CoV-2 viral infection, it may have risk factors for contraction of the disease that are common to other respiratory virus conditions such as Influenza.

UK Biobank is a large prospective, deeply-phenotyped, population-based cohort study carried out in the UK.[12] Over the study period, testing for COVID-19 in England was conducted in A&E departments and in-hospital. These data were provided by Public Health England (PHE) and linked to UK Biobank baseline data. We aimed to establish modifiable and non-modifiable risk factors for confirmed COVID-19. We also aimed to compare these risk factors to risk factors for the incident pneumonia over a similar time span.

Methods

UK Biobank was conducted via 22 assessment centres across England, Scotland, and Wales between March 2006 and December 2010 and recruited 502,624 participants aged 37 to 73

For peer review only - http://bmjopen.bmj.com/site/about/guidelines.xhtml

years. The present study was restricted to participants living in England for whom COVID-19 test results were available. Death data were available to the end of January 2018 in England. To reduce bias, we excluded from the study all participants known to have died COVID-19 pandemic. Baseline biological measurements were recorded and touch-screen questionnaires were administered according to a standardised protocol.[12,13] UK Biobank received ethical approval from the North West Multi-Centre Research Ethics Committee (REC reference: 11/NW/03820). All participants gave written informed consent before enrolment in the study, which was conducted in accord with the principles of the Declaration of Helsinki. This study was performed under UK Biobank application number 7155.

Outcomes

Results of COVID-19 tests for UK Biobank participants were provided by PHE.[7] Data provided by PHE included the specimen date, specimen type (e.g. upper respiratory tract), laboratory, origin (whether evidence from microbiological record that the participant was an inpatient or not) and result (positive or negative). Data were available for the period 16th March 2020 to 3rd May 2020. Results were available for 5,356 tests conducted on 3,003 individuals. Confirmed COVID-19 infection (primary outcome) was defined as at least one positive result in the context of an in-hospital or accident and emergency (A&E) test. Any positive test result was used as a sensitivity analysis.

Pneumonia was defined based on ICD-10 codes J12-J18, and influenza based on J09-J11, converted into Read Codes using the UK Biobank's look-up table. Incident pneumonia and influenza, occurring after 1st January 2016 (an arbitrary date taken to mimic the time lag from baseline exposure measurement to incident COVID-19 infection, while also obtaining sufficient case numbers) was obtained from a 41% sample of participants with available data from primary care. A sensitivity analysis was conducted using cases after 1st January 2015 and demonstrated consistent data. An analysis was also conducted exploring risk factors for all cases of pneumonia and influenza after baseline.

Exposures

For peer review only - http://bmjopen.bmj.com/site/about/guidelines.xhtml

Exposures were measured at the baseline assessment visit between 2006 and 2010. Current age (on 1st March 2020) was derived from the assessment date and age at recruitment. Ethnicity, smoking, alcohol consumption, physician-diagnosed prevalent conditions and medication use were self-reported. For the present analyses, ethnicity was coded as white, south Asian, black, or mixed/other. Smoking status was categorised into never versus former/current smoking. Systolic and diastolic blood pressures were measured at the baseline visit, preferentially using an automated measurement, but using manual measurement where this was not available, and average of available measures used. Area-level socioeconomic deprivation was assessed by the Townsend score (incorporating measures of unemployment, non-car ownership, non-home ownership and household overcrowding) corresponding to the participants' home postcode. Higher scores on the Townsend score represent greater socioeconomic deprivation. Self-reported walking pace was rated by each participant as slow, steady/average, or brisk.[14]

The definition of baseline diabetes included self-reported type 1 or type 2 diabetes, those with a primary or secondary hospital diagnoses relating to diabetes at baseline (ICD-10 codes E10-E14.9), and those who reported using diabetes medications. Baseline cardiovascular disease was defined as self-reported myocardial infarction, stroke, or transient ischaemic attack. Cancer, longstanding illness, and depression were self-reported on touchscreen questionnaire. Other previous health complaints including asthma, rheumatoid arthritis, CKD, systemic lupus erythematosus (SLE), sleep apnoea, COPD, pneumonia, bronchitis (including bronchitis, bronchiectasis, and emphysema), and other respiratory diseases (including interstitial lung disease, asbestosis, pulmonary fibrosis, alveolitis, respiratory failure, pleurisy, pneumothorax, other respiratory condition) which were derived from self-report at nurse interview. Some conditions did not have sufficient numbers of cases for multivariable analysis, but univariable results are presented for completeness. Statin (categorised to include other cholesterol lowering medications) and blood pressure medication use were also recorded from self-report, with blood pressure medication being used as a proxy for baseline diagnosed hypertension.

BMI is the ratio of the measured body mass in kg divided by height squared measured in metres. Height was measured using a Seca 202 height measure. Weight and whole-body fat

For peer review only - http://bmjopen.bmj.com/site/about/guidelines.xhtml

mass and fat free mass were measured to the nearest 0.1 kg using the Tanita BC-418 MA body composition analyser. Socks and shoes were removed when height was measured Grip strength was measured using a Jamar J00105 hydraulic hand dynamometer and the mean was derived from the right and left hand values expressed in kilograms.[15]

Lung function was assessed by spirometry using a Vitalograph Pneumotrac 6800 spirometer (Vitalograph, Buckingham, UK). Participants did not perform spirometry if they answered yes to unsure to the following: chest infection in the last month, history of detached retina, heart attack, surgery to eyes, chest or abdomen in last 3 months, history of collapsed lung, pregnancy, or currently on medication for tuberculosis. The aim was to record two acceptable blows from a maximum of three attempts. The spirometer software compared the acceptability of the first two blows and, if acceptable (defined as a ≤5% difference in FVC and FEV1), the third blow was not required. The mean observation was taken for both measures.

Blood collection sampling procedures for the study have been previously described and validated.[16] Biochemical assays were performed at a dedicated central laboratory on around 480,000 samples. Further details of these measurements can be found in the UK Biobank Data Showcase and Protocol (http://www.ukbiobank. ac.uk). For the present study we included total cholesterol, HDL cholesterol, rheumatoid factor, cystatin C, glycated haemoglobin (HbA1C), C-reactive protein (CRP), differential white blood cell count, and red cell distribution width as exposures of interest.

Statistics

Mean and standard deviations were reported for continuous outcomes except for biomarkers, where median and interquartile ranges (IQR) were reported. Poisson regression with robust 'sandwich' standard errors were used to study the associations of exposure variables with confirmed COVID-19 and pneumonia. Poisson regressions were used because they provide risk ratio (RR) which is easier to interpret and robust error estimation ensures accurate inference.[17] Three adjustment schemes were considered: model 0 - univariate (i.e. no adjustment), model 1 – adjusted for age, sex, ethnicity, and deprivation index, and model 2 – further adjusted for behavioural (smoking and alcohol drinking) and physical

For peer review only - http://bmjopen.bmj.com/site/about/guidelines.xhtml

(adiposity, blood pressure, spirometry, and physical capability) factors that were found to be significant in model 1. To avoid multicollinearity, if there were similar factors that were significant in model 2, only the one with strongest association was chosen. This included, for example, choosing one from, BMI, BMI categories, body fat-free mass, body fat mass, and body fat percent, or one from FEV1, FVC, and FEV1/FVC. For continuous variables, the linearity of exposure-outcome associations were tested using penalised cubic splines in generalised additive model (GAM).[18] Nonlinearity was tested using likelihood ratio test comparing a model with the exposure fitted on a spline with a model assuming a linear exposure-outcome relationship. P-value for nonlinearity < 0.05 suggest evidence against the linearity assumption. Spline smoothness was chosen using generalised cross validation.[19] Population attributable fractions (PAFs) were calculated to determine the relative contribution of each risk factor to the overall number of confirmed COVID-19 cases within UK Biobank. Another Poisson model which included all significant factors in model 2 was fitted to estimate the mutually adjusted RRs. These RRs were biased towards null because of over adjustment bias but were used to construct the PAFs to ensure the PAF estimates did not overlap or exceed 100%. In general, two-tailed P-values < 0.05 were considered statistically significant. Analyses were conducted in R Statistical Software version 3.5.3 with the package "mgcv".

Results

Of 445,857 participants in England, 428,225 were alive during the available follow-up period. Complete data on covariates were available for 235,928(55.1%) participants. Primary care data on incident pneumonia were available in 96,814 (41.0%) participants (Supplementary Fig 1). At 1st March 2020, the age range of eligible participants was 49-83 years, and time elapsed from baseline was median 10.97 years (IQR 10.36-11.55 years). Of these participants, 1,525 received at least one COVID-19 test, and 518 had at least one positive result, with 397 positive results conducted in hospital or A&E (primary outcome).

Univariable risk factors for incident COVID-19

Key univariable potentially modifiable risk factors for confirmed COVID-19 included current and former smoking (RR 1.56), higher BMI, body fat, and HbA1C (RR 2.32 for obesity), poor

For peer review only

lung function (RR 0.84 for 1 SD increase in FEV1), treated hypertension (RR 1.89), HDL-cholesterol (RR 0.71 per SD increase) and slow walking pace (RR 2.29) (Table 1). Among non-modifiable risk factors were older age (particularly the over 75s), male sex (RR 1.38), black ethnicity (RR 2.88), socioeconomic deprivation (RR 1.30 per SD increase in Townsend Index) (Table 1). Among baseline comorbidities, general long standing illness (RR 1.59), baseline diabetes (RR 2.16), baseline CVD (RR 1.93), sleep apnoea (RR 3.32), statin use (RR 1.80), higher inflammatory markers (particularly white blood cell count; RR 1.17 per SD increase), and higher cystatin C (RR 1.34 per SD increase) were also associated with confirmed COVID-19 (Table 1). Risk factor associations were similar when all 518 test positive COVID-19 cases were considered, rather than only in-hospital positive tests (supplementary table 1)

Univariable risk factors for incident pneumonia

Of the 96,814 participants with primary care data, 209 had pneumonia recorded after 2016. Of the modifiable risk factors pneumonia was associated with BMI, body fat and HbA1C (more moderately than COVID-19), lung function (more strongly than COVID-19), and slow walking pace, but not smoking, lipids, blood pressure, or blood pressure medication use. Incident pneumonia was more common in participants who were over 65 years in 2020, and in women (RR 0.59 among men) (Table 1). Ethnicity was not associated with pneumonia. Among comorbidities, baseline diabetes, CVD and cancer were approximately twice as common in those who developed pneumonia (Table 1), and any longstanding illness was also strong associated with pneumonia. Inflammatory markers were similarly associated with pneumonia as with COVID-19.

When investigating risk factors for pneumonia over the full follow-up time from baseline, risk factors were generally similar although there was increased power due to a higher number of incident cases (Supplementary table 1). In this analysis smoking was weakly associated with pneumonia (RR 1.15) as was blood pressure medication use (RR 1.13). Similar findings were found when we studied pneumonia occurring after 2015 (Supplementary Table 2).

Univariable risk factors for incident influenza

For peer review only - http://bmjopen.bmj.com/site/about/guidelines.xhtml

Of the 96,814 participants with primary care data, 94 had influenza recorded after 2016. Those who developed influenza were generally slightly more socioeconomically deprived, more likely to have bronchitis at baseline, and less likely to take statins. No other risk factors showed strong associations (Table 1). When all influenza cases occurring after baseline were considered, evidence of statistically significant associations became stronger due to increased power. Cases were more common in younger people and in women. Black people and South Asians were at increased risk. Smoking, poor lung function, slow walking pace, and high BMI were all associated with higher influenza risk (Supplementary Table 1).

Multivariable models for COVID-19 and pneumonia

After multivariable adjustment for sociodemographic factors, the modifiable risk factors for confirmed COVID-19 included smoking (RR 1.45 (95% CI 1.19-1.79)), higher BMI (RR 1.36 per SD increase (95% CI 1.25-1.48)) and other measures of body fat, higher HbA1C (RR 1.23 per 1 SD increase (95% CI 1.15-1.32)), as well as blood pressure medication, FEV1 and FVC , and slow walking pace (RR 1.99 (95% CI 1.48-2.68)) (Model 1, Table 2). Other risk factors were older age (RR 1.12 per 5 years (95% CI 1.05-1.19)), male sex (RR 1.36 (95% CI 1.11-1.66)), black ethnicity (RR 2.32 (95% CI 1.33, 4.04)), South Asian ethnicity (RR 1.98 (95%CI 1.10-3.55)) socioeconomic deprivation (RR 1.27 per SD increase in Townsend Index (95% CI 1.16-1.39)). Among comorbidities, risk factors included longstanding illness (RR 1.44 (95% CI 1.17-1.77)), diabetes, CVD and statin use. Among blood biomarkers, risk factors included lower total and HDL-cholesterol, higher cystatin C (RR 1.27 per 1 SD increase (95% CI 1.16-1.39)), CRP and white blood cell count (Table 2).

After adding BMI, blood pressure, FEV1 and walking pace as mediators/covariates to the adjustment model, modifiable risk factors for COVID-19 admission continued to include smoking (RR 1.39 (95% CI 1.13-1.73)), higher BMI (RR 1.28 (95% CI 1.16-1.40)), higher HbA1C (RR 1.14 (956%CI 1.05,1.23), treated hypertension (RR 1.33 95% CI 1.04-1.70) and slow walking pace (RR 1.53 (95% CI 1.12-2.08)). The age association was no longer significant after these additional adjustments. Other risk factors included male sex (RR 1.72 (95% CI 1.25-2.35)), black ethnicity (RR 2.00 (95% CI 1.16, 3.53)), socioeconomic deprivation (RR 1.17 (95% CI 1.06-1.29)), lower HDL-cholesterol (RR 0.83 (95% CI 0.73-0.95)), and higher cystatin C (RR 1.13 (95% CI 1.02-1.25)) (Model 2, Table 2).

For peer review only - http://bmjopen.bmj.com/site/about/guidelines.xhtml

Nonlinear associations of continuous variables with COVID-19 and pneumonia are shown in Figure 1 (and supplementary table 3). Age was associated with COVID-19 admission in a J-shaped curve, where participants aged 60-70 years shared similar risk and participants aged over 75 years had exponentially increased risk with age. The associations of BMI and FEV1 with COVID-19 were fairly linear. Associations were similar when adjusting for body fat percentage rather than BMI (Supplementary Table 3).

Directly contrasting model 2 for COVID-19 and pneumonia (Figure 2), the risk factors in common for both conditions were higher BMI and slow walking pace, although less strongly associated with pneumonia than for COVID-19. Highlighting specific differences, smoking and treated blood pressure were only associated with COVID-19. Pneumonia was more common in women, and socioeconomic deprivation, HDL cholesterol, cystatin-C, and HbA1c were not associated with pneumonia. Low FEV1 was more strongly associated with pneumonia than with COVID-19. Pneumonia also showed an association with baseline cancer.

Population attributable risks

Factors that were significant in Table 2 were mutually adjusted to compare their population attributable fractions for COVID-19 and pneumonia (Table 3). Among potentially modifiable risk factors smoking accounted for 14.9% of COVID-19 cases that occurred within the UK Biobank population, obesity with 6.3%, high HbA1C with 5.3%, treated BP with 5.1%, and slow walking pace with 4.0% (total 35.6%). In contrast, none of these factors were large contributors to pneumonia cases within UK Biobank. (Table 3)

Discussion

In this population-based study, we found that confirmed COVID-19 infection was associated with a number of modifiable risk factors, a trend that less apparent for pneumonia and influenza. In particular, the associations of smoking, BMI (and body fat), hypertension, and physical fitness (as measured by slow walking pace) with COVID-19 are of note; even when

For peer review only - http://bmjopen.bmj.com/site/about/guidelines.xhtml

such factors were measured a decade before infection they potentially accounted for one-third of COVID-19 cases in UK Biobank. The associations of FEV1 and BMI with COVID-19 are linear, suggesting even modest improvements in lifestyle may be beneficial to risk of presumed severe COVID symptoms. The other independent risk factors for COVID-19 infection included older age, male sex, black ethnicity, socioeconomic deprivation, longstanding illness, and reduced renal function as measured by cystatin C, the latter also notable given renal complication in severe COVID-19.

It is important to recognise the competing risk for health of lockdown and social distancing. Social distancing reduces viral transmission, but also has consequences for lifestyle. Previous data suggests that physical fitness can be rapidly lost when activity levels decrease,[20,21] and this will also result in an increase in BMI.[22,23] Further, social distancing may increase loneliness, depression, and psychological stress in some people and consequently adversely affect eating habits[24] and other health behaviours. Anecdotal evidence from our clinics and media suggest many are struggling with overeating. This study strongly suggests public health guidance should focus on reducing the risk of severe complications of COVID-19 by advocating a healthy lifestyle during the ongoing pandemic, not just for general cardiovascular and metabolic health, but also to help to protect against COVID-19 infection, used alongside other public health interventions.

Understanding the actual causes of disease, rather than markers that simply correlate with exposures, is clearly a key issue to consider. The independent association of socioeconomic deprivation with COVID-19 after multiple adjustment may be explained by the accumulation of earlier life socioeconomic adversities which can result in less physiological reserve and more multimorbidity.[24,25] It may also be linked to more overcrowding, reduced social distancing, and potential exposure to greater viral load. Asthma, diabetes and high blood pressure, which have been shown to be associated with a higher risk of severe COVID-19 outcomes[26] also showed some trends to be associated with COVID-19 in this dataset. While substantial focus of COVID-19 research has been its apparently more aggressive symptoms and disease progression in older people, it is important to recognise that people who are older have less cardiorespiratory reserve to cope with COVID-19 infection. Older

For peer review only - http://bmjopen.bmj.com/site/about/guidelines.xhtml

age is also associated with more hypertension and diabetes, poorer lung function, and greater relative fat mass.[24]

Previous reports have suggested that obesity or excess ectopic fat deposition may be a unifying risk factor for severe Covid-19 infection,[8,27] reducing both protective cardiorespiratory reserve as well as potentiating the immune dysregulation that appears, at least in part, to mediate the progression to critical illness in a proportion of COVID-19 patients. Our analysis bears out these hypotheses. Once BMI or body fat was adjusted for, inflammatory markers were no longer associated with incident COVID-19. This suggests proinflammatory markers, arising from increased adipose deposition, are probably acting as a marker for body fat which seems to be an adverse risk factor for severe COVID-19.[8,27–29] Furthermore, obesity enhances thrombosis,[30] which is relevant given the association between severe COVID-19 and pro-thrombotic disseminated intravascular coagulation and high rates of venous thromboembolism,[31,32] as well as the association with D-dimer seen in other reports.[33] D-dimer was not measured in blood samples in UK Biobank.

Given that pneumonia is a critical clinical complication of COVID-19, it is important to understand how risk factors for COVID-19 related pneumonia differ from “classic” community acquired pneumonia.[11] Our comparison between other common respiratory diseases and COVID-19 suggests that identification of at-risk groups based on our understanding of other respiratory diseases may be inadequate – a more refined approach to risk stratification based on COVID-19 specific risks is needed, and future data should focus on some of the exposures we identify to achieve this.

The strengths of our study include the large cohort size at an age relevant to more severe COVID-19 symptoms, and biochemistry assays performed in a single dedicated central laboratory. We were also able to extensively explore emerging and novel risk factors for COVID-19, while simultaneously comparing to risk factors for pneumonia identified to primary care providers. Limitations include that UK Biobank is not representative of the whole UK population,[34] (our data focuses on England specifically) although this is generally not a concern in investigating risk associations.[35] Care should be taken in generalising the PAF estimates. These related to cases occurring within the UK Biobank

For peer review only - http://bmjopen.bmj.com/site/about/guidelines.xhtml

population, but are not directly applicable to the general population where the prevalence of risk factors is different. However, they allowed a direct comparison between COVID-19 and pneumonia in the same cohort. Due to the under-representation of non-white ethnicities in UK Biobank, we have limited power to explore important interactions by ethnic group (although Black ethnicity was associated with the outcome), and we recognise this as an important risk factor. Ascertainment bias, including differential healthcare seeking, differential testing and differential prognosis may explain some differences in outcomes given poor coverage of testing in the UK. It is also likely that cases will generally be at the more severe end of the clinical spectrum by using hospitalised cases as the outcome, although use of admission to ITU would be additionally informative once sufficient case numbers accrue. Despite this, we still observe many similar risk factors to incident pneumonia, which is more likely to have close to complete case ascertainment in primary care records, although, as discussed, other risk factors seem more strongly linked to COVID-19 infections. Exposures were measured several years before the development of the outcomes, and misclassification of the exposures will likely bias our results to the null. However, this also serves to illustrate that the risk factors were generally present many years before development of the disease, and as is well known, risk factors tend to track with age. We have also been unable to fully exclude all deaths that occurred prior to the pandemic, due to lack of up-to-date linkage to mortality records.

In conclusion, these early data from UK Biobank suggest risk factors for confirmed COVID-19 infection differ in some important ways from risk factors for pneumonia, being more common in males than females, in lower SES, and with stronger associations with ethnicity, CV risk markers, prior smoking and adiposity. Such findings suggest possible merit in advocating improvements in lifestyle as an additional measure to reduce the risk of COVID-19 alongside existing public health measures such as social distancing and shielding of high risk groups. have implications for health advice targeted at the public to lessen risks during this pandemic

For peer review only - http://bmjopen.bmj.com/site/about/guidelines.xhtml

Contributors

PW, JPP and NS conceived the idea for the paper. FH conducted the analysis. All authors contributed to the interpretation of the findings. PW, FH, CCM and NS jointly wrote the first draft. All authors critically revised the paper for intellectual content and approved the final version of the manuscript. PW and NS are guarantors of the work. The corresponding author attests that all listed authors meet authorship criteria and that no others meeting the criteria have been omitted.

Transparency Statement

The guarantors affirm that the manuscript is an honest, accurate, and transparent account of the study being reported; that no important aspects of the study have been omitted; and that any discrepancies from the study as originally planned (and, if relevant, registered) have been explained.

Funding

The work in this study is supported by the British Heart Foundation Centre of Research Excellence Grant RE/18/6/34217. CLN acknowledges funding from a Medical Research Council Fellowship (MR/R024774/1). SVK acknowledges funding from the Medical Research Council (MC_UU_12017/13), Scottish Government Chief Scientist Office (SPHSU13), and NRS Senior Clinical Fellowship (SCAF/15/02). The funders played no part in the research.

Competing interests: All authors have completed the ICMJE uniform disclosure form at www.icmje.org/coi_disclosure.pdf and declare: support from funders as acknowledged above for the submitted work; PW has received research grants from Roche Diagnostics, AstraZeneca and Boehringer Ingelheim outside the submitted work, and NS has received grant and personal fees from Boehringer Ingelheim, and personal fees from Amgen, AstraZeneca, Eli Lilly, Novo Nordisk, Pfizer, and Sanofi outside the submitted work; all authors declare no other relationships or activities that could appear to have influenced the submitted work.

Ethics Approval

For peer review only

UK Biobank received ethical approval from the North West Multi-centre Research Ethics Committee (REC reference: 11/NW/03820). All participants gave written informed consent before enrolment in the study, which was conducted in accordance with the principles of the Declaration of Helsinki. Direct dissemination of the results to participants is not possible/applicable.

Data Sharing

UK Biobank data can be requested by bona fide researchers for approved projects, including replication, through https://www.ukbiobank.ac.uk/.

Copyright

I, the Submitting Author has the right to grant and does grant on behalf of all authors of the Work (as defined in the below author licence), an exclusive licence and/or a non-exclusive licence for contributions from authors who are: i) UK Crown employees; ii) where BMJ has agreed a CC-BY licence shall apply, and/or iii) in accordance with the terms applicable for US Federal Government officers or employees acting as part of their official duties; on a worldwide, perpetual, irrevocable, royalty-free basis to BMJ Publishing Group Ltd ("BMJ") its licensees and where the relevant Journal is co-owned by BMJ to the co-owners of the Journal, to publish the Work in BMJ Open and any other BMJ products and to exploit all rights, as set out in our licence.

For peer review only

References

1 Mizumoto K, Kagaya K, Zarebski A, *et al.* Estimating the asymptomatic proportion of coronavirus disease 2019 (COVID-19) cases on board the Diamond Princess cruise ship, Yokohama, Japan, 2020. Eurosurveillance. 2020;**25**. doi:10.2807/1560-7917.ES.2020.25.10.2000180

2 Wu Z, McGoogan JM. Characteristics of and Important Lessons from the Coronavirus Disease 2019 (COVID-19) Outbreak in China: Summary of a Report of 72314 Cases from the Chinese Center for Disease Control and Prevention. *JAMA - J Am Med Assoc* 2020;**323**:1239–42. doi:10.1001/jama.2020.2648

3 Coronavirus disease 2019 (COVID-19) Situation Report-76 HIGHLIGHTS.

4 Petrilli CM, Jones SA, Yang J, *et al.* Factors associated with hospitalization and critical illness among 4,103 patients with COVID-19 disease in New York City. *medRxiv* 2020;:2020.04.08.20057794. doi:10.1101/2020.04.08.20057794

5 Tu H, Tu S, Gao S, *et al.* The epidemiological and clinical features of COVID-19 and lessons from this global infectious public health event. *J Infect* 2020;**0**. doi:10.1016/j.jinf.2020.04.011

6 Banerjee A, Pasea L, Harris S, *et al.* Estimating excess 1- year mortality from COVID-19 according to underlying conditions and age in England: a rapid analysis using NHS health records in 3.8 million adults. *medRxiv* 2020;:2020.03.22.20040287. doi:10.1101/2020.03.22.20040287

7 Richardson S, Hirsch JS, Narasimhan M, *et al.* Presenting Characteristics, Comorbidities, and Outcomes Among 5700 Patients Hospitalized With COVID-19 in the New York City Area. *JAMA* Published Online First: April 2020. doi:10.1001/jama.2020.6775

8 Simonnet A, Chetboun M, Poissy J, *et al.* High prevalence of obesity in severe acute respiratory syndrome coronavirus-2 (SARS-CoV-2) requiring invasive mechanical ventilation. *Obesity (Silver Spring)* Published Online First: 9 April 2020. doi:10.1002/oby.22831

9 Obesity in patients younger than 60 years is a risk factor for Covid-19 hospital admission | Clinical Infectious Diseases | Oxford Academic.

10 2 Treatment and care planning | COVID-19 rapid guideline: managing suspected or confirmed pneumonia in adults in the community | Guidance | NICE.

11 Almirall J, Serra-Prat M, Bolíbar I, *et al.* Risk Factors for Community-Acquired Pneumonia in Adults: A Systematic Review of Observational Studies. *Respiration* 2017;**94**:299–311. doi:10.1159/000479089

12 Collins R. What makes UK Biobank special? *Lancet* 2012;**379**:1173–4. doi:10.1016/S0140-6736(12)60404-8

13 Sudlow C, Gallacher J, Allen N, *et al.* UK Biobank: An Open Access Resource for Identifying the Causes of a Wide Range of Complex Diseases of Middle and Old Age. *PLoS Med* 2015;**12**:e1001779. doi:10.1371/journal.pmed.1001779

14 Celis-Morales CA, Gray S, Petermann F, *et al.* Walking Pace Is Associated with Lower Risk of All-Cause and Cause-Specific Mortality. *Med Sci Sports Exerc* 2019;**51**:472–80. doi:10.1249/MSS.0000000000001795

15 Celis-Morales CA, Welsh P, Lyall DM, *et al.* Associations of grip strength with cardiovascular, respiratory, and cancer outcomes and all cause mortality: Prospective cohort study of half a million UK Biobank participants. *BMJ* 2018;**361**:k1651. doi:10.1136/bmj.k1651

For peer review only - http://bmjopen.bmj.com/site/about/guidelines.xhtml

16 Elliott P, Peakman TC. The UK Biobank sample handling and storage protocol for the collection, processing and archiving of human blood and urine. *Int J Epidemiol* 2008;**37**:234–44. doi:10.1093/ije/dym276

17 Zou G. A modified poisson regression approach to prospective studies with binary data. *Am J Epidemiol* 2004;**159**:702–6. doi:10.1093/aje/kwh090

18 Wood SN. Stable and efficient multiple smoothing parameter estimation for generalized additive models. *J Am Stat Assoc* 2004;**99**:673–86. doi:10.1198/016214504000000980

19 Wood SN, Pya N, Säfken B. Smoothing Parameter and Model Selection for General Smooth Models. *J Am Stat Assoc* 2016;**111**:1548–63. doi:10.1080/01621459.2016.1180986

20 Olsen RH, Krogh-Madsen R, Thomsen C, *et al.* Metabolic responses to reduced daily steps in healthy nonexercising men. JAMA - J. Am. Med. Assoc. 2008;**299**:1261–3. doi:10.1001/jama.299.11.1259

21 Drummond MJ, Dickinson JM, Fry CS, *et al.* Bed rest impairs skeletal muscle amino acid transporter expression, mTORC1 signaling, and protein synthesis in response to essential amino acids in older adults. *Am J Physiol - Endocrinol Metab* 2012;**302**. doi:10.1152/ajpendo.00603.2011

22 Bradbury KE, Guo W, Cairns BJ, *et al.* Association between physical activity and body fat percentage, with adjustment for BMI: A large cross-sectional analysis of UK Biobank. *BMJ Open* 2017;**7**:e011843. doi:10.1136/bmjopen-2016-011843

23 Parsons TJ, Manor O, Power C. Physical activity and change in body mass index from adolescence to mid-adulthood in the 1958 British cohort. *Int J Epidemiol* 2006;**35**:197–204. doi:10.1093/ije/dyi291

24 Razzoli M, Pearson C, Crow S, *et al.* Stress, overeating, and obesity: Insights from human studies and preclinical models. Neurosci. Biobehav. Rev. 2017;**76**:154–62. doi:10.1016/j.neubiorev.2017.01.026

25 Packard CJ, Bezlyak V, McLean JS, *et al.* Early life socioeconomic adversity is associated in adult life with chronic inflammation, carotid atherosclerosis, poorer lung function and decreased cognitive performance: A cross-sectional, population-based study. *BMC Public Health* 2011;**11**:42. doi:10.1186/1471-2458-11-42

26 Zhao X, Zhang B, Li P, *et al. Incidence, clinical characteristics and prognostic factor of patients with COVID-19: a systematic review and meta-analysis*. Cold Spring Harbor Laboratory Press 2020. doi:10.1101/2020.03.17.20037572

27 Lighter J, Phillips M, Hochman S, *et al.* Obesity in patients younger than 60 years is a risk factor for Covid-19 hospital admission. *Clin Infect Dis* Published Online First: 2020. doi:10.1093/cid/ciaa415

28 Timpson NJ, Nordestgaard BG, Harbord RM, *et al.* C-reactive protein levels and body mass index: Elucidating direction of causation through reciprocal Mendelian randomization. *Int J Obes* 2011;**35**:300–8. doi:10.1038/ijo.2010.137

29 Welsh P, Polisecki E, Robertson M, *et al.* Unraveling the directional link between adiposity and inflammation: A bidirectional mendelian randomization approach. *J Clin Endocrinol Metab* 2010;**95**:93–9. doi:10.1210/jc.2009-1064

30 Klovaite J, Benn M, Nordestgaard BG. Obesity as a causal risk factor for deep venous thrombosis: A Mendelian randomization study. *J Intern Med* 2015;**277**:573–84.

For peer review only - http://bmjopen.bmj.com/site/about/guidelines.xhtml

doi:10.1111/joim.12299

31 Beun R, Kusadasi N, Sikma M, *et al.* Thromboembolic events and apparent heparin resistance in patients infected with SARS-CoV-2. *Int J Lab Hematol* Published Online First: April 2020. doi:10.1111/ijlh.13230

32 Panigada M, Bottino N, Tagliabue P, *et al.* Hypercoagulability of COVID-19 patients in Intensive Care Unit. A Report of Thromboelastography Findings and other Parameters of Hemostasis. *J Thromb Haemost* 2020;:jth.14850. doi:10.1111/jth.14850

33 Zhou F, Yu T, Du R, *et al.* Clinical course and risk factors for mortality of adult inpatients with COVID-19 in Wuhan, China: a retrospective cohort study. *Lancet* 2020;**395**:1054–62. doi:10.1016/S0140-6736(20)30566-3

34 Fry A, Littlejohns TJ, Sudlow C, *et al.* Comparison of Sociodemographic and Health-Related Characteristics of UK Biobank Participants With Those of the General Population. *Am J Epidemiol* 2017;**186**:1026–34. doi:10.1093/aje/kwx246

35 Keyes KM, Westreich D. UK Biobank, big data, and the consequences of non-representativeness. *Lancet* 2019;**393**:1297. doi:10.1016/S0140-6736(18)33067-8

For peer review only

For peer review only - http://bmjopen.bmj.com/site/about/guidelines.xhtml

Figure Legends

Figure 1. Nonlinear associations of significant continuous variables with COVID-19 and pneumonia

Figure 2. Risk factors for COVID-19 and Pneumonia in the UK Biobank cohort.

Data presented as Risk Ratios (RR) and their 95% CI. Analyses were adjusted for age, sex, ethnicity, deprivation, BMI, FEV1, and walking pace (and DBP for COVID-19 only). Continuous exposures were standardised and presented per 1-SD increment (deprivation index SD=3.01, Cystatin C SD=0.14, BMI SD=4.59, HbA1c SD=5.80, white cell count SD=1.69, FEV1 SD=0.77 and HDL cholesterol SD=0.37).

For peer review only

Table 1. Univariable association of baseline risk factors with COVID-19 in 2020, pneumonia occurring after 2016, and influenza occurring after 2016

	COVID-19				Pneumonia (after 2016)				Influenza (after 2016)			
	No n=235531	Yes n=397	RR*	P	No n=96605	Yes n=209	RR	P	No n=96720	Yes n=94	RR	P
Age (years)	66.53 (8.09)	67.55 (8.68)	1.14	0.01	66.52 (8.06)	68.78 (7.60)	1.34	< 0.0001	66.53 (8.06)	66.20 (7.83)	0.96	0.69
Age categories (years), n (%)												
<60	58683 (24.92)	102 (25.69)	1	REF	23707 (24.54)	31 (14.83)	1	REF	23717 (24.52)	21 (22.34)	1	REF
60-64	37589 (15.96)	42 (10.58)	0.64	0.02	15230 (15.77)	24 (11.48)	1.20	0.49	15233 (15.75)	21 (22.34)	1.56	0.15
65-69	42812 (18.18)	58 (14.61)	0.78	0.13	17606 (18.22)	45 (21.53)	1.95	0.004	17632 (18.23)	19 (20.21)	1.22	0.54
70-74	55726 (23.66)	93 (23.43)	0.96	0.78	23233 (24.05)	63 (30.14)	2.07	0.0009	23278 (24.07)	18 (19.15)	0.87	0.67
≥75	40721 (17.29)	102 (25.69)	1.44	0.009	16829 (17.42)	46 (22.01)	2.09	0.002	16860 (17.43)	15 (15.96)	1.00	0.99
Male sex, n (%)	111095 (47.17)	219 (55.16)	1.38	0.001	45548 (47.15)	72 (34.45)	0.59	0.0003	45573 (47.12)	47 (50.00)	1.12	0.58
Ethnicity (%)												
White	224215 (95.20)	356 (89.67)	1	REF	92477 (95.73)	201 (96.17)	1	REF	92588 (95.73)	90 (95.74)	1	REF
Black	3057 (1.30)	14 (3.53)	2.88	0.0001	908 (0.94)	3 (1.44)	1.52	0.47	911 (0.94)	0 (0.00)	0.00	0.97
South Asian	3508 (1.49)	12 (3.02)	2.15	0.009	1605 (1.66)	3 (1.44)	0.86	0.80	1607 (1.66)	1 (1.06)	0.64	0.66
Others	4751 (2.02)	15 (3.78)	1.99	0.009	1615 (1.67)	2 (0.96)	0.57	0.43	1614 (1.67)	3 (3.19)	1.91	0.27
Deprivation index (score)	-1.44 (2.98)	-0.56 (3.37)	1.30	< 0.0001	-1.48 (2.90)	-1.28 (3.07)	1.07	0.31	-1.48 (2.90)	-0.92 (3.32)	1.20	0.06
Current/former smoker, n (%)	106829 (45.36)	224 (56.42)	1.56	< 0.0001	43843 (45.38)	93 (44.50)	0.96	0.80	43891 (45.38)	45 (47.87)	1.11	0.63
Alcohol drinking status, n (%)												
Never	8894 (3.78)	27 (6.80)	0.84	0.56	3714 (3.84)	13 (6.22)	1.20	0.64	3723 (3.85)	4 (4.26)	1.20	0.80
Former	7469 (3.17)	19 (4.79)	1	REF	3090 (3.20)	13 (6.22)	1	REF	3099 (3.20)	4 (4.26)	1	REF
Current	219168 (93.05)	351 (88.41)	0.53	0.001	89801 (92.96)	183 (87.56)	0.58	0.06	89898 (92.95)	86 (91.49)	0.89	0.82
BMI (kg/m²)	27.32 (4.56)	29.12 (5.25)	1.40	< 0.0001	27.41 (4.56)	28.61 (5.58)	1.26	0.0002	27.42 (4.57)	27.53 (4.70)	1.03	0.80
BMI categories, n (%)												
Underweight	787 (0.33)	1 (0.25)	1.17	0.88	305 (0.32)	1 (0.48)	1.65	0.62	306 (0.32)	0 (0.00)	0.00	0.97
Normal	78073 (33.15)	85 (21.41)	1	REF	31182 (32.28)	62 (29.67)	1	REF	31209 (32.27)	35 (37.23)	1	REF
Overweight	101751 (43.20)	172 (43.32)	1.55	0.0009	41867 (43.34)	80 (38.28)	0.96	0.81	41917 (43.34)	30 (31.91)	0.64	0.07
Obese	54920 (23.32)	139 (35.01)	2.32	< 0.0001	23251 (24.07)	66 (31.58)	1.43	0.04	23288 (24.08)	29 (30.85)	1.11	0.68
Body fat-free mass (Kg)	53.76 (11.47)	56.18 (11.50)	1.23	< 0.0001	53.75 (11.49)	52.08 (12.04)	0.86	0.04	53.74 (11.49)	54.61 (12.17)	1.08	0.46
Body fat mass (Kg)	24.49 (9.19)	27.44 (10.35)	1.32	< 0.0001	24.69 (9.21)	27.90 (11.25)	1.35	< 0.0001	24.70 (9.22)	24.31 (9.37)	0.96	0.68
Body fat proportion (percent)	31.01 (8.40)	32.36 (8.47)	1.17	0.001	31.19 (8.41)	34.29 (8.68)	1.44	< 0.0001	31.19 (8.41)	30.51 (8.46)	0.92	0.43
Systolic blood pressure (mmHg)	137.24 (18.07)	138.49 (18.76)	1.07	0.17	137.55 (18.10)	137.55 (18.03)	1.00	1.00	137.55 (18.10)	137.60 (17.40)	1.00	0.98
Diastolic blood pressure (mmHg)	82.08 (9.90)	82.99 (10.47)	1.10	0.07	82.19 (9.90)	80.90 (9.63)	0.88	0.06	82.19 (9.90)	82.79 (10.74)	1.06	0.56
FEV1(litres)	2.86 (0.77)	2.73 (0.78)	0.84	0.0009	2.86 (0.78)	2.53 (0.81)	0.63	< 0.0001	2.86 (0.78)	2.84 (0.88)	0.98	0.84
FVC (litres)	3.79 (0.98)	3.68 (0.97)	0.89	0.03	3.79 (0.99)	3.40 (1.01)	0.65	< 0.0001	3.79 (0.99)	3.73 (1.04)	0.95	0.60
FEV1/FVC	0.76 (0.06)	0.74 (0.08)	0.85	0.0004	0.76 (0.06)	0.74 (0.08)	0.82	0.0007	0.76 (0.06)	0.76 (0.06)	1.04	0.72
Walking pace (%)												
Slow	14887 (6.32)	59 (14.86)	2.29	< 0.0001	6190 (6.41)	29 (13.88)	2.07	0.0005	6209 (6.42)	10 (10.64)	1.78	0.10
Average	123588 (52.47)	213 (53.65)	1	REF	50839 (52.63)	115 (55.02)	1	REF	50908 (52.63)	46 (48.94)	1	REF
Brisk	97056 (41.21)	125 (31.49)	0.75	0.010	39576 (40.97)	65 (31.10)	0.73	0.04	39603 (40.95)	38 (40.43)	1.06	0.78
Grip strength (Kg)	31.31 (10.93)	31.36 (10.31)	1.00	0.93	31.21 (10.96)	27.83 (10.83)	0.72	< 0.0001	31.20 (10.96)	31.94 (11.27)	1.07	0.51

For peer review only - http://bmjopen.bmj.com/site/about/guidelines.xhtml

For peer review only

Prevalent disease at baseline (%)												
Longstanding illness, n (%)	68208 (28.96)	156 (39.29)	1.59	< 0.0001	28899 (29.91)	89 (42.58)	1.74	< 0.0001	28954 (29.94)	34 (36.17)	1.33	0.19
Diabetes	10969 (4.66)	38 (9.57)	2.16	< 0.0001	4575 (4.74)	20 (9.57)	2.12	0.001	4591 (4.75)	4 (4.26)	0.89	0.82
CVD	11238 (4.77)	35 (8.82)	1.93	0.0002	4870 (5.04)	18 (8.61)	1.77	0.02	4884 (5.05)	4 (4.26)	0.84	0.73
Cancer	15849 (6.75)	32 (8.12)	1.22	0.28	6557 (6.81)	27 (12.92)	2.03	0.0006	6573 (6.82)	11 (11.70)	1.81	0.06
Depression	45781 (19.44)	68 (17.13)	0.86	0.25	18651 (19.31)	34 (16.27)	0.81	0.27	18667 (19.30)	18 (19.15)	0.99	0.97
CKD stages 3-5	293 (0.12)	2 (0.50)	4.04	0.049	128 (0.13)	0 (0.00)	0.00	0.97	128 (0.13)	0 (0.00)	0.00	0.98
SLE	274 (0.12)	0 (0.00)	0.00	0.96	125 (0.13)	0 (0.00)	0.00	0.97	125 (0.13)	0 (0.00)	0.00	0.98
Asthma	27722 (11.77)	54 (13.60)	1.18	0.26	11433 (11.83)	34 (16.27)	1.45	0.049	11454 (11.84)	13 (13.83)	1.19	0.55
Sleep apnoea	718 (0.30)	4 (1.01)	3.32	0.02	317 (0.33)	0 (0.00)	0.00	0.97	317 (0.33)	0 (0.00)	0.00	0.97
COPD	536 (0.23)	3 (0.76)	3.33	0.04	241 (0.25)	0 (0.00)	0.00	0.96	240 (0.25)	1 (1.06)	4.31	0.15
Bronchitis	2515 (1.07)	8 (2.02)	1.90	0.07	1085 (1.12)	6 (2.87)	2.59	0.02	1087 (1.12)	4 (4.26)	3.90	0.008
Pneumonia	2938 (1.25)	8 (2.02)	1.63	0.17	1152 (1.19)	4 (1.91)	1.61	0.34	1155 (1.19)	1 (1.06)	0.89	0.91
Other respiratory disease	1243 (0.53)	3 (0.76)	1.43	0.53	511 (0.53)	1 (0.48)	0.90	0.92	512 (0.53)	0 (0.00)	0.00	0.98
Medication at baseline, n (%)												
Statin	36314 (15.42)	98 (24.69)	1.80	< 0.0001	15358 (15.90)	39 (18.66)	1.21	0.28	15389 (15.91)	8 (8.51)	0.49	0.055
BP medication	37590 (15.96)	105 (26.45)	1.89	< 0.0001	15719 (16.27)	34 (16.27)	1.00	1.00	15741 (16.27)	12 (12.77)	0.75	0.36
Steroid	876 (0.37)	1 (0.25)	0.68	0.70	354 (0.37)	1 (0.48)	1.31	0.79	355 (0.37)	0 (0.00)	0.00	0.98
Biomarker at baseline												
Total cholesterol (mmol/L)	5.62 (4.92-6.34)	5.48 (4.68-6.21)	0.85	0.001	5.62 (4.91-6.34)	5.62 (4.84-6.30)	0.99	0.94	5.62 (4.91-6.34)	5.62 (5.04-6.37)	1.07	0.49
HDL cholesterol (mmol/L)	1.40 (1.17-1.67)	1.29 (1.09-1.54)	0.71	< 0.0001	1.39 (1.17-1.67)	1.41 (1.14-1.70)	1.01	0.87	1.39 (1.17-1.67)	1.36 (1.12-1.64)	0.92	0.44
Cystatin C (mg/L)	0.88 (0.80-0.97)	0.92 (0.83-1.02)	1.34	< 0.0001	0.88 (0.80-0.97)	0.89 (0.80-1.01)	1.12	0.07	0.88 (0.80-0.97)	0.91 (0.82-0.98)	1.03	0.76
HbA1c (mmol/mol)	35.10 (32.60-37.60)	36.20 (33.60-39.60)	1.30	< 0.0001	35.10 (32.60-37.60)	36.00 (33.50-39.00)	1.22	< 0.0001	35.10 (32.60-37.60)	35.10 (32.32-38.30)	1.08	0.39
CRP (mg/L)	1.25 (0.63-2.56)	1.58 (0.86-3.28)	1.15	0.0001	1.27 (0.64-2.59)	1.37 (0.84-3.27)	1.14	0.01	1.27 (0.64-2.59)	1.34 (0.70-2.93)	1.07	0.47
Rheumatoid factor (IU/ml)	3.16 (3.16-3.16)	3.16 (3.16-3.16)	1.02	0.64	3.16 (3.16-3.16)	3.16 (3.16-3.16)	1.04	0.50	3.16 (3.16-3.16)	3.16 (3.16-3.16)	1.04	0.64
Red cell distribution width (%)	13.30 (12.90-13.80)	13.32 (12.97-13.95)	1.12	0.01	13.30 (12.89-13.80)	13.38 (12.97-14.02)	1.15	0.02	13.30 (12.89-13.80)	13.27 (12.97-13.70)	0.90	0.36
White cell count (10^9 cells/L)	6.60 (5.61-7.80)	6.90 (5.84-8.26)	1.17	0.001	6.63 (5.64-7.80)	6.90 (5.80-8.29)	1.15	0.03	6.63 (5.64-7.80)	6.60 (5.50-7.76)	1.02	0.82
Neutrophil count (10^9 cells/L)	4.00 (3.25-4.90)	4.12 (3.29-5.13)	1.12	0.02	4.01 (3.26-4.92)	4.11 (3.41-5.15)	1.10	0.13	4.01 (3.26-4.92)	4.02 (3.30-5.05)	0.99	0.93
Lymphocyte count (10^9 cells/L)	1.88 (1.52-2.29)	1.90 (1.55-2.40)	1.13	0.009	1.88 (1.52-2.30)	1.92 (1.60-2.36)	1.11	0.12	1.88 (1.52-2.29)	1.85 (1.51-2.53)	1.04	0.68
Monocyte count (10^9 cells/L)	0.45 (0.36-0.56)	0.47 (0.39-0.59)	1.13	0.01	0.45 (0.37-0.57)	0.48 (0.37-0.59)	1.07	0.31	0.45 (0.37-0.57)	0.50 (0.40-0.57)	1.14	0.17

Numbers represent number (%) for categorical variables, mean (SD) for gaussian continuous variables, and median (25-75th percentiles) for skewed variables

*Univariable relative risk ratio per 1 standard deviation for continuous variables and using comparator group for categorical variables.

†Evidence for non-linearity (see supplementary material)

For peer review only - http://bmjopen.bmj.com/site/about/guidelines.xhtml

Table 2. Association of risk factors for COVID-19 and pneumonia in UK Biobank

	COVID-19				Pneumonia (after 2016)			
	Model 1		Model 2		Model 1		Model 2	
	RR (95% CI)	P	RR (95% CI)	P	RR (95% CI)	P	RR (95% CI)	P
Age (per 5 years)	1.12 (1.05, 1.19)	0.0007	1.04 (0.96, 1.11)	0.32	1.21 (1.11, 1.33)	< 0.0001	1.12 (1.01, 1.24)	0.03
Male Sex	1.36 (1.11, 1.66)	0.003	1.72 (1.25, 2.35)	0.0008	0.58 (0.44, 0.77)	0.0002	0.80 (0.56, 1.16)	0.24
Ethnicity								
White	1 (Reference)	-	1 (Reference)	-	1 (Reference)	-	1 (Reference)	-
Black	2.32 (1.33, 4.04)	0.003	2.00 (1.13, 3.53)	0.02	1.63 (0.51, 5.21)	0.41	1.20 (0.37, 3.89)	0.76
South Asian	1.98 (1.10, 3.55)	0.02	1.73 (0.94, 3.17)	0.08	0.96 (0.31, 3.05)	0.95	0.66 (0.20, 2.14)	0.49
Others	1.83 (1.08, 3.10)	0.03	1.70 (0.99, 2.91)	0.053	0.63 (0.15, 2.55)	0.51	0.52 (0.13, 2.15)	0.37
Deprivation index (per 1 SD)	1.27 (1.16, 1.39)	< 0.0001	1.17 (1.06, 1.29)	0.001	1.10 (0.96, 1.26)	0.18	1.04 (0.90, 1.20)	0.62
Current/former smoker	1.45 (1.19, 1.79)	0.0003	1.39 (1.13, 1.71)	0.002	0.95 (0.72, 1.25)	0.71	0.89 (0.67, 1.19)	0.44
Alcohol drinking status								
Never	1 (Reference)	-	-	-	1 (Reference)	-	-	-
Former	0.90 (0.49, 1.65)	0.74	-	-	1.28 (0.58, 2.79)	0.54	-	-
Current	0.65 (0.43, 1.00)	0.05	-	-	0.66 (0.37, 1.18)	0.16	-	-
BMI (per 1 SD)	1.36 (1.25, 1.48)	< 0.0001	1.28 (1.16, 1.40)	< 0.0001	1.25 (1.11, 1.41)	0.0002	1.16 (1.02, 1.32)	0.03
BMI categories								
Underweight	1.17 (0.16, 8.59)	0.88	-	-	1.43 (0.20, 10.04)	0.73	-	-
Normal	1 (Reference)	-	-	-	1 (Reference)	-	-	-
Overweight	1.43 (1.10, 1.87)	0.008	-	-	1.01 (0.72, 1.42)	0.94	-	-
Obese	2.08 (1.58, 2.74)	< 0.0001	-	-	1.44 (1.01, 2.05)	0.04	-	-
Body fat-free mass (per 1 SD)	1.39 (1.17, 1.64)	0.0002	-	-	1.42 (1.10, 1.84)	0.008	-	-
Body fat mass (per 1 SD)	1.37 (1.25, 1.50)	< 0.0001	-	-	1.27 (1.12, 1.44)	0.0002	-	-
Body fat percent (per 1 SD)	1.58 (1.37, 1.81)	< 0.0001	-	-	1.34 (1.11, 1.62)	0.002	-	-
Systolic blood pressure (per 1 SD)	1.01 (0.91, 1.12)	0.89	-	-	0.94 (0.81, 1.08)	0.39	-	-
Diastolic blood pressure (per 1 SD)	1.06 (0.96, 1.17)	0.25	-	-	0.90 (0.79, 1.04)	0.16	-	-
FEV1 (per 1 SD)	0.76 (0.66, 0.87)	< 0.0001	0.84 (0.73, 0.97)	0.02	0.70 (0.58, 0.86)	0.0005	0.75 (0.61, 0.92)	0.006
FVC (per 1 SD)	0.80 (0.70, 0.93)	0.003	-	-	0.72 (0.59, 0.90)	0.003	-	-
FEV1/FVC (per 1 SD)	0.89 (0.81, 0.98)	0.01	-	-	0.84 (0.74, 0.95)	0.007	-	-
Walking pace								
Slow	1.99 (1.48, 2.68)	< 0.0001	1.53 (1.12, 2.08)	0.007	1.91 (1.26, 2.90)	0.002	1.60 (1.04, 2.46)	0.03
Average	1 (Reference)	-	1 (Reference)	-	1 (Reference)	-	1 (Reference)	-

For peer review only - http://bmjopen.bmj.com/site/about/guidelines.xhtml

Brisk	0.80 (0.64, 1.00)	0.051	0.95 (0.75, 1.20)	0.65	0.78 (0.57, 1.06)	0.11	0.88 (0.64, 1.21)	0.42
Grip strength (per 1 SD)	0.87 (0.75, 1.01)	0.07	0.94 (0.81, 1.10)	0.45	0.85 (0.68, 1.07)	0.16	0.96 (0.76, 1.22)	0.76
Prevalent disease at baseline								
Longstanding illness	1.44 (1.17, 1.77)	0.0005	1.16 (0.93, 1.44)	0.19	1.65 (1.25, 2.18)	0.0004	1.40 (1.04, 1.88)	0.03
Diabetes	1.72 (1.22, 2.43)	0.002	1.24 (0.87, 1.77)	0.23	2.06 (1.29, 3.30)	0.002	1.59 (0.98, 2.59)	0.06
CVD	1.56 (1.09, 2.23)	0.02	1.22 (0.85, 1.76)	0.29	1.64 (1.00, 2.70)	0.052	1.35 (0.81, 2.24)	0.25
Cancer	1.22 (0.84, 1.76)	0.29	1.20 (0.83, 1.74)	0.33	1.72 (1.14, 2.60)	0.009	1.72 (1.14, 2.60)	0.01
Depression	0.96 (0.73, 1.25)	0.74	1.01 (0.77, 1.32)	0.94	0.81 (0.56, 1.18)	0.27	0.86 (0.59, 1.25)	0.42
Asthma	1.19 (0.89, 1.59)	0.23	1.04 (0.78, 1.40)	0.78	1.44 (0.99, 2.09)	0.054	1.25 (0.85, 1.82)	0.25
COPD	2.80 (0.89, 8.82)	0.08	1.81 (0.57, 5.79)	0.31	-	-	-	-
Bronchitis	1.65 (0.82, 3.36)	0.16	1.26 (0.61, 2.58)	0.53	2.23 (0.98, 5.06)	0.055	1.77 (0.77, 4.07)	0.18
Pneumonia	1.57 (0.77, 3.17)	0.21	1.50 (0.74, 3.05)	0.26	1.53 (0.57, 4.14)	0.40	1.47 (0.54, 3.99)	0.45
Other respiratory disease	1.36 (0.43, 4.26)	0.60	1.21 (0.38, 3.82)	0.74	0.84 (0.12, 6.04)	0.86	0.76 (0.10, 5.52)	0.78
Medication at baseline								
Statin	1.53 (1.20, 1.95)	0.0006	1.24 (0.97, 1.59)	0.09	1.08 (0.75, 1.55)	0.69	0.91 (0.62, 1.32)	0.62
BP medication	1.62 (1.28, 2.06)	< 0.0001	1.33 (1.04, 1.70)	0.02	0.96 (0.66, 1.41)	0.85	0.82 (0.55, 1.21)	0.31
Steroid	0.63 (0.09, 4.57)	0.65	0.53 (0.07, 3.83)	0.53	1.20 (0.17, 8.63)	0.86	1.03 (0.14, 7.53)	0.97
Biomarker								
Total cholesterol (per 1 SD)	0.89 (0.81, 0.99)	0.03	0.93 (0.85, 1.03)	0.16	0.94 (0.82, 1.08)	0.40	0.98 (0.85, 1.12)	0.76
HDL cholesterol (per 1 SD)	0.73 (0.65, 0.83)	< 0.0001	0.83 (0.73, 0.95)	0.005	0.88 (0.76, 1.03)	0.11	0.98 (0.84, 1.16)	0.84
Cystatin C (per 1 SD)	1.27 (1.16, 1.39)	< 0.0001	1.13 (1.02, 1.25)	0.02	1.08 (0.94, 1.24)	0.29	0.96 (0.83, 1.12)	0.59
HbA1c (per 1 SD)	1.23 (1.15, 1.32)	< 0.0001	1.14 (1.05, 1.23)	0.001	1.19 (1.07, 1.33)	0.001	1.11 (0.99, 1.25)	0.07
CRP (per 1 SD)	1.14 (1.06, 1.22)	0.0006	1.03 (0.95, 1.13)	0.45	1.11 (1.00, 1.24)	0.04	1.03 (0.91, 1.16)	0.67
Rheumatoid factor (per 1 SD)	1.02 (0.93, 1.12)	0.70	1.01 (0.92, 1.11)	0.82	1.03 (0.91, 1.15)	0.68	1.02 (0.90, 1.15)	0.76
Red cell distribution width (per 1 SD)	1.08 (0.99, 1.19)	0.09	1.03 (0.94, 1.13)	0.55	1.12 (0.99, 1.27)	0.07	1.07 (0.95, 1.22)	0.27
White cell count (per 1 SD)	1.15 (1.05, 1.27)	0.003	1.04 (0.94, 1.15)	0.44	1.15 (1.01, 1.32)	0.03	1.07 (0.93, 1.23)	0.32
Neutrophil count (per 1 SD)	1.12 (1.02, 1.23)	0.02	1.03 (0.93, 1.13)	0.61	1.11 (0.97, 1.27)	0.12	1.04 (0.91, 1.19)	0.57
Lymphocyte count (per 1 SD)	1.11 (1.01, 1.23)	0.02	1.03 (0.94, 1.14)	0.53	1.07 (0.94, 1.23)	0.28	1.02 (0.89, 1.17)	0.76
Monocyte count (per 1 SD)	1.09 (0.99, 1.20)	0.08	1.01 (0.92, 1.12)	0.79	1.11 (0.97, 1.28)	0.12	1.06 (0.92, 1.21)	0.43

Data presented as risk ratios and their 95% CI. continuous exposures were standardised and presented as 1-standar deviation increment. Variables with associations denoted "-" are excluded from the model due to non-significance in previous model and /or collinearity with another variable. Analyses were adjusted for Model 1 (age, sex, ethnicity and deprivation) and model 2 (model 1 plus BMI, FEV1, and walking pace (and also grip strength and DBP for COVID-19 only))

For peer review only - http://bmjopen.bmj.com/site/about/guidelines.xhtml

Table 3. Population attributable fractions of COVID-19 in the UK Biobank

	COVID-19		Pneumonia after 2016	
	**RR (95% CI)**	**PAF**	**RR (95% CI)**	**PAF**
Current/former smoker	1.39 (1.13 to 1.70)	14.94	0.91 (0.69 to 1.20)	-4.15
High deprivation index	1.45 (1.16 to 1.82)	8.33	1.00 (0.71 to 1.40)	-0.10
Male	1.18 (0.94 to 1.50)	8.00	0.64 (0.46 to 0.90)	-20.65
Age > 65 years	1.18 (0.94 to 1.46)	6.33	1.29 (0.96 to 1.73)	9.88
BMI > 30 kg/m^2	1.29 (1.03 to 1.61)	6.25	1.14 (0.83 to 1.57)	3.17
High HbA1c	1.29 (1.02 to 1.62)	5.26	1.37 (1.00 to 1.88)	6.66
Low HDL cholesterol	1.27 (1.00 to 1.61)	5.15	1.34 (0.95 to 1.91)	6.45
BP medication	1.34 (1.05 to 1.71)	5.11	0.88 (0.60 to 1.29)	-2.19
High Cystatin C	1.24 (0.98 to 1.56)	4.48	1.12 (0.80 to 1.57)	2.39
Slow walking pace	1.66 (1.23 to 2.24)	4.02	1.78 (1.17 to 2.70)	4.69
Ethnicity: Black	1.87 (1.32 to 2.65)	4.00	0.70 (0.34 to 1.45)	- 2.00
Low FEV1	1.15 (0.88 to 1.50)	2.93	1.64 (1.18 to 2.28)	11.23

Estimated using prevalence in the study sample and RR shown in this table.

All factors shown are mutually adjusted. Continuous variables were categorised with the top 20% as the at-risk group.

RR shown in this table may be overadjusted, e.g. cystatin C and HDL cholesterol may be downstream factors of BMI.

For peer review only

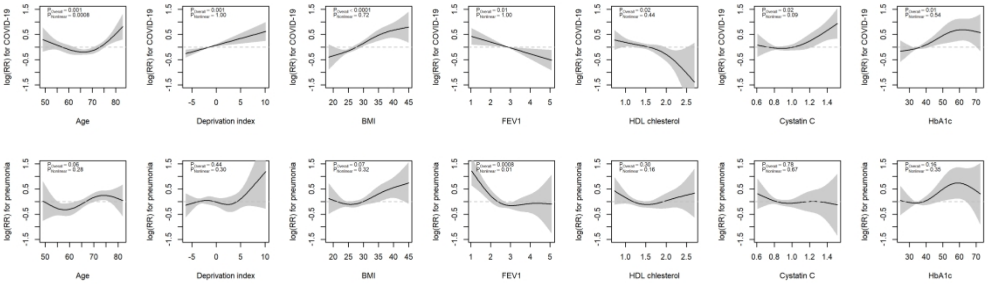

Figure 1. Nonlinear associations of significant continuous variables with COVID-19 and pneumonia

355x110mm (300 x 300 DPI)

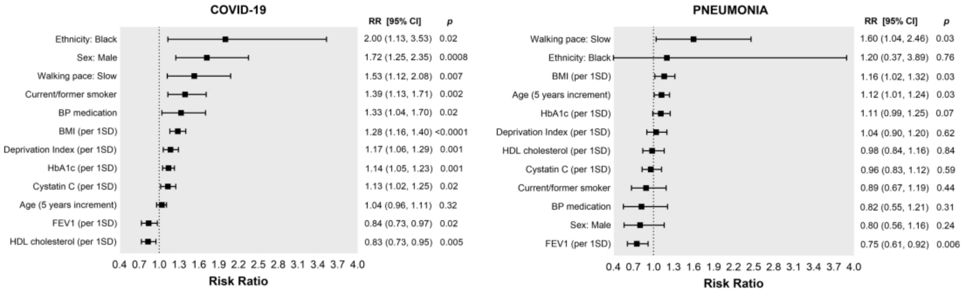

Figure 2. Risk factors for COVID-19 and Pneumonia in the UK Biobank cohort.
Data presented as Risk Ratios (RR) and their 95% CI. Analyses were adjusted for age, sex, ethnicity, deprivation, BMI, FEV1, and walking pace (and DBP for COVID-19 only). Continuous exposures were standardised and presented per 1-SD increment (deprivation index SD=3.01, Cystatin C SD=0.14, BMI SD=4.59, HbA1c SD=5.80, white cell count SD=1.69, FEV1 SD=0.77 and HDL cholesterol SD=0.37).

244x81mm (300 x 300 DPI)

Modifiable and non-modifiable risk factors for COVID-19: results from UK Biobank

Supplementary Material

Frederick K Ho*, Carlos A Celis-Morales*, Stuart R Gray, S Vittal Katikireddi, Claire L Niedzwiedz, Claire Hastie, Lyn D Ferguson, Colin Berry, Daniel F Mackay, Jason MR Gill, Jill P Pell**, Naveed Sattar MD**, Paul Welsh**

*Joint first authors

**Joint senior

For peer review only

For peer review only - http://bmjopen.bmj.com/site/about/guidelines.xhtml

SUPPLEMENTARY MATERIAL

Supplementary Table 1. Univariable association of baseline risk factors pneumonia and influenza occurring any time after baseline

	COVID-19 in all settings				Pneumonia since baseline				Influenza since baseline			
	No n=235410	Yes n=518	RR*	P	No n=94015	Yes n=2799	RR*	P	No n=95452	Yes n=1362	RR*	P
Age (years)	66.53 (8.09)	67.04 (8.93)	1.07	0.15	66.51 (8.06)	67.24 (8.18)	1.09	< 0.0001	66.54 (8.06)	65.63 (8.17)	0.90	< 0.0001
Age categories (years)												
<60	58639 (24.91)	146 (28.19)	1	REF	23107 (24.58)	631 (22.54)	1	REF	23349 (24.46)	389 (28.56)	1	REF
60-64	37569 (15.96)	62 (11.97)	0.66	0.007	14884 (15.83)	370 (13.22)	0.91	0.16	15027 (15.74)	227 (16.67)	0.91	0.25
65-69	42802 (18.18)	68 (13.13)	0.64	0.002	17128 (18.22)	523 (18.69)	1.11	0.06	17392 (18.22)	259 (19.02)	0.90	0.17
70-74	55711 (23.67)	108 (20.85)	0.78	0.049	22584 (24.02)	712 (25.44)	1.15	0.010	23000 (24.10)	296 (21.73)	0.78	0.0009
≥75	40689 (17.28)	134 (25.87)	1.32	0.02	16312 (17.35)	563 (20.11)	1.26	< 0.0001	16684 (17.48)	191 (14.02)	0.69	< 0.0001
Male sex, n (%)	111029 (47.16)	285 (55.02)	1.37	0.0004	44786 (47.64)	834 (29.80)	0.48	< 0.0001	45041 (47.19)	579 (42.51)	0.83	0.0006
Ethnicity, n (%)												
White	224116 (95.20)	455 (87.84)	4.18	< 0.0001	90008 (95.74)	2670 (95.39)	1.22	0.26	91423 (95.78)	1255 (92.14)	0.97	0.92
Black	3045 (1.29)	26 (5.02)	1	REF	879 (0.93)	32 (1.14)	1	REF	899 (0.94)	12 (0.88)	1	REF
South Asian	3502 (1.49)	18 (3.47)	2.52	0.0001	1546 (1.64)	62 (2.22)	1.34	0.02	1560 (1.63)	48 (3.52)	2.20	< 0.0001
Others	4747 (2.02)	19 (3.67)	1.97	0.004	1582 (1.68)	35 (1.25)	0.75	0.09	1570 (1.64)	47 (3.45)	2.15	< 0.0001
Deprivation index (score)	-1.44 (2.98)	-0.50 (3.39)	1.32	< 0.0001	-1.49 (2.89)	-1.19 (3.07)	1.10	< 0.0001	-1.49 (2.89)	-0.98 (3.16)	1.18	< 0.0001
Current/former smoker, n (%)	106773 (45.36)	280 (54.05)	1.42	< 0.0001	42567 (45.28)	1369 (48.91)	1.15	0.0001	43261 (45.32)	675 (49.56)	1.18	0.002
Alcohol drinking status, n (%)												
Never	8885 (3.77)	36 (6.95)	1	REF	3573 (3.80)	154 (5.50)	1	REF	3668 (3.84)	59 (4.33)	1	REF
Former	7464 (3.17)	24 (4.63)	0.79	0.38	2963 (3.15)	140 (5.00)	1.09	0.44	3045 (3.19)	58 (4.26)	1.18	0.37
Current	219061 (93.06)	458 (88.42)	0.52	0.0001	87479 (93.05)	2505 (89.50)	0.67	< 0.0001	88739 (92.97)	1245 (91.41)	0.87	0.31
BMI (kg/m^2)	27.31 (4.56)	29.15 (5.29)	1.40	< 0.0001	27.40 (4.55)	27.88 (5.01)	1.10	< 0.0001	27.41 (4.56)	27.74 (4.81)	1.07	0.009
BMI categories, n (%)												
Underweight	787 (0.33)	1 (0.19)	0.87	0.89	295 (0.31)	11 (0.39)	1.29	0.40	306 (0.32)	0 (0.00)	0.00	0.92
Normal	78044 (33.15)	114 (22.01)	1	REF	30371 (32.30)	873 (31.19)	1	REF	30821 (32.29)	423 (31.06)	1	REF
Overweight	101703 (43.20)	220 (42.47)	1.48	0.0007	40839 (43.44)	1108 (39.59)	0.95	0.21	41388 (43.36)	559 (41.04)	0.98	0.80
Obese	54876 (23.31)	183 (35.33)	2.28	< 0.0001	22510 (23.94)	807 (28.83)	1.24	< 0.0001	22937 (24.03)	380 (27.90)	1.20	0.008
Body fat-free mass (Kg)	53.76 (11.47)	56.39 (11.55)	1.25	< 0.0001	53.84 (11.50)	50.54 (10.66)	0.74	< 0.0001	53.75 (11.50)	52.98 (11.20)	0.93	0.01
Body fat mass (Kg)	24.49 (9.19)	27.48 (10.59)	1.32	< 0.0001	24.64 (9.19)	26.67 (9.89)	1.21	< 0.0001	24.69 (9.21)	25.47 (9.56)	1.08	0.002
Body fat proportion (percent)	31.01 (8.40)	32.27 (8.56)	1.16	0.0007	31.11 (8.40)	34.05 (8.28)	1.40	< 0.0001	31.18 (8.41)	32.09 (8.32)	1.11	< 0.0001
Systolic blood pressure (mmHg)	137.24 (18.07)	138.40 (18.89)	1.07	0.14	137.60 (18.11)	135.90 (17.67)	0.91	< 0.0001	137.59 (18.10)	135.00 (17.69)	0.86	< 0.0001
Diastolic blood pressure (mmHg)	82.07 (9.90)	83.13 (10.49)	1.11	0.02	82.22 (9.90)	81.22 (9.60)	0.91	< 0.0001	82.20 (9.89)	81.31 (10.16)	0.91	0.0010
FEV1 (litres)	2.86 (0.77)	2.75 (0.78)	0.86	0.0007	2.87 (0.78)	2.57 (0.73)	0.67	< 0.0001	2.86 (0.78)	2.73 (0.78)	0.84	< 0.0001
FVC (litres)	3.79 (0.98)	3.68 (0.97)	0.89	0.01	3.80 (0.99)	3.44 (0.91)	0.68	< 0.0001	3.79 (0.99)	3.63 (0.96)	0.85	< 0.0001
FEV1/FVC	0.76 (0.06)	0.75 (0.07)	0.88	0.002	0.76 (0.06)	0.75 (0.07)	0.89	< 0.0001	0.76 (0.06)	0.75 (0.07)	0.95	0.045
Walking pace, n (%)												
Slow	14872 (6.32)	74 (14.29)	2.15	< 0.0001	5895 (6.27)	324 (11.58)	1.74	< 0.0001	6098 (6.39)	121 (8.88)	1.35	0.002
Average	123516 (52.47)	285 (55.02)	1	REF	49425 (52.57)	1529 (54.63)	1	REF	50222 (52.61)	732 (53.74)	1	REF

For peer review only - http://bmjopen.bmj.com/site/about/guidelines.xhtml

Brisk	97022 (41.21)	159 (30.69)	0.71	0.0006	38695 (41.16)	946 (33.80)	0.80	< 0.0001	39132 (41.00)	509 (37.37)	0.89	0.050
Grip strength (Kg)	31.31 (10.93)	31.44 (10.39)	1.01	0.79	31.31 (10.97)	27.46 (9.99)	0.69	< 0.0001	31.22 (10.96)	29.99 (10.96)	0.89	< 0.0001
Prevalent disease at baseline, n (%)												
Longstanding illness	68156 (28.95)	208 (40.15)	1.64	< 0.0001	27777 (29.55)	1211 (43.27)	1.78	< 0.0001	28500 (29.86)	488 (35.83)	1.31	< 0.0001
Diabetes	10959 (4.66)	48 (9.27)	2.09	< 0.0001	4386 (4.67)	209 (7.47)	1.62	< 0.0001	4510 (4.72)	85 (6.24)	1.34	0.009
CVD	11229 (4.77)	44 (8.49)	1.85	< 0.0001	4689 (4.99)	199 (7.11)	1.44	< 0.0001	4805 (5.03)	83 (6.09)	1.22	0.08
Cancer	15841 (6.75)	40 (7.77)	1.16	0.36	6339 (6.76)	245 (8.80)	1.32	< 0.0001	6480 (6.81)	104 (7.66)	1.13	0.22
Depression	45765 (19.44)	84 (16.22)	0.80	0.06	18180 (19.34)	505 (18.04)	0.92	0.09	18417 (19.29)	268 (19.68)	1.02	0.72
CKD stages 3-5	291 (0.12)	4 (0.77)	6.22	0.0003	122 (0.13)	6 (0.21)	1.62	0.23	125 (0.13)	3 (0.22)	1.67	0.37
SLE	274 (0.12)	0 (0.00)	0.00	0.96	115 (0.12)	10 (0.36)	2.77	0.001	123 (0.13)	2 (0.15)	1.14	0.85
Asthma	27705 (11.77)	71 (13.71)	1.19	0.17	10897 (11.59)	570 (20.36)	1.90	< 0.0001	11250 (11.79)	217 (15.93)	1.41	< 0.0001
Sleep apnoea	718 (0.30)	4 (0.77)	2.54	0.06	301 (0.32)	16 (0.57)	1.75	0.02	311 (0.33)	6 (0.44)	1.35	0.46
COPD	535 (0.23)	4 (0.77)	3.40	0.01	220 (0.23)	21 (0.75)	3.03	< 0.0001	231 (0.24)	10 (0.73)	2.96	0.0006
Bronchitis	2514 (1.07)	9 (1.74)	1.64	0.14	1011 (1.08)	80 (2.86)	2.58	< 0.0001	1061 (1.11)	30 (2.20)	1.98	0.0002
Pneumonia	2935 (1.25)	11 (2.12)	1.72	0.08	1115 (1.19)	41 (1.46)	1.23	0.18	1140 (1.19)	16 (1.17)	0.98	0.95
Other respiratory disease	1242 (0.53)	4 (0.77)	1.47	0.45	489 (0.52)	23 (0.82)	1.56	0.03	508 (0.53)	4 (0.29)	0.55	0.23
Medication at baseline, n (%)												
Statin	36295 (15.42)	117 (22.59)	1.60	< 0.0001	14836 (15.78)	561 (20.04)	1.33	< 0.0001	15161 (15.88)	236 (17.33)	1.11	0.15
BP medication	37560 (15.96)	135 (26.06)	1.85	< 0.0001	15249 (16.22)	504 (18.01)	1.13	0.01	15504 (16.24)	249 (18.28)	1.15	0.04
Steroid	876 (0.37)	1 (0.19)	0.52	0.51	334 (0.36)	21 (0.75)	2.05	0.0009	352 (0.37)	3 (0.22)	0.60	0.37
Biomarker at baseline												
Total cholesterol (mmol/L)	5.62 (4.92-6.34)	5.45 (4.71-6.20)	0.84	< 0.0001	5.63 (4.91-6.35)	5.52 (4.80-6.26)	0.92	< 0.0001	5.63 (4.91-6.34)	5.51 (4.83-6.26)	0.92	0.0010
HDL cholesterol (mmol/L)	1.40 (1.17-1.67)	1.29 (1.09-1.53)	0.71	< 0.0001	1.39 (1.17-1.67)	1.42 (1.18-1.70)	1.05	0.004	1.40 (1.17-1.67)	1.37 (1.15-1.65)	0.93	0.01
Cystatin C (mg/L)	0.88 (0.80-0.97)	0.93 (0.83-1.02)	1.35	< 0.0001	0.88 (0.80-0.97)	0.88 (0.79-0.99)	1.05	0.01	0.88 (0.80-0.97)	0.89 (0.80-0.99)	1.08	0.004
HbA1c (mmol/mol)	35.10 (32.60-37.60)	35.90 (33.40-39.38)	0.83	< 0.0001	35.10 (32.60-37.60)	35.60 (33.10-38.50)	0.96	0.02	35.10 (32.60-37.60)	35.30 (32.80-38.20)	0.95	0.045
CRP (mg/L)	1.25 (0.63-2.56)	1.67 (0.87-3.23)	1.26	< 0.0001	1.26 (0.63-2.57)	1.54 (0.78-3.19)	1.16	< 0.0001	1.26 (0.64-2.58)	1.46 (0.71-3.04)	1.08	0.0005
Rheumatoid factor (IU/ml)	3.16 (3.16-3.16)	3.16 (3.16-3.16)	1.15	< 0.0001	3.16 (3.16-3.16)	3.16 (3.16-3.16)	1.13	< 0.0001	3.16 (3.16-3.16)	3.16 (3.16-3.16)	1.11	< 0.0001
Red cell distribution width (%)	13.30 (12.90-13.80)	13.34 (12.97-13.94)	1.03	0.50	13.30 (12.89-13.80)	13.39 (12.93-13.91)	1.04	0.03	13.30 (12.89-13.80)	13.30 (12.90-13.80)	1.04	0.07
White cell count (10^9 cells/Litre)	6.60 (5.61-7.80)	6.82 (5.82-8.20)	1.14	0.0005	6.62 (5.63-7.80)	6.89 (5.83-8.18)	1.11	< 0.0001	6.63 (5.64-7.80)	6.86 (5.75-8.07)	1.02	0.39
Neutrophil count (10^9 cells/Litre)	4.00 (3.25-4.90)	4.11 (3.30-5.11)	1.15	0.0007	4.00 (3.26-4.91)	4.20 (3.42-5.10)	1.16	< 0.0001	4.01 (3.26-4.92)	4.19 (3.32-5.07)	1.12	< 0.0001
Lymphocyte count (10^9 cells/Litre)	1.88 (1.52-2.29)	1.90 (1.55-2.40)	1.11	0.01	1.88 (1.52-2.29)	1.92 (1.55-2.39)	1.14	< 0.0001	1.88 (1.52-2.29)	1.90 (1.53-2.36)	1.10	0.0003
Monocyte count (10^9 cells/Litre)	0.45 (0.36-0.56)	0.48 (0.38-0.58)	1.11	0.01	0.45 (0.37-0.57)	0.45 (0.37-0.57)	1.10	< 0.0001	0.45 (0.37-0.57)	0.46 (0.37-0.58)	1.07	0.01

Numbers represent number (%) for categorical variables, mean (SD) for gaussian continuous variables, and median (25-75th percentiles) for skewed variables

*Univariable relative risk ratio per 1 standard deviation for continuous variables and using comparator group for categorical variables.

†Evidence for non-linearity (see supplementary table 2)

For peer review only

For peer review only - http://bmjopen.bmj.com/site/about/guidelines.xhtml

Supplementary table 2. Sensitivity analysis for pneumonia occurring after 2015.

	Pneumonia (after 2015)				Model 1		Model 2	
	No n=117181	Yes n=767	RR	P	RR (95% CI)	P	RR (95% CI)	P
Mean (SD) Age	66.52 (8.06)	68.44 (7.87)	1.28	< 0.0001	1.18 (1.12, 1.24)	< 0.0001	1.10 (1.04, 1.17)	0.001
Age categories								
<50	23642 (24.57)	96 (15.79)	1	REF	-	-	-	-
50-54	15174 (15.77)	80 (13.16)	1.30	0.09	-	-	-	-
55-59	17523 (18.21)	128 (21.05)	1.79	< 0.0001	-	-	-	-
60-64	23142 (24.05)	154 (25.33)	1.63	0.0002	-	-	-	-
≥65	16725 (17.38)	150 (24.67)	2.20	< 0.0001	-	-	-	-
Male	45416 (47.21)	204 (33.55)	0.57	< 0.0001	0.56 (0.47, 0.66)	< 0.0001	0.72 (0.58, 0.89)	0.003
Ethnicity								
White	92091 (95.72)	587 (96.55)	1	REF	1 (Reference)	-	1 (Reference)	-
Black	906 (0.94)	5 (0.82)	0.87	0.75	0.88 (0.36, 2.13)	0.78	0.72 (0.29, 1.75)	0.47
South Asian	1600 (1.66)	8 (1.32)	0.79	0.50	0.85 (0.42, 1.71)	0.65	0.66 (0.32, 1.34)	0.25
Others	1609 (1.67)	8 (1.32)	0.78	0.49	0.82 (0.41, 1.66)	0.59	0.72 (0.36, 1.45)	0.36
Mean (SD) deprivation index	-1.49 (2.90)	-1.22 (3.01)	1.09	0.03	1.12 (1.04, 1.22)	0.004	1.07 (0.98, 1.16)	0.11
Current/former smoker	43642 (45.36)	294 (48.36)	1.13	0.14	0.95 (0.72, 1.25)	0.71	1.07 (0.91, 1.26)	0.43
Alcohol drinking status								
Never	3696 (3.84)	31 (5.10)	1.01	0.98	1.28 (0.58, 2.79)	0.54	-	-
Former	3077 (3.20)	26 (4.28)	1	REF	1 (Reference)	-	-	-
Current	89433 (92.96)	551 (90.62)	0.74	0.10	0.66 (0.37, 1.18)	0.16	-	-
Mean (SD) BMI	27.41 (4.56)	28.16 (5.11)	1.16	< 0.0001	1.25 (1.11, 1.41)	0.0002	1.09 (1.01, 1.18)	0.03
BMI categories								
Underweight	304 (0.32)	2 (0.33)	1.10	0.90	1.43 (0.20, 10.4)	0.73	-	-
Normal	31058 (32.28)	186 (30.59)	1	REF	1 (Reference)	-	-	-
Overweight	41705 (43.35)	242 (39.80)	0.97	0.75	1.01 (0.72, 1.42)	0.94	-	-
Obese	23139 (24.05)	178 (29.28)	1.28	0.02	1.44 (1.01, 2.05)	0.04	-	-
Mean (SD) Body fat-free mass	53.76 (11.49)	51.26 (11.04)	0.80	< 0.0001	1.42 (1.10, 1.84)	0.008	-	-
Mean (SD) Body fat mass	24.68 (9.21)	26.95 (10.05)	1.24	< 0.0001	1.27 (1.12, 1.44)	0.0002	-	-

For peer review only - http://bmjopen.bmj.com/site/about/guidelines.xhtml

Mean (SD) Body fat percent	31.18 (8.41)	34.01 (8.33)	1.39	< 0.0001	1.34 (1.11, 1.62)	0.002	-	-
Mean (SD) Systolic blood pressure	137.56 (18.10)	137.18 (18.02)	0.98	0.61	0.94 (0.81, 1.08)	0.39	-	-
Mean (SD) Diastolic blood pressure	82.20 (9.90)	81.48 (9.76)	0.93	0.08	0.90 (0.79, 1.04)	0.16	-	-
Mean (SD) FEV1	2.86 (0.78)	2.56 (0.75)	0.66	< 0.0001	0.70 (0.58, 0.86)	0.0005	0.79 (0.70, 0.89)	< 0.0001
Mean (SD) FVC	3.79 (0.99)	3.43 (0.94)	0.67	< 0.0001	0.72 (0.59, 0.90)	0.003	-	-
Mean (SD) FEV1/FVC	0.76 (0.06)	0.74 (0.07)	0.86	< 0.0001	0.84 (0.74, 0.95)	0.007	-	-
Walking pace								
Slow	50630 (52.63)	324 (53.29)	1.90	< 0.0001	1.91 (1.26, 2.90)	0.002	1.53 (1.18, 2.00)	0.001
Average	6144 (6.39)	75 (12.34)	1	REF	1 (Reference)	-	1 (Reference)	-
Brisk	39432 (40.99)	209 (34.38)	0.83	0.03	0.78 (0.57, 1.06)	0.11	0.96 (0.80, 1.15)	0.65
Mean (SD) grip strength	31.22 (10.96)	27.84 (10.44)	0.72	< 0.0001	0.85 (0.68, 1.07)	0.16	0.96 (0.76, 1.22)	0.76
Prevalent disease at baseline								
Longstanding illness	28729 (29.86)	259 (42.60)	1.74	< 0.0001	1.65 (1.25, 2.18)	0.0004	1.40 (1.04, 1.88)	0.03
Diabetes	4549 (4.73)	46 (7.57)	1.64	0.001	2.06 (1.29, 3.30)	0.002	1.59 (0.98, 2.59)	0.06
CVD	4842 (5.03)	46 (7.57)	1.54	0.005	1.64 (1.00, 2.70)	0.052	1.35 (0.81, 2.24)	0.25
Cancer	6525 (6.80)	59 (9.80)	1.48	0.004	1.72 (1.14, 2.60)	0.009	1.72 (1.14, 2.60)	0.01
Depression	18587 (19.32)	98 (16.12)	0.80	0.047	0.81 (0.56, 1.18)	0.27	0.86 (0.59, 1.25)	0.42
Asthma	11342 (11.79)	125 (20.56)	1.93	< 0.0001	1.44 (0.99, 2.09)	0.054	1.25 (0.85, 1.82)	0.25
COPD	238 (0.25)	3 (0.49)	1.99	0.23	-	-	-	-
Bronchitis	1074 (1.12)	17 (2.80)	2.52	0.0002	2.23 (0.98, 5.06)	0.055	1.77 (0.77, 4.07)	0.18
Pneumonia	1144 (1.19)	12 (1.97)	1.67	0.08	1.53 (0.57, 4.14)	0.40	1.47 (0.54, 3.99)	0.45
Other respiratory disease	507 (0.53)	5 (0.82)	1.56	0.32	0.84 (0.12, 6.04)	0.86	0.76 (0.10, 5.52)	0.78
Medication at baseline								
Statin	15265 (15.87)	132 (21.71)	1.47	< 0.0001	1.08 (0.75, 1.55)	0.69	0.91 (0.62, 1.32)	0.62
BP medication	15636 (16.25)	117 (19.24)	1.23	0.047	0.96 (0.66, 1.41)	0.85	0.82 (0.55, 1.21)	0.31
Steroid	352 (0.37)	3 (0.49)	1.35	0.61	1.20 (0.17, 8.63)	0.86	1.03 (0.14, 7.53)	0.97
Median (IQR) Biomarker at baseline								
Total cholesterol	5.62 (4.91-6.34)	5.55 (4.80-6.26)	0.94	0.10	0.94 (0.82, 1.08)	0.40	0.98 (0.85, 1.12)	0.76
HDL cholesterol	1.39 (1.17-1.67)	1.41 (1.17-1.70)	1.05	0.20	0.88 (0.76, 1.03)	0.11	0.98 (0.84, 1.16)	0.84

For peer review only - http://bmjopen.bmj.com/site/about/guidelines.xhtml

Cystatin C	0.88 (0.80-0.97)	0.90 (0.80-1.01)	1.17	< 0.0001	1.08 (0.94, 1.24)	0.29	0.96 (0.83, 1.12)	0.59
HbA1c	35.10 (32.60-37.60)	35.70 (33.38-38.60)	1.18	< 0.0001	1.19 (1.07, 1.33)	0.001	1.11 (0.99, 1.25)	0.07
CRP	1.27 (0.64-2.59)	1.50 (0.81-3.05)	1.14	< 0.0001	1.11 (1.00, 1.24)	0.04	1.03 (0.91, 1.16)	0.67
Rheumatoid factor	3.16 (3.16-3.16)	3.16 (3.16-3.16)	1.04	0.22	1.03 (0.91, 1.15)	0.68	1.02 (0.90, 1.15)	0.76
Red cell distribution width	13.30 (12.89-13.80)	13.37 (12.95-13.91)	1.10	0.01	1.12 (0.99, 1.27)	0.07	1.07 (0.95, 1.22)	0.27
White cell count	6.63 (5.64-7.80)	6.93 (5.90-8.10)	1.15	0.0002	1.15 (1.01, 1.32)	0.03	1.07 (0.93, 1.23)	0.32
Neutrophil count	4.01 (3.26-4.92)	4.19 (3.52-5.13)	1.12	0.003	1.11 (0.97, 1.27)	0.12	1.04 (0.91, 1.19)	0.57
Lymphocyte count	1.88 (1.52-2.29)	1.92 (1.60-2.35)	1.09	0.02	1.07 (0.94, 1.23)	0.28	1.02 (0.89, 1.17)	0.76
Monocyte count	0.45 (0.37-0.57)	0.46 (0.37-0.58)	1.06	0.13	1.11 (0.97, 1.28)	0.12	1.06 (0.92, 1.21)	0.43

Variables with associations denoted "-" are excluded from the model due to non-significance in previous model and /or collinearity with another variable.

Model 1 adjusts for age, sex, ethnicity, deprivation

Model 2 adjusted for model 1 plus body fat %, FEV1, and walking pace

For peer review only

Supplementary table 3. Linearity of continuous variables with COVID-19 and influenza.

	Covid-19			Pneumonia (after 2016)			Influenza (after 2016)		
	χ^2	df	$P_{nonlinear}$	χ^2	df	$P_{nonlinear}$	χ^2	df	$P_{nonlinear}$
Age	16.32	2.00	0.0003	2.36	1.99	0.30	1.99	2.00	0.37
Deprivation index	0.01	0.07	0.15	2.63	2.00	0.27	2.01	2.00	0.37
BMI	0.85	1.98	0.65	4.93	2.00	0.08	0.00	0.03	0.08
Body fat-free mass	5.44	1.98	0.06	5.45	1.92	0.06	1.32	2.00	0.52
Body fat mass	2.74	1.99	0.25	2.30	1.99	0.31	0.96	2.00	0.62
Body fat percent	0.66	1.97	0.71	1.00	2.00	0.61	0.00	0.02	0.09
Systolic blood pressure	1.65	2.00	0.44	0.84	1.99	0.65	2.24	2.00	0.33
Diastolic blood pressure	2.55	1.99	0.28	0.14	1.51	0.86	1.60	1.97	0.44
FEV1	1.04	1.98	0.59	8.50	1.92	0.01	4.07	1.94	0.12
FVC	0.00	0.03	0.08	8.95	1.95	0.01	1.42	1.99	0.49
FEV1/FVC	3.23	1.98	0.20	3.10	1.83	0.19	0.62	2.00	0.73
Grip strength	7.05	2.00	0.03	2.75	1.93	0.24	0.37	2.00	0.83
Biomarker at baseline									
Total cholesterol	7.40	1.95	0.02	0.94	1.99	0.62	2.50	1.99	0.28
HDL cholesterol	0.00	0.03	0.08	2.69	1.94	0.25	1.89	1.98	0.38
Cystatin C	3.02	1.99	0.22	1.31	1.99	0.52	1.05	1.99	0.59
HbA1c	6.23	2.00	0.04	3.71	2.00	0.16	4.93	2.00	0.08
CRP	11.86	1.99	0.003	7.84	1.99	0.02	5.08	2.00	0.08
Rheumatoid factor	2.22	2.00	0.33	4.24	2.00	0.12	1.70	1.99	0.43
Red cell distribution width	0.55	1.99	0.76	5.07	2.00	0.08	3.64	1.95	0.16
White cell count	5.25	2.00	0.07	1.43	2.00	0.49	0.00	0.03	0.08
Neutrophil count	3.51	1.97	0.17	1.33	2.00	0.51	2.44	1.94	0.28
Lymphocyte count	2.85	1.91	0.23	1.13	2.00	0.57	13.41	2.00	0.001
Monocyte count	0.01	0.04	0.11	1.14	1.76	0.50	9.36	1.91	0.008

For peer review only

Supplementary table 4. Association of risk factors for COVID-19 and pneumonia in UK Biobank with body fat percent replacing BMI in Model 2

	COVID-19		Pneumonia (after 2016)	
	RR (95% CI)	P	RR (95% CI)	P
Age	1.02 (0.95, 1.09)	0.63	1.11 (1.00, 1.23)	0.048
Male	2.72 (1.89, 3.93)	< 0.0001	1.05 (0.67, 1.63)	0.84
Ethnicity				
White	1 (Reference)		1 (Reference)	
Black	2.01 (1.13, 3.55)	0.02	1.22 (0.38, 3.94)	0.74
South Asian	1.60 (0.87, 2.93)	0.13	0.63 (0.19, 2.03)	0.44
Others	1.67 (0.98, 2.86)	0.06	0.51 (0.12, 2.12)	0.36
Deprivation index	1.18 (1.07, 1.29)	0.0009	1.04 (0.90, 1.20)	0.57
Current/former smoker	1.38 (1.12, 1.69)	0.002	0.89 (0.67, 1.18)	0.42
Body fat percent	1.42 (1.23, 1.65)	< 0.0001	1.20 (0.98, 1.46)	0.08
FEV1	0.84 (0.73, 0.97)	0.02	0.75 (0.61, 0.92)	0.005
Walking pace				
Slow	1.61 (1.18, 2.18)	0.002	1.66 (1.08, 2.55)	0.02
Average	1 (Reference)		1 (Reference)	
Brisk	0.95 (0.75, 1.19)	0.64	0.87 (0.63, 1.20)	0.39
grip strength	0.97 (0.83, 1.13)	0.71	0.98 (0.78, 1.24)	0.88
Prevalent disease at baseline				
Longstanding illness	1.16 (0.93, 1.44)	0.18	1.40 (1.04, 1.89)	0.02
Diabetes	1.30 (0.91, 1.85)	0.15	1.65 (1.02, 2.68)	0.04
CVD	1.22 (0.84, 1.76)	0.29	1.35 (0.81, 2.25)	0.25
Cancer	1.20 (0.83, 1.74)	0.33	1.72 (1.13, 2.60)	0.01
Depression	1.01 (0.77, 1.32)	0.94	0.86 (0.59, 1.25)	0.42
Asthma	1.05 (0.78, 1.41)	0.76	1.25 (0.86, 1.83)	0.25
COPD	1.77 (0.55, 5.67)	0.34	-	-
Bronchitis	1.24 (0.60, 2.55)	0.56	1.75 (0.76, 4.03)	0.19
Pneumonia	1.50 (0.74, 3.06)	0.26	1.46 (0.54, 3.98)	0.46
Other respiratory disease	1.20 (0.38, 3.79)	0.76	0.75 (0.10, 5.48)	0.78
Medication at baseline				
Statin	1.25 (0.98, 1.61)	0.08	0.92 (0.63, 1.34)	0.67
BP medication	1.35 (1.05, 1.72)	0.02	0.83 (0.56, 1.23)	0.35
Steroid	0.52 (0.07, 3.82)	0.52	1.03 (0.14, 7.51)	0.98
Biomarker				
Total cholesterol	0.92 (0.83, 1.01)	0.09	0.97 (0.85, 1.11)	0.67
HDL cholesterol	0.82 (0.72, 0.93)	0.002	0.97 (0.83, 1.14)	0.70
Cystatin C	1.14 (1.03, 1.26)	0.009	0.97 (0.84, 1.13)	0.72
HbA1c	1.15 (1.06, 1.24)	0.0003	1.13 (1.00, 1.26)	0.04
CRP	1.04 (0.95, 1.13)	0.38	1.04 (0.92, 1.17)	0.56
Rheumatoid factor	1.01 (0.92, 1.11)	0.85	1.02 (0.90, 1.15)	0.77
Red cell distribution width	1.04 (0.95, 1.14)	0.43	1.08 (0.95, 1.23)	0.23
White cell count	1.04 (0.94, 1.15)	0.43	1.08 (0.94, 1.23)	0.29
Neutrophil count	1.03 (0.93, 1.13)	0.62	1.04 (0.91, 1.20)	0.54
Lymphocyte count	1.03 (0.94, 1.14)	0.51	1.03 (0.90, 1.17)	0.71
Monocyte count	1.01 (0.92, 1.12)	0.77	1.06 (0.92, 1.22)	0.41

Variables with associations denoted "-" are excluded from the model due to non-significance in previous model and /or collinearity with another variable. SLE and CKD also omitted due to low numbers of baseline cases.

Model 2 adjusted for age, sex, ethnicity, deprivation, body fat %, FEV1, and walking pace

For peer review only - http://bmjopen.bmj.com/site/about/guidelines.xhtml

Supplementary Figure 1. CONSORT diagram.

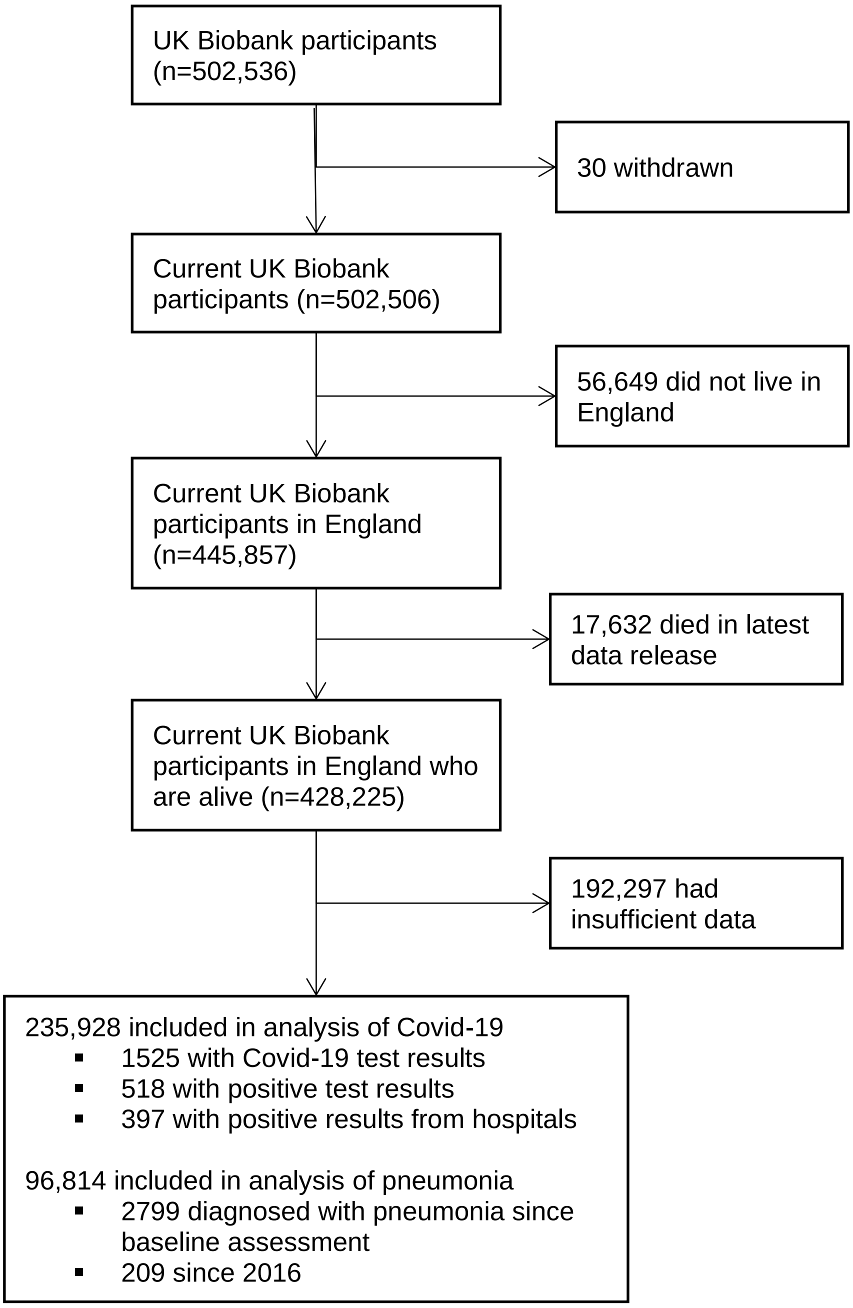

STROBE Statement—Checklist of items that should be included in reports of ***cohort studies***

	Item No	Recommendation	Page No
**Title and abstract**	1	(*a*) Indicate the study's design with a commonly used term in the title or the abstract	2
		(*b*) Provide in the abstract an informative and balanced summary of what was done and what was found	2
**Introduction**			
Background/rationale	2	Explain the scientific background and rationale for the investigation being reported	4
Objectives	3	State specific objectives, including any prespecified hypotheses	4
**Methods**			
Study design	4	Present key elements of study design early in the paper	5-6
Setting	5	Describe the setting, locations, and relevant dates, including periods of recruitment, exposure, follow-up, and data collection	5-6
Participants	6	(*a*) Give the eligibility criteria, and the sources and methods of selection of participants. Describe methods of follow-up<br>(*b*) For matched studies, give matching criteria and number of exposed and unexposed	5
Variables	7	Clearly define all outcomes, exposures, predictors, potential confounders, and effect modifiers. Give diagnostic criteria, if applicable	6-7
Data sources/ measurement	8*	For each variable of interest, give sources of data and details of methods of assessment (measurement). Describe comparability of assessment methods if there is more than one group	6-7
Bias	9	Describe any efforts to address potential sources of bias	6-7
Study size	10	Explain how the study size was arrived at	Figure s1
Quantitative variables	11	Explain how quantitative variables were handled in the analyses. If applicable, describe which groupings were chosen and why	7 Table 1
Statistical methods	12	(*a*) Describe all statistical methods, including those used to control for confounding<br>(*b*) Describe any methods used to examine subgroups and interactions<br>(*c*) Explain how missing data were addressed<br>(*d*) If applicable, explain how loss to follow-up was addressed<br>(*e*) Describe any sensitivity analyses	7-8
**Results**			
Participants	13*	(a) Report numbers of individuals at each stage of study—eg numbers potentially eligible, examined for eligibility, confirmed eligible, included in the study, completing follow-up, and analysed<br>(b) Give reasons for non-participation at each stage<br>(c) Consider use of a flow diagram	9<br>Fig s1
Descriptive data	14*	(a) Give characteristics of study participants (eg demographic, clinical, social) and information on exposures and potential confounders<br>(b) Indicate number of participants with missing data for each variable of interest<br>(c) Summarise follow-up time (eg, average and total amount)	Fig s1
Outcome data	15*	Report numbers of outcome events or summary measures over time	9

For peer review only

Main results	16	(*a*) Give unadjusted estimates and, if applicable, confounder-adjusted estimates and their precision (eg, 95% confidence interval). Make clear which confounders were adjusted for and why they were included<br>(*b*) Report category boundaries when continuous variables were categorized<br>(*c*) If relevant, consider translating estimates of relative risk into absolute risk for a meaningful time period	9-12
Other analyses	17	Report other analyses done—eg analyses of subgroups and interactions, and sensitivity analyses	Table s1-s4
**Discussion**			
Key results	18	Summarise key results with reference to study objectives	12
Limitations	19	Discuss limitations of the study, taking into account sources of potential bias or imprecision. Discuss both direction and magnitude of any potential bias	13-14
Interpretation	20	Give a cautious overall interpretation of results considering objectives, limitations, multiplicity of analyses, results from similar studies, and other relevant evidence	14
Generalisability	21	Discuss the generalisability (external validity) of the study results	13-14
**Other information**			
Funding	22	Give the source of funding and the role of the funders for the present study and, if applicable, for the original study on which the present article is based	15

*Give information separately for exposed and unexposed groups.

Note: An Explanation and Elaboration article discusses each checklist item and gives methodological background and published examples of transparent reporting. The STROBE checklist is best used in conjunction with this article (freely available on the Web sites of PLoS Medicine at http://www.plosmedicine.org/, Annals of Internal Medicine at http://www.annals.org/, and Epidemiology at http://www.epidem.com/). Information on the STROBE Initiative is available at http://www.strobe-statement.org.

For peer review only

For peer review only - http://bmjopen.bmj.com/site/about/guidelines.xhtml

BMJ Open

Modifiable and non-modifiable risk factors for COVID-19, and comparison to risk factors for influenza and pneumonia: results from a UK Biobank prospective cohort study

Journal:	*BMJ Open*
Manuscript ID	bmjopen-2020-040402.R1
Article Type:	Original research
Date Submitted by the Author:	08-Sep-2020
Complete List of Authors:	Ho, Frederick; University of Glasgow, Institute of Health and Wellbeing<br>Celis-Morales, Carlos; University of Glasgow Institute of Cardiovascular and Medical Sciences<br>Gray, Stuart; University of Glasgow, Institute of Cardiovascular and Medical Sciences, BHF Glasgow Cardiovascular Research Centre<br>Katikireddi, Srinivasa; University of Glasgow School of Life Sciences, MRC/CSO Social & Public Health Sciences Unit<br>Niedzwiedz, Claire; University of Glasgow, Institute of Health & Wellbeing<br>Hastie, Claire; University of Glasgow, Institute of Health and Wellbeing<br>Ferguson, Lyn; University of Glasgow, Institute of Cardiovascular and Medical Sciences<br>Berry, Colin; University of Glasgow, Institute of Cardiovascular and Medical Sciences, British Heart Foundation Glasgow Cardiovascular Research Centre<br>Mackay, Daniel; University of Glasgow, Public Health Unit<br>Gill, Jason; University of Glasgow, Institute of Cardiovascular and Medical Sciences<br>Pell, J. P.; University of Glasgow, Public Health Unit<br>Sattar, Naveed; University of Glasgow, Institute of Cardiovascular and Medical Sciences<br>Welsh, Paul; University of Glasgow, Institute of Cardiovascular and Medical Sciences
**Primary Subject Heading**:	Epidemiology
Secondary Subject Heading:	Infectious diseases, Diabetes and endocrinology
Keywords:	Epidemiology < INFECTIOUS DISEASES, Cardiology < INTERNAL MEDICINE, Diabetes & endocrinology < INTERNAL MEDICINE

SCHOLARONE™
Manuscripts

I, the Submitting Author has the right to grant and does grant on behalf of all authors of the Work (as defined in the below author licence), an exclusive licence and/or a non-exclusive licence for contributions from authors who are: i) UK Crown employees; ii) where BMJ has agreed a CC-BY licence shall apply, and/or iii) in accordance with the terms applicable for US Federal Government officers or employees acting as part of their official duties; on a worldwide, perpetual, irrevocable, royalty-free basis to BMJ Publishing Group Ltd ("BMJ") its licensees and where the relevant Journal is co-owned by BMJ to the co-owners of the Journal, to publish the Work in this journal and any other BMJ products and to exploit all rights, as set out in our licence.

The Submitting Author accepts and understands that any supply made under these terms is made by BMJ to the Submitting Author unless you are acting as an employee on behalf of your employer or a postgraduate student of an affiliated institution which is paying any applicable article publishing charge ("APC") for Open Access articles. Where the Submitting Author wishes to make the Work available on an Open Access basis (and intends to pay the relevant APC), the terms of reuse of such Open Access shall be governed by a Creative Commons licence – details of these licences and which Creative Commons licence will apply to this Work are set out in our licence referred to above.

Other than as permitted in any relevant BMJ Author's Self Archiving Policies, I confirm this Work has not been accepted for publication elsewhere, is not being considered for publication elsewhere and does not duplicate material already published. I confirm all authors consent to publication of this Work and authorise the granting of this licence.

For peer review only

Modifiable and non-modifiable risk factors for COVID-19, and comparison to risk factors for influenza and pneumonia: results from a UK Biobank prospective cohort study

Frederick K Ho* (0000-0001-7190-9025), Carlos A Celis-Morales* (0000-0003-2612-3917), Stuart R Gray (0000-0001-8969-9636), S Vittal Katikireddi (0000-0001-6593-9092), Claire L Niedzwiedz (0000-0001-6133-4168), Claire Hastie (0000-0002-4604-3319), Lyn D Ferguson (0000-0002-6136-8349), Colin Berry (0000-0002-4547-8636), Daniel F Mackay (0000-0001-5381-7098), Jason MR Gill (0000-0003-3615-0986), Jill P Pell** (0000-0002-8898-7035), Naveed Sattar MD** (0000-0002-1604-2593), Paul Welsh** (0000-0002-7970-3643)

*Joint first authors

**Joint senior

Affiliations

Institute of Health and Wellbeing, University of Glasgow, 1 Lilybank Gardens, Glasgow, G12 8RZ, United Kingdom Frederick K Ho research associate, S Vittal Katikireddi clinical senior research fellow, Claire L Niedzwiedz RCUK innovation fellow, Claire Hastie research associate, Daniel F Mackay professor of public health informatics, Jill P Pell director of institute

Institute of Cardiovascular and Medical Sciences, University of Glasgow, BHF Glasgow Cardiovascular Research Centre, 126 University Place, Glasgow, G12 8TA, United Kingdom Carlos A Celis-Morales research fellow, Stuart R Gray senior lecturer, Lyn D Ferguson clinical lecturer, Colin Berry professor of cardiology and imaging, Jason MR Gill professor of cardiometabolic health, Naveed Sattar professor of metabolic medicine, Paul Welsh senior lecturer

Correspondence to:

Paul Welsh

Institute of Cardiovascular and Medical Sciences, University of Glasgow, BHF Glasgow Cardiovascular Research Centre, 126 University Place, Glasgow G12 8TA, UK.

Tel: +44 (0)141 330 1722, Email address: paul.welsh@glasgow.ac.uk

ORCID ID: 0000-0002-7970-3643

For peer review only

For peer review only - http://bmjopen.bmj.com/site/about/guidelines.xhtml

Abstract

Objectives

We aimed to investigate demographic, lifestyle, socioeconomic, and clinical risk factors for COVID-19, and compared them to risk factors for pneumonia and influenza in UK Biobank.

Design

Cohort study

Setting

UK Biobank

Participants

49-83 year olds (in 2020) from a general population study

Main Outcome Measures

Confirmed COVID-19 infection (positive SARS-CoV-2 test). Incident influenza and pneumonia were obtained from primary care data. Poisson regression was used to study the association of exposure variables with outcomes.

Results

Among 235,928 participants, 397 had confirmed COVID-19. After multivariable adjustment, modifiable risk factors were higher body mass index and higher HbA1C (RR 1.28 and RR 1.14 per SD increase, respectively), smoking (RR 1.39), slow walking pace as a proxy for physical fitness (RR 1.53), , and use of blood pressure medications as a proxy for hypertension (RR 1.33). Higher forced expiratory volume in one second (FEV1) and HDL-cholesterol were both associated with lower risk (RR 0.84 and RR 0.83 per SD increase respectively). Non-modifiable risk factors included male sex (RR 1.72), black ethnicity (RR 2.00), socioeconomic deprivation (RR 1.17 per SD increase in Townsend Index), and high cystatin C (RR 1.13 per SD increase). The risk factors overlapped with pneumonia somewhat; less so for influenza. The associations with modifiable risk factors were generally stronger for COVID-19, than pneumonia or influenza.

Conclusion

These findings suggest that modification of lifestyle may help to reduce the risk of COVID-19 and could be a useful adjunct to other interventions, such as social distancing and shielding of high risk.

For peer review only

Strengths and limitations

- Large cohort from a general population, as opposed to a cohort of hospitalised patients, at an age relevant to more severe COVID-19 symptoms.
- Biochemistry assays performed in a single dedicated central laboratory.
- Extensively emerging and novel risk factors for COVID-19.
- UK Biobank is not representative of the whole UK population.
- Exposures were measured several years before the development of the outcomes, and misclassification of the exposures will likely bias our results to the null.

For peer review only

For peer review only - http://bmjopen.bmj.com/site/about/guidelines.xhtml

Introduction

COVID-19, caused by severe acute respiratory syndrome coronavirus 2 (SARS-CoV-2) infection, includes a spectrum of morbidity from asymptomatic infection[1] to severe pneumonia in patients presenting for medical care.[2] The COVID-19 pandemic[3] has led to concerted research efforts to identify people at greatest risk of developing the infection and progressing to critical illness and dying. Predictors of disease severity include older age, smoking, diabetes, hypertension, kidney disease, chronic obstructive pulmonary disease (COPD), and previous cardiovascular disease (CVD).[4–7] In addition it is becoming clear that other risk factors might include obesity and low physical fitness.[8,9] However, many studies investigate risk factors for disease progression to death or critical illness among hospitalised patients[7] as opposed to healthy comparators.

Identifying the risk factors for COVID-19 is important in terms of identifying factors that can be modified to reduce risk, as well as identifying non-modifiable risk factors that can help identify high risk groups who require shielding and targeting for testing, and eventual vaccine and anti-viral therapies. Pneumonia is a life-threatening complication of COVID-19 infection.[10] Established major risk factors for community-acquired pneumonia include many of the emerging risk factors for COVID-19.[11] As COVID-19 is caused by SARS-CoV-2 viral infection, it may have risk factors for contraction of the disease that are common to other respiratory virus conditions such as Influenza.

UK Biobank is a large prospective, deeply-phenotyped, population-based cohort study carried out in the UK.[12] Over the study period, testing for COVID-19 in England was conducted in A&E departments and in-hospital. These data were provided by Public Health England (PHE) and linked to UK Biobank baseline data. We aimed to establish modifiable and non-modifiable risk factors for confirmed COVID-19. We also aimed to compare these risk factors to risk factors for the incident pneumonia over a similar time span.

Methods

Participants

For peer review only - http://bmjopen.bmj.com/site/about/guidelines.xhtml

UK Biobank was conducted via 22 assessment centres across England, Scotland, and Wales between March 2006 and December 2010 and recruited 502,624 participants aged 37 to 73 years. The present study was restricted to participants living in England for whom COVID-19 test results were available. Death data were available to the end of January 2018 in England. To reduce competing risks, whereby risk factors may influence risk of death before the pandemic occurred, we excluded from the study all participants known to have died prior to the COVID-19 pandemic. Baseline biological measurements were recorded and touch-screen questionnaires were administered according to a standardised protocol.[12,13] UK Biobank received ethical approval from the North West Multi-Centre Research Ethics Committee (REC reference: 11/NW/03820). All participants gave written informed consent before enrolment in the study, which was conducted in accord with the principles of the Declaration of Helsinki. This study was performed under UK Biobank application number 7155.

Outcomes

Results of COVID-19 polymerase chain reaction (PCR) tests for UK Biobank participants were provided by PHE.[7,14] Data provided by PHE included the specimen date, specimen type (e.g. upper respiratory tract), laboratory, origin (whether evidence from microbiological record that the participant was an inpatient or not) and result (positive or negative). At the time the manuscript was developed,[15] data were available for the period 16th March 2020 to 3rd May 2020. Results were available for 5,356 tests conducted on 3,003 individuals. Confirmed COVID-19 infection (primary outcome) was defined as at least one positive result in the context of an in-hospital or accident and emergency (A&E) test. Any positive test result was used as a sensitivity analysis. Longer follow-up of the UK Biobank cohort has become available, but in this analysis, we have elected to investigate risk factors for more severe disease (in hospital positive tests) in the early stages of the pandemic.

Pneumonia was defined based on ICD-10 codes J12-J18, and influenza based on J09-J11, converted into Read Codes using the UK Biobank's look-up table. Incident pneumonia and influenza, occurring after 1st January 2016 (an arbitrary date taken to mimic the time lag from baseline exposure measurement to incident COVID-19 infection, while also obtaining sufficient case numbers) was obtained from a 41% sample of participants with available data from primary care. A sensitivity analysis was conducted using cases after 1st January

For peer review only - http://bmjopen.bmj.com/site/about/guidelines.xhtml

2015 and demonstrated consistent data. An analysis was also conducted exploring risk factors for all cases of pneumonia and influenza after baseline.

Exposures

Exposures were measured at the baseline assessment visit between 2006 and 2010. "Modifiable" risk factors were considered to include smoking, anthropometric measurements, glycaemic control (HbA1C), lung function measurements, hypertension, HDL-cholesterol, and measures of physical activity.

Current age (on 1st March 2020) was derived from the assessment date and age at recruitment. Ethnicity, smoking, alcohol consumption, physician-diagnosed prevalent conditions and medication use were self-reported. For the present analyses, ethnicity was coded as white, south Asian, black, or mixed/other. Smoking status was categorised into never versus former/current smoking. Systolic and diastolic blood pressures were measured at the baseline visit, preferentially using an automated measurement, but using manual measurement where this was not available, and average of available measures used. Area-level socioeconomic deprivation was assessed by the Townsend score (incorporating measures of unemployment, non-car ownership, non-home ownership and household overcrowding) corresponding to the participants' home postcode. Higher scores on the Townsend score represent greater socioeconomic deprivation. Self-reported walking pace was rated by each participant as slow, steady/average, or brisk.[16]

The definition of baseline diabetes included self-reported type 1 or type 2 diabetes, those with a primary or secondary hospital diagnoses relating to diabetes at baseline (ICD-10 codes E10-E14.9), and those who reported using diabetes medications. Baseline cardiovascular disease was defined as self-reported myocardial infarction, stroke, or transient ischaemic attack. Cancer, longstanding illness, and depression were self-reported on touchscreen questionnaire. Other previous health complaints including asthma, rheumatoid arthritis, chronic kidney disease (CKD), systemic lupus erythematosus (SLE), sleep apnoea, COPD, pneumonia, bronchitis (including bronchitis, bronchiectasis, and emphysema), and other respiratory diseases (including interstitial lung disease, asbestosis, pulmonary fibrosis, alveolitis, respiratory failure, pleurisy, pneumothorax, other respiratory

For peer review only - http://bmjopen.bmj.com/site/about/guidelines.xhtml

condition) which were derived from self-report at nurse interview. Some conditions were not included in multivariable analysis due to low case numbers and/or high correlations with other conditions, but univariable results are presented for completeness. Statin (categorised to include other cholesterol lowering medications) and blood pressure medication use were also recorded from self-report, with blood pressure medication being used as a proxy for baseline diagnosed hypertension.

Body mass index (BMI) is the ratio of the measured body mass in kg divided by height squared measured in metres. Height was measured using a Seca 202 height measure. Weight and whole-body fat mass and fat free mass were measured to the nearest 0.1 kg using the Tanita BC-418 MA body composition analyser. Socks and shoes were removed when height was measured Grip strength was measured using a Jamar J00105 hydraulic hand dynamometer and the mean was derived from the right and left hand values expressed in kilograms.[17]

Lung function was assessed by spirometry using a Vitalograph Pneumotrac 6800 spirometer (Vitalograph, Buckingham, UK). Participants did not perform spirometry if they answered yes to unsure to the following: chest infection in the last month, history of detached retina, heart attack, surgery to eyes, chest or abdomen in last 3 months, history of collapsed lung, pregnancy, or currently on medication for tuberculosis. The aim was to record two acceptable blows from a maximum of three attempts. The spirometer software compared the acceptability of the first two blows and, if acceptable (defined as a ≤5% difference in forced vital capacity (FVC) and Forced expiratory volume in one second (FEV1)), the third blow was not required. The mean observation was taken for both measures.

Blood collection sampling procedures for the study have been previously described and validated.[18] Biochemical assays were performed at a dedicated central laboratory on around 480,000 samples. Further details of these measurements can be found in the UK Biobank Data Showcase and Protocol (http://www.ukbiobank. ac.uk). For the present study we included total cholesterol, HDL cholesterol, rheumatoid factor, cystatin C, glycated haemoglobin (HbA1C), C-reactive protein (CRP), differential white blood cell count, and red cell distribution width as exposures of interest. Biomarkers with data below the limit of

For peer review only - http://bmjopen.bmj.com/site/about/guidelines.xhtml

detection were imputed as the square root of the limit of detection. The majority of participants had undetectable rheumatoid factor and risk ratios were derived for detectable rheumatoid factor, with the referent being undetectable.

Statistics

Mean and standard deviations were reported for continuous outcomes except for biomarkers, where median and interquartile ranges (IQR) were reported. Poisson regression with robust 'sandwich' standard errors were used to study the associations of exposure variables with confirmed COVID-19 and pneumonia. Poisson regressions were used because they provide risk ratio (RR) which is easier to interpret and robust error estimation ensures accurate inference.[19] Three adjustment schemes were considered: model 0 - univariate (i.e. no adjustment), model 1 – adjusted for age, sex, ethnicity, and deprivation index, and model 2 – further adjusted for behavioural (smoking and alcohol drinking) and physical (adiposity, blood pressure, spirometry, and physical capability) factors that were found to be significant in model 1. In Model 2, there were variables that were derived from the same variable (e.g. BMI and BMI categories) and have strong correlations (r=0.87 between BMI, and body fat mass, and r=0.97 between FEV1 and FVC). To avoid multicollinearity, in model 2, we chose one from, BMI, BMI categories, body fat-free mass, body fat mass, and body fat percent, and one from FEV1, FVC, and FEV1/FVC. For continuous variables, the linearity of exposure-outcome associations were tested using penalised cubic splines in generalised additive model (GAM).[20] Nonlinearity was tested using likelihood ratio test comparing a model with the exposure fitted on a spline with a model assuming a linear exposure-outcome relationship. P-value for nonlinearity < 0.05 suggest evidence against the linearity assumption. Spline smoothness was chosen using generalised cross validation.[21] Population attributable fractions (PAFs) were calculated to determine the relative contribution of each risk factor to the overall number of confirmed COVID-19 cases within UK Biobank. Another Poisson model which included all significant factors in model 2 was fitted to estimate the mutually adjusted relative risks (RR)s. These RRs were biased towards null because of over adjustment bias but were used to construct the PAFs to ensure the PAF estimates did not overlap or exceed 100%. In general, two-tailed P-values < 0.05 were considered statistically significant. Analyses were conducted in R Statistical Software version 3.5.3 with the package "mgcv".

For peer review only - http://bmjopen.bmj.com/site/about/guidelines.xhtml

Patient and public involvement

UK Biobank maintains a website and twitter feed to keep participants, the general public, and researchers up to date on the study (http://www.ukbiobank.ac.uk/news/). There is an annual scientific meeting which is recorded and available to the public as webcast. The results of the present study are shared through these channels as the UK Biobank organisation deem appropriate, our own twitter feeds, and open access publication.

The study was set up by the MRC, Department of Health and Wellcome Trust with input from major patient representative organisations (British Heart Foundation and Cancer Research UK: http://www.ukbiobank.ac.uk/public-consultation/).

Results

Of 445,857 participants in England, 428,225 were alive during the available follow-up period. Complete data on covariates were available for 235,928(55.1%) participants. Primary care data on incident pneumonia were available in 96,814 (41.0%) participants (Supplementary Fig 1). At 1st March 2020, the age range of eligible participants was 49-83 years, and time elapsed from baseline was median 10.97 years (IQR 10.36-11.55 years). Of these participants, 1,525 received at least one COVID-19 test, and 518 had confirmed SARS-CoV-2 infection, with 397 positive results conducted in hospital or A&E (primary outcome).

Univariable risk factors for incident COVID-19

Key univariable potentially modifiable risk factors for confirmed COVID-19 included current and former smoking (RR 1.56), higher BMI, body fat, and HbA1C (RR 2.32 for obesity), poor lung function (RR 0.84 for 1 SD increase in FEV1), treated hypertension (RR 1.89), HDL-cholesterol (RR 0.71 per SD increase) and slow walking pace (RR 2.29) (Table 1). Among non-modifiable risk factors were older age (particularly the over 75-year olds; RR 1.44), male sex (RR 1.38), black ethnicity (RR 2.88), socioeconomic deprivation (RR 1.30 per SD increase in Townsend Index) (Table 1). Among baseline comorbidities, general long standing illness (RR 1.59), baseline diabetes (RR 2.16), baseline CVD (RR 1.93), sleep apnoea (RR 3.32), statin use (RR 1.80), higher inflammatory markers (particularly white blood cell count; RR 1.17 per SD

For peer review only - http://bmjopen.bmj.com/site/about/guidelines.xhtml

increase), and higher cystatin C (RR 1.34 per SD increase) were also associated with confirmed COVID-19 (Table 1). Risk factor associations were similar when all 518 test positive COVID-19 cases were considered, rather than only in-hospital positive tests (supplementary table 1)

Univariable risk factors for incident pneumonia

Of the 96,814 participants with primary care data, 209 had pneumonia recorded after 2016.

Of the modifiable risk factors, pneumonia was associated with BMI, body fat and HbA1C (more moderately than COVID-19; RR 1.43 for obesity), poor lung function (more strongly than COVID-19; RR 0.63 for 1 SD increase in FEV1), and slow walking pace (RR 2.07), but not smoking, lipids, blood pressure, or blood pressure medication use. Among non-modifiable risk factors, incident pneumonia was more common in participants who were older (RR 2.09 in the over 75 year olds), and in women (RR 0.59 among men) (Table 1). Ethnicity was not associated with pneumonia. Among comorbidities, baseline diabetes, CVD and cancer were approximately twice as common in those who developed pneumonia (Table 1), and any longstanding illness was also associated with pneumonia. Inflammatory markers were similarly associated with pneumonia as with COVID-19.

When investigating risk factors for pneumonia over the full follow-up time from baseline, risk factors were generally similar although there was increased power due to a higher number of incident cases (Supplementary table 1). In this analysis smoking was associated with pneumonia (RR 1.15) as was blood pressure medication use (RR 1.13). Similar findings were found when we studied pneumonia occurring after 2015 (Supplementary Table 2).

Univariable risk factors for incident influenza

Of the 96,814 participants with primary care data, 94 had influenza recorded after 2016.

Those who developed influenza were generally slightly more socioeconomically deprived (RR 1.20 per SD increase in Townsend score), more likely to have bronchitis at baseline, and less likely to take statins. No other risk factors showed associations (Table 1). When all influenza cases occurring after baseline were considered, evidence of statistically significant

For peer review only - http://bmjopen.bmj.com/site/about/guidelines.xhtml

associations emerged due to increased power. Smoking (RR 1.18), poor lung function (RR 0.84 for 1 SD increase in FEV), slow walking pace (RR1.35), and high BMI (RR 1.20 for obesity) were all associated with higher influenza risk (Supplementary Table 1). Cases less common in older people (RR 0.69 in the over 75 year olds) and more common in women (RR 0.83 in men). Black people (RR 2.20) and South Asians (RR 2.15) were at increased risk.

Multivariable models for COVID-19 and pneumonia

After multivariable adjustment for age, sex, ethnicity, and socioeconomic status, the modifiable risk factors for confirmed COVID-19 included smoking (RR 1.45 (95% CI 1.19-1.79)), higher BMI (RR 1.36 per SD increase (95% CI 1.25-1.48)) and other measures of body fat, higher HbA1C (RR 1.23 per 1 SD increase (95% CI 1.15-1.32)), as well as blood pressure medication, FEV1 and FVC , and slow walking pace (RR 1.99 (95% CI 1.48-2.68)) (Model 1, Table 2). Other risk factors were older age (RR 1.12 per 5 years (95% CI 1.05-1.19)), male sex (RR 1.36 (95% CI 1.11-1.66)), black ethnicity (RR 2.32 (95% CI 1.33, 4.04)), South Asian ethnicity (RR 1.98 (95%CI 1.10-3.55)) socioeconomic deprivation (RR 1.27 per SD increase in Townsend Index (95% CI 1.16-1.39)). Among comorbidities, risk factors included longstanding illness (RR 1.44 (95% CI 1.17-1.77)), diabetes, CVD and statin use. Among blood biomarkers, risk factors included lower total and HDL-cholesterol, higher cystatin C (RR 1.27 per 1 SD increase (95% CI 1.16-1.39)), CRP and white blood cell count (Table 2).

After adding BMI, blood pressure, FEV1 and walking pace as mediators/covariates to the adjustment model, modifiable risk factors for COVID-19 admission continued to include smoking (RR 1.39 (95% CI 1.13-1.73)), higher BMI (RR 1.28 (95% CI 1.16-1.40)), higher HbA1C (RR 1.14 (956%CI 1.05,1.23), treated hypertension (RR 1.33 95% CI 1.04-1.70) and slow walking pace (RR 1.53 (95% CI 1.12-2.08)). The age association was no longer significant after these additional adjustments. Other risk factors included male sex (RR 1.72 (95% CI 1.25-2.35)), black ethnicity (RR 2.00 (95% CI 1.16, 3.53)), socioeconomic deprivation (RR 1.17 (95% CI 1.06-1.29)), lower HDL-cholesterol (RR 0.83 (95% CI 0.73-0.95)), and higher cystatin C (RR 1.13 (95% CI 1.02-1.25)) (Model 2, Table 2).

Nonlinear associations of continuous variables with COVID-19 and pneumonia are shown in Figure 1 (and supplementary table 3). Age was associated with COVID-19 admission in a J-

For peer review only - http://bmjopen.bmj.com/site/about/guidelines.xhtml
For peer review only

shaped curve, where for participants aged 60-70 years the curve was relatively flat, and for participants aged over 75 years risk increased exponentially with age. The associations of BMI and FEV1 with COVID-19 were fairly linear. Associations were similar when adjusting for body fat percentage rather than BMI (Supplementary Table 4).

Directly contrasting model 2 for COVID-19 and pneumonia (Figure 2), the risk factors in common for both conditions were higher BMI and slow walking pace, although less strongly associated with pneumonia than for COVID-19. Highlighting specific differences, smoking and treated blood pressure were only associated with COVID-19. Pneumonia was more common in women, and socioeconomic deprivation, HDL cholesterol, cystatin-C, and HbA1c were not associated with pneumonia. Low FEV1 was more strongly associated with pneumonia than with COVID-19. Pneumonia also showed an association with baseline cancer.

Population attributable risks

Factors that were significant in Table 2 were mutually adjusted to compare their population attributable fractions for COVID-19 and pneumonia (Table 3). Among potentially modifiable risk factors smoking accounted for 14.9% of COVID-19 cases that occurred within the UK Biobank population, obesity with 6.3%, high HbA1C with 5.3%, treated BP with 5.1%, and slow walking pace with 4.0% (total 35.6%). In contrast, none of these factors were large contributors to pneumonia cases within UK Biobank. (Table 3)

Discussion

In this population-based study, we found that confirmed COVID-19 infection was associated with a number of modifiable risk factors, a trend that less apparent for pneumonia and influenza. In particular, the associations of smoking, BMI (and body fat), hypertension, and physical fitness (as measured by slow walking pace) with COVID-19 are of note; even when such factors were measured a decade before infection they potentially accounted for one-third of COVID-19 cases in UK Biobank. The associations of FEV1 and BMI with COVID-19 are linear, suggesting even modest improvements in lifestyle may be beneficial to risk of

For peer review only - http://bmjopen.bmj.com/site/about/guidelines.xhtml

presumed severe COVID symptoms. The other independent risk factors for COVID-19 infection included older age, male sex, black ethnicity, socioeconomic deprivation, longstanding illness, and reduced renal function as measured by cystatin C, the latter also notable given renal complication in severe COVID-19. Of note, the modifiable risk factors explained some of the association of age with COVID-19 in our adjusted model.

Since this analysis was conducted[15] several other studies have also shown the association between modifiable and non-modifiable risk factors and COVID-19 risk,[22–29] particularly with obesity. Generally, these data support our analyses showing the importance of both modifiable and non-modifiable risk factors. It is important to recognise the competing risk for health of lockdown and social distancing. Social distancing reduces viral transmission, but also has consequences for lifestyle. Previous data suggests that physical fitness can be rapidly lost when activity levels decrease,[30,31] and this will also result in an increase in BMI.[32,33] Further, social distancing may increase loneliness, depression, and psychological stress in some people and consequently adversely affect eating habits[34] and other health behaviours. Anecdotal evidence from our clinics and media suggest many are struggling with overeating. This study suggests public health guidance should focus on reducing the risk of severe complications of COVID-19 by advocating a healthy lifestyle during the ongoing pandemic, not just for general cardiovascular and metabolic health, but also to help to protect against COVID-19 infection, used alongside other public health interventions.

Understanding the actual causes of disease, rather than markers that simply correlate with exposures, is clearly a key issue to consider. The independent association of socioeconomic deprivation with COVID-19 after multiple adjustment may be explained by the accumulation of earlier life socioeconomic adversities which can result in less physiological reserve and more multimorbidity.[34,35] It may also be linked to more overcrowding, reduced social distancing, and potential exposure to greater viral load. Asthma, diabetes and high blood pressure, which have been shown to be associated with a higher risk of severe COVID-19 outcomes[36] also showed some trends to be associated with COVID-19 in this dataset. While substantial focus of COVID-19 research has been its apparently more aggressive symptoms and disease progression in older people, it is important to recognise that people

For peer review only - http://bmjopen.bmj.com/site/about/guidelines.xhtml

who are older have less cardiorespiratory reserve to cope with COVID-19 infection. Older age is also associated with more hypertension and diabetes, poorer lung function, and greater relative fat mass.[34]

Previous reports have suggested that obesity or excess ectopic fat deposition may be a unifying risk factor for severe Covid-19 infection,[8,37] reducing both protective cardiorespiratory reserve as well as potentiating the immune dysregulation that appears, at least in part, to mediate the progression to critical illness in a proportion of COVID-19 patients. Our analysis bears out these hypotheses. Once BMI or body fat was adjusted for, inflammatory markers were no longer associated with incident COVID-19. This suggests proinflammatory markers, arising from increased adipose deposition, are probably acting as a marker for body fat which seems to be an adverse risk factor for severe COVID-19.[8,37–39] Furthermore, obesity enhances thrombosis,[40] which is relevant given the association between severe COVID-19 and pro-thrombotic disseminated intravascular coagulation and high rates of venous thromboembolism,[41,42] as well as the association with D-dimer seen in other reports.[43] D-dimer was not measured in blood samples in UK Biobank.

Given that pneumonia is a critical clinical complication of COVID-19, it is important to understand how risk factors for COVID-19 related pneumonia differ from "classic" community acquired pneumonia.[11] Our comparison between other common respiratory diseases and COVID-19 suggests that identification of at-risk groups based on our understanding of other respiratory diseases may be inadequate – a more refined approach to risk stratification based on COVID-19 specific risks is needed, and future data should focus on some of the exposures we identify to achieve this.

The strengths of our study include the large cohort size at an age relevant to more severe COVID-19 symptoms, and biochemistry assays performed in a single dedicated central laboratory. We were also able to extensively explore emerging and novel risk factors for COVID-19, while simultaneously comparing to risk factors for pneumonia identified to primary care providers. Limitations include that UK Biobank is not representative of the whole UK population,[44] (our data focuses on England specifically) although this is generally not a concern in investigating risk associations.[45] Care should be taken in

For peer review only - http://bmjopen.bmj.com/site/about/guidelines.xhtml

generalising the PAF estimates. These related to cases occurring within the UK Biobank population, but are not directly applicable to the general population where the prevalence of risk factors is different. In addition, mutual adjustment for overlapping risk factors can lead to problems in interpretability of the PAFs.[46] However, the direction of causal associations with this new outcome is not clear, and these exploratory analyses allow a direct comparison between COVID-19 and pneumonia in the same cohort. Due to the under-representation of non-white ethnicities in UK Biobank, we have limited power to explore important interactions by ethnic group (although Black ethnicity was associated with the outcome), and we recognise this as an important risk factor. Ascertainment bias, including differential healthcare seeking, differential testing and differential prognosis may explain some differences in outcomes given poor coverage of testing in the UK. It is also likely that cases will generally be at the more severe end of the clinical spectrum by using hospitalised cases as the outcome, although use of admission to ITU would be additionally informative once sufficient case numbers accrue. Despite this, we still observe many similar risk factors to incident pneumonia, which is more likely to have close to complete case ascertainment in primary care records, although, as discussed, other risk factors seem more strongly linked to COVID-19 infections. Exposures were measured several years before the development of the outcomes, and misclassification of the exposures will likely bias our results to the null. However, this also serves to illustrate that the risk factors were generally present many years before development of the disease, and as is well known, risk factors tend to track with age. We have also been unable to fully exclude all deaths that occurred prior to the pandemic, due to lack of up-to-date linkage to mortality records.

In conclusion, these data from UK Biobank suggest risk factors for confirmed COVID-19 infection differ in some important ways from risk factors for pneumonia, being more common in males than females, in lower SES, and with stronger associations with ethnicity, CV risk markers, prior smoking and adiposity. Such findings suggest possible merit in advocating improvements in lifestyle as an additional measure to reduce the risk of COVID-19 alongside existing public health measures such as social distancing and shielding of high risk groups. They also have implications for health advice targeted at the public to lessen risks during this pandemic.

For peer review only - http://bmjopen.bmj.com/site/about/guidelines.xhtml

Contributors

PW, JPP and NS conceived the idea for the paper. FH conducted the analysis. PW, FH, CCM and NS jointly wrote the first draft. SRG, SVK, CLN, CH, LDF, CB, DFM, JMRG, and JPP contributed to the interpretation of the findings, critically revised the paper for intellectual content, and gave approved the final version of the manuscript. PW and NS are guarantors of the work. The corresponding author attests that all listed authors meet authorship criteria and that no others meeting the criteria have been omitted.

Transparency Statement

The guarantors affirm that the manuscript is an honest, accurate, and transparent account of the study being reported; that no important aspects of the study have been omitted; and that any discrepancies from the study as originally planned (and, if relevant, registered) have been explained.

Funding

The work in this study is supported by the British Heart Foundation Centre of Research Excellence Grant RE/18/6/34217. CLN acknowledges funding from a Medical Research Council Fellowship (MR/R024774/1). SVK acknowledges funding from the Medical Research Council (MC_UU_12017/13), Scottish Government Chief Scientist Office (SPHSU13), and NRS Senior Clinical Fellowship (SCAF/15/02). The funders played no part in the research.

Competing interests: All authors have completed the ICMJE uniform disclosure form at www.icmje.org/coi_disclosure.pdf and declare: support from funders as acknowledged above for the submitted work; PW has received research grants from Roche Diagnostics, AstraZeneca and Boehringer Ingelheim outside the submitted work, and NS has received grant and personal fees from Boehringer Ingelheim, and personal fees from Amgen, AstraZeneca, Eli Lilly, Novo Nordisk, Pfizer, and Sanofi outside the submitted work; all authors declare no other relationships or activities that could appear to have influenced the submitted work.

Ethics Approval

For peer review only - http://bmjopen.bmj.com/site/about/guidelines.xhtml

UK Biobank received ethical approval from the North West Multi-centre Research Ethics Committee (REC reference: 11/NW/03820). All participants gave written informed consent before enrolment in the study, which was conducted in accordance with the principles of the Declaration of Helsinki. Direct dissemination of the results to participants is not possible/applicable.

Data Sharing

UK Biobank data can be requested by bona fide researchers for approved projects, including replication, through https://www.ukbiobank.ac.uk/.

Copyright

I, the Submitting Author has the right to grant and does grant on behalf of all authors of the Work (as defined in the below author licence), an exclusive licence and/or a non-exclusive licence for contributions from authors who are: i) UK Crown employees; ii) where BMJ has agreed a CC-BY licence shall apply, and/or iii) in accordance with the terms applicable for US Federal Government officers or employees acting as part of their official duties; on a worldwide, perpetual, irrevocable, royalty-free basis to BMJ Publishing Group Ltd ("BMJ") its licensees and where the relevant Journal is co-owned by BMJ to the co-owners of the Journal, to publish the Work in BMJ Open and any other BMJ products and to exploit all rights, as set out in our licence.

For peer review only

References

1 Mizumoto K, Kagaya K, Zarebski A, *et al.* Estimating the asymptomatic proportion of coronavirus disease 2019 (COVID-19) cases on board the Diamond Princess cruise ship, Yokohama, Japan, 2020. Eurosurveillance. 2020;**25**. doi:10.2807/1560-7917.ES.2020.25.10.2000180

2 Wu Z, McGoogan JM. Characteristics of and Important Lessons from the Coronavirus Disease 2019 (COVID-19) Outbreak in China: Summary of a Report of 72314 Cases from the Chinese Center for Disease Control and Prevention. *JAMA - J Am Med Assoc* 2020;**323**:1239–42. doi:10.1001/jama.2020.2648

3 Coronavirus disease 2019 (COVID-19) Situation Report-76 HIGHLIGHTS.

4 Petrilli CM, Jones SA, Yang J, *et al.* Factors associated with hospitalization and critical illness among 4,103 patients with COVID-19 disease in New York City. *medRxiv* 2020;:2020.04.08.20057794. doi:10.1101/2020.04.08.20057794

5 Tu H, Tu S, Gao S, *et al.* The epidemiological and clinical features of COVID-19 and lessons from this global infectious public health event. *J Infect* 2020;**0**. doi:10.1016/j.jinf.2020.04.011

6 Banerjee A, Pasea L, Harris S, *et al.* Estimating excess 1- year mortality from COVID-19 according to underlying conditions and age in England: a rapid analysis using NHS health records in 3.8 million adults. *medRxiv* 2020;:2020.03.22.20040287. doi:10.1101/2020.03.22.20040287

7 Richardson S, Hirsch JS, Narasimhan M, *et al.* Presenting Characteristics, Comorbidities, and Outcomes Among 5700 Patients Hospitalized With COVID-19 in the New York City Area. *JAMA* Published Online First: April 2020. doi:10.1001/jama.2020.6775

8 Simonnet A, Chetboun M, Poissy J, *et al.* High prevalence of obesity in severe acute respiratory syndrome coronavirus-2 (SARS-CoV-2) requiring invasive mechanical ventilation. *Obesity (Silver Spring)* Published Online First: April 2020. doi:10.1002/oby.22831

9 Obesity in patients younger than 60 years is a risk factor for Covid-19 hospital admission | Clinical Infectious Diseases | Oxford Academic.

10 2 Treatment and care planning | COVID-19 rapid guideline: managing suspected or confirmed pneumonia in adults in the community | Guidance | NICE.

11 Almirall J, Serra-Prat M, Bolíbar I, *et al.* Risk Factors for Community-Acquired Pneumonia in Adults: A Systematic Review of Observational Studies. *Respiration* 2017;**94**:299–311. doi:10.1159/000479089

12 Collins R. What makes UK Biobank special? *Lancet* 2012;**379**:1173–4. doi:10.1016/S0140-6736(12)60404-8

13 Sudlow C, Gallacher J, Allen N, *et al.* UK Biobank: An Open Access Resource for Identifying the Causes of a Wide Range of Complex Diseases of Middle and Old Age. *PLoS Med* 2015;**12**:e1001779. doi:10.1371/journal.pmed.1001779

14 Armstrong J, Rudkin JK, Allen N, *et al.* Dynamic linkage of covid-19 test results between public health england’s second generation surveillance system and uk biobank. *Microb Genomics* 2020;**6**:1–9. doi:10.1099/mgen.0.000397

15 Ho FK, Celis-Morales CA, Gray SR, *et al.* Modifiable and non-modifiable risk factors for COVID-19: results from UK Biobank. *medRxiv* 2020;:2020.04.28.20083295. doi:10.1101/2020.04.28.20083295

For peer review only - http://bmjopen.bmj.com/site/about/guidelines.xhtml

16 Celis-Morales CA, Gray S, Petermann F, *et al.* Walking Pace Is Associated with Lower Risk of All-Cause and Cause-Specific Mortality. *Med Sci Sports Exerc* 2019;**51**:472–80. doi:10.1249/MSS.0000000000001795

17 Celis-Morales CA, Welsh P, Lyall DM, *et al.* Associations of grip strength with cardiovascular, respiratory, and cancer outcomes and all cause mortality: Prospective cohort study of half a million UK Biobank participants. *BMJ* 2018;**361**:k1651. doi:10.1136/bmj.k1651

18 Elliott P, Peakman TC. The UK Biobank sample handling and storage protocol for the collection, processing and archiving of human blood and urine. *Int J Epidemiol* 2008;**37**:234–44. doi:10.1093/ije/dym276

19 Zou G. A modified poisson regression approach to prospective studies with binary data. *Am J Epidemiol* 2004;**159**:702–6. doi:10.1093/aje/kwh090

20 Wood SN. Stable and efficient multiple smoothing parameter estimation for generalized additive models. *J Am Stat Assoc* 2004;**99**:673–86. doi:10.1198/016214504000000980

21 Wood SN, Pya N, Säfken B. Smoothing Parameter and Model Selection for General Smooth Models. *J Am Stat Assoc* 2016;**111**:1548–63. doi:10.1080/01621459.2016.1180986

22 Williamson EJ, Walker AJ, Bhaskaran K, *et al.* Factors associated with COVID-19-related death using OpenSAFELY. *Nature* 2020;**584**:430–6. doi:10.1038/s41586-020-2521-4

23 Zhu Z, Hasegawa K, Ma B, *et al.* Obesity & genetic predisposition with COVID-19. *Metabolism* 2020;:154345. doi:10.1016/j.metabol.2020.154345

24 McQueenie R, Foster HME, Jani BD, *et al.* Multimorbidity, polypharmacy, and COVID-19 infection within the UK Biobank cohort. *PLoS One* 2020;**15**:e0238091. doi:10.1371/journal.pone.0238091

25 Woolford SJ, D'Angelo S, Curtis EM, *et al.* COVID-19 and associations with frailty and multimorbidity: a prospective analysis of UK Biobank participants. *Aging Clin Exp Res* Published Online First: 2020. doi:10.1007/s40520-020-01653-6

26 Sattar N, Ho FK, Gill JM, *et al.* BMI and future risk for COVID-19 infection and death across sex, age and ethnicity: Preliminary findings from UK biobank. *Diabetes Metab Syndr Clin Res Rev* 2020;**14**:1149–51. doi:10.1016/j.dsx.2020.06.060

27 Razieh C, Zaccardi F, Davies MJ, *et al.* Body mass index and the risk of COVID-19 across ethnic groups: Analysis of UK Biobank. Diabetes, Obes. Metab. 2020. doi:10.1111/dom.14125

28 Batty GD, Deary IJ, Luciano M, *et al.* Psychosocial factors and hospitalisations for COVID-19: Prospective cohort study based on a community sample. *Brain Behav Immun* Published Online First: 2020. doi:10.1016/j.bbi.2020.06.021

29 Hamer M, Gale CR, Kivimäki M, *et al.* Overweight, obesity, and risk of hospitalization for COVID-19: A community-based cohort study of adults in the United Kingdom. *Proc Natl Acad Sci* 2020;:202011086. doi:10.1073/pnas.2011086117

30 Olsen RH, Krogh-Madsen R, Thomsen C, *et al.* Metabolic responses to reduced daily steps in healthy nonexercising men. JAMA - J. Am. Med. Assoc. 2008;**299**:1261–3. doi:10.1001/jama.299.11.1259

31 Drummond MJ, Dickinson JM, Fry CS, *et al.* Bed rest impairs skeletal muscle amino acid transporter expression, mTORC1 signaling, and protein synthesis in response to essential amino acids in older adults. *Am J Physiol - Endocrinol Metab* 2012;**302**. doi:10.1152/ajpendo.00603.2011

For peer review only - http://bmjopen.bmj.com/site/about/guidelines.xhtml

32 Bradbury KE, Guo W, Cairns BJ, *et al.* Association between physical activity and body fat percentage, with adjustment for BMI: A large cross-sectional analysis of UK Biobank. *BMJ Open* 2017;**7**:e011843. doi:10.1136/bmjopen-2016-011843

33 Parsons TJ, Manor O, Power C. Physical activity and change in body mass index from adolescence to mid-adulthood in the 1958 British cohort. *Int J Epidemiol* 2006;**35**:197–204. doi:10.1093/ije/dyi291

34 Razzoli M, Pearson C, Crow S, *et al.* Stress, overeating, and obesity: Insights from human studies and preclinical models. Neurosci. Biobehav. Rev. 2017;**76**:154–62. doi:10.1016/j.neubiorev.2017.01.026

35 Packard CJ, Bezlyak V, McLean JS, *et al.* Early life socioeconomic adversity is associated in adult life with chronic inflammation, carotid atherosclerosis, poorer lung function and decreased cognitive performance: A cross-sectional, population-based study. *BMC Public Health* 2011;**11**:42. doi:10.1186/1471-2458-11-42

36 Zhao X, Zhang B, Li P, *et al. Incidence, clinical characteristics and prognostic factor of patients with COVID-19: a systematic review and meta-analysis*. Cold Spring Harbor Laboratory Press 2020. doi:10.1101/2020.03.17.20037572

37 Lighter J, Phillips M, Hochman S, *et al.* Obesity in patients younger than 60 years is a risk factor for Covid-19 hospital admission. *Clin Infect Dis* Published Online First: 2020. doi:10.1093/cid/ciaa415

38 Timpson NJ, Nordestgaard BG, Harbord RM, *et al.* C-reactive protein levels and body mass index: Elucidating direction of causation through reciprocal Mendelian randomization. *Int J Obes* 2011;**35**:300–8. doi:10.1038/ijo.2010.137

39 Welsh P, Polisecki E, Robertson M, *et al.* Unraveling the directional link between adiposity and inflammation: A bidirectional mendelian randomization approach. *J Clin Endocrinol Metab* 2010;**95**:93–9. doi:10.1210/jc.2009-1064

40 Klovaite J, Benn M, Nordestgaard BG. Obesity as a causal risk factor for deep venous thrombosis: A Mendelian randomization study. *J Intern Med* 2015;**277**:573–84. doi:10.1111/joim.12299

41 Beun R, Kusadasi N, Sikma M, *et al.* Thromboembolic events and apparent heparin resistance in patients infected with SARS-CoV-2. *Int J Lab Hematol* Published Online First: April 2020. doi:10.1111/ijlh.13230

42 Panigada M, Bottino N, Tagliabue P, *et al.* Hypercoagulability of COVID-19 patients in Intensive Care Unit. A Report of Thromboelastography Findings and other Parameters of Hemostasis. *J Thromb Haemost* 2020;:jth.14850. doi:10.1111/jth.14850

43 Zhou F, Yu T, Du R, *et al.* Clinical course and risk factors for mortality of adult inpatients with COVID-19 in Wuhan, China: a retrospective cohort study. *Lancet* 2020;**395**:1054–62. doi:10.1016/S0140-6736(20)30566-3

44 Fry A, Littlejohns TJ, Sudlow C, *et al.* Comparison of Sociodemographic and Health-Related Characteristics of UK Biobank Participants With Those of the General Population. *Am J Epidemiol* 2017;**186**:1026–34. doi:10.1093/aje/kwx246

45 Keyes KM, Westreich D. UK Biobank, big data, and the consequences of non-representativeness. *Lancet* 2019;**393**:1297. doi:10.1016/S0140-6736(18)33067-8

46 Green MJ, Popham F. Interpreting mutual adjustment for multiple indicators of

For peer review only - http://bmjopen.bmj.com/site/about/guidelines.xhtml

socioeconomic position without committing mutual adjustment fallacies. *BMC Public Health* 2019;**19**:10. doi:10.1186/s12889-018-6364-y

For peer review only

Figure Legends

Figure 1. Nonlinear associations of significant continuous variables with COVID-19 and pneumonia

Figure 2. Risk factors for COVID-19 and Pneumonia in the UK Biobank cohort.

Data presented as Risk Ratios (RR) and their 95% CI. Analyses were adjusted for age, sex, ethnicity, deprivation, BMI, FEV1, and walking pace (and DBP for COVID-19 only). Continuous exposures were standardised and presented per 1-SD increment (deprivation index SD=3.01, Cystatin C SD=0.14, BMI SD=4.59, HbA1c SD=5.80, FEV1 SD=0.77 and HDL cholesterol SD=0.37).

For peer review only

For peer review only - http://bmjopen.bmj.com/site/about/guidelines.xhtml

Table 1. Univariable association of baseline risk factors with in hospital COVID-19 in 2020, pneumonia occurring after 2016, and influenza occurring after 2016

	In hospital COVID-19				Pneumonia (after 2016)				Influenza (after 2016)			
	No n=235531	Yes n=397	RR*	P	No n=96605	Yes n=209	RR	P	No n=96720	Yes n=94	RR	P
Age (years) †	66.53 (8.09)	67.55 (8.68)	1.14	0.01	66.52 (8.06)	68.78 (7.60)	1.34	< 0.0001	66.53 (8.06)	66.20 (7.83)	0.96	0.69
Age categories (years), n (%)												
<60	58683 (24.92)	102 (25.69)	1	REF	23707 (24.54)	31 (14.83)	1	REF	23717 (24.52)	21 (22.34)	1	REF
60-64	37589 (15.96)	42 (10.58)	0.64	0.02	15230 (15.77)	24 (11.48)	1.20	0.49	15233 (15.75)	21 (22.34)	1.56	0.15
65-69	42812 (18.18)	58 (14.61)	0.78	0.13	17606 (18.22)	45 (21.53)	1.95	0.004	17632 (18.23)	19 (20.21)	1.22	0.54
70-74	55726 (23.66)	93 (23.43)	0.96	0.78	23233 (24.05)	63 (30.14)	2.07	0.0009	23278 (24.07)	18 (19.15)	0.87	0.67
≥75	40721 (17.29)	102 (25.69)	1.44	0.009	16829 (17.42)	46 (22.01)	2.09	0.002	16860 (17.43)	15 (15.96)	1.00	0.99
Male sex, n (%)	111095 (47.17)	219 (55.16)	1.38	0.001	45548 (47.15)	72 (34.45)	0.59	0.0003	45573 (47.12)	47 (50.00)	1.12	0.58
Ethnicity (%)												
White	224215 (95.20)	356 (89.67)	1	REF	92477 (95.73)	201 (96.17)	1	REF	92588 (95.73)	90 (95.74)	1	REF
Black	3057 (1.30)	14 (3.53)	2.88	0.0001	908 (0.94)	3 (1.44)	1.52	0.47	911 (0.94)	0 (0.00)	0.00	0.97
South Asian	3508 (1.49)	12 (3.02)	2.15	0.009	1605 (1.66)	3 (1.44)	0.86	0.80	1607 (1.66)	1 (1.06)	0.64	0.66
Others	4751 (2.02)	15 (3.78)	1.99	0.009	1615 (1.67)	2 (0.96)	0.57	0.43	1614 (1.67)	3 (3.19)	1.91	0.27
Deprivation index (score)	-1.44 (2.98)	-0.56 (3.37)	1.30	< 0.0001	-1.48 (2.90)	-1.28 (3.07)	1.07	0.31	-1.48 (2.90)	-0.92 (3.32)	1.20	0.06
Current/former smoker, n (%)	106829 (45.36)	224 (56.42)	1.56	< 0.0001	43843 (45.38)	93 (44.50)	0.96	0.80	43891 (45.38)	45 (47.87)	1.11	0.63
Alcohol drinking status, n (%)												
Never	8894 (3.78)	27 (6.80)	1	REF	3714 (3.84)	13 (6.22)	1	REF	3723 (3.85)	4 (4.26)	1	REF
Former	7469 (3.17)	19 (4.79)	0.84	0.56	3090 (3.20)	13 (6.22)	1.20	0.64	3099 (3.20)	4 (4.26)	1.20	0.80
Current	219168 (93.05)	351 (88.41)	0.53	0.001	89801 (92.96)	183 (87.56)	0.58	0.06	89898 (92.95)	86 (91.49)	0.89	0.82
BMI (kg/m²)	27.32 (4.56)	29.12 (5.25)	1.40	< 0.0001	27.41 (4.56)	28.61 (5.58)	1.26	0.0002	27.42 (4.57)	27.53 (4.70)	1.03	0.80
BMI categories, n (%)												
Underweight	787 (0.33)	1 (0.25)	1.17	0.88	305 (0.32)	1 (0.48)	1.65	0.62	306 (0.32)	0 (0.00)	0.00	0.97
Normal	78073 (33.15)	85 (21.41)	1	REF	31182 (32.28)	62 (29.67)	1	REF	31209 (32.27)	35 (37.23)	1	REF
Overweight	101751 (43.20)	172 (43.32)	1.55	0.0009	41867 (43.34)	80 (38.28)	0.96	0.81	41917 (43.34)	30 (31.91)	0.64	0.07
Obese	54920 (23.32)	139 (35.01)	2.32	< 0.0001	23251 (24.07)	66 (31.58)	1.43	0.04	23288 (24.08)	29 (30.85)	1.11	0.68
Body fat-free mass (Kg)	53.76 (11.47)	56.18 (11.50)	1.23	< 0.0001	53.75 (11.49)	52.08 (12.04)	0.86	0.04	53.74 (11.49)	54.61 (12.17)	1.08	0.46
Body fat mass (Kg)	24.49 (9.19)	27.44 (10.35)	1.32	< 0.0001	24.69 (9.21)	27.90 (11.25)	1.35	< 0.0001	24.70 (9.22)	24.31 (9.37)	0.96	0.68
Body fat proportion (percent)	31.01 (8.40)	32.36 (8.47)	1.17	0.001	31.19 (8.41)	34.29 (8.68)	1.44	< 0.0001	31.19 (8.41)	30.51 (8.46)	0.92	0.43
Systolic blood pressure (mmHg)	137.24 (18.07)	138.49 (18.76)	1.07	0.17	137.55 (18.10)	137.55 (18.03)	1.00	1.00	137.55 (18.10)	137.60 (17.40)	1.00	0.98
Diastolic blood pressure (mmHg)	82.08 (9.90)	82.99 (10.47)	1.10	0.07	82.19 (9.90)	80.90 (9.63)	0.88	0.06	82.19 (9.90)	82.79 (10.74)	1.06	0.56
FEV1(litres)	2.86 (0.77)	2.73 (0.78)	0.84	0.0009	2.86 (0.78)	2.53 (0.81)	0.63	< 0.0001	2.86 (0.78)	2.84 (0.88)	0.98	0.84
FVC (litres)	3.79 (0.98)	3.68 (0.97)	0.89	0.03	3.79 (0.99)	3.40 (1.01)	0.65	< 0.0001	3.79 (0.99)	3.73 (1.04)	0.95	0.60
FEV1/FVC	0.76 (0.06)	0.74 (0.08)	0.85	0.0004	0.76 (0.06)	0.74 (0.08)	0.82	0.0007	0.76 (0.06)	0.76 (0.06)	1.04	0.72
Walking pace (%)												
Slow	14887 (6.32)	59 (14.86)	2.29	< 0.0001	6190 (6.41)	29 (13.88)	2.07	0.0005	6209 (6.42)	10 (10.64)	1.78	0.10
Average	123588 (52.47)	213 (53.65)	1	REF	50839 (52.63)	115 (55.02)	1	REF	50908 (52.63)	46 (48.94)	1	REF

For peer review only

For peer review only - http://bmjopen.bmj.com/site/about/guidelines.xhtml

Brisk	97056 (41.21)	125 (31.49)	0.75	0.010	39576 (40.97)	65 (31.10)	0.73	0.04	39603 (40.95)	38 (40.43)	1.06	0.78
Grip strength (Kg) †	31.31 (10.93)	31.36 (10.31)	1.00	0.93	31.21 (10.96)	27.83 (10.83)	0.72	< 0.0001	31.20 (10.96)	31.94 (11.27)	1.07	0.51
Prevalent disease at baseline (%)												
Longstanding illness, n (%)	68208 (28.96)	156 (39.29)	1.59	< 0.0001	28899 (29.91)	89 (42.58)	1.74	< 0.0001	28954 (29.94)	34 (36.17)	1.33	0.19
Diabetes	10969 (4.66)	38 (9.57)	2.16	< 0.0001	4575 (4.74)	20 (9.57)	2.12	0.001	4591 (4.75)	4 (4.26)	0.89	0.82
CVD	11238 (4.77)	35 (8.82)	1.93	0.0002	4870 (5.04)	18 (8.61)	1.77	0.02	4884 (5.05)	4 (4.26)	0.84	0.73
Cancer	15849 (6.75)	32 (8.12)	1.22	0.28	6557 (6.81)	27 (12.92)	2.03	0.0006	6573 (6.82)	11 (11.70)	1.81	0.06
Depression	45781 (19.44)	68 (17.13)	0.86	0.25	18651 (19.31)	34 (16.27)	0.81	0.27	18667 (19.30)	18 (19.15)	0.99	0.97
CKD stages 3-5	293 (0.12)	2 (0.50)	4.04	0.049	128 (0.13)	0 (0.00)	0.00	0.97	128 (0.13)	0 (0.00)	0.00	0.98
SLE	274 (0.12)	0 (0.00)	0.00	0.96	125 (0.13)	0 (0.00)	0.00	0.97	125 (0.13)	0 (0.00)	0.00	0.98
Asthma	27722 (11.77)	54 (13.60)	1.18	0.26	11433 (11.83)	34 (16.27)	1.45	0.049	11454 (11.84)	13 (13.83)	1.19	0.55
Sleep apnoea	718 (0.30)	4 (1.01)	3.32	0.02	317 (0.33)	0 (0.00)	0.00	0.97	317 (0.33)	0 (0.00)	0.00	0.97
COPD	536 (0.23)	3 (0.76)	3.33	0.04	241 (0.25)	0 (0.00)	0.00	0.96	240 (0.25)	1 (1.06)	4.31	0.15
Bronchitis	2515 (1.07)	8 (2.02)	1.90	0.07	1085 (1.12)	6 (2.87)	2.59	0.02	1087 (1.12)	4 (4.26)	3.90	0.008
Pneumonia	2938 (1.25)	8 (2.02)	1.63	0.17	1152 (1.19)	4 (1.91)	1.61	0.34	1155 (1.19)	1 (1.06)	0.89	0.91
Other respiratory disease	1243 (0.53)	3 (0.76)	1.43	0.53	511 (0.53)	1 (0.48)	0.90	0.92	512 (0.53)	0 (0.00)	0.00	0.98
Medication at baseline, n (%)												
Statin	36314 (15.42)	98 (24.69)	1.80	< 0.0001	15358 (15.90)	39 (18.66)	1.21	0.28	15389 (15.91)	8 (8.51)	0.49	0.055
BP medication	37590 (15.96)	105 (26.45)	1.89	< 0.0001	15719 (16.27)	34 (16.27)	1.00	1.00	15741 (16.27)	12 (12.77)	0.75	0.36
Steroid	876 (0.37)	1 (0.25)	0.68	0.70	354 (0.37)	1 (0.48)	1.31	0.79	355 (0.37)	0 (0.00)	0.00	0.98
Biomarker at baseline												
Total cholesterol (mmol/L) †	5.62 (4.92-6.34)	5.48 (4.68-6.21)	0.85	0.001	5.62 (4.91-6.34)	5.62 (4.84-6.30)	0.99	0.94	5.62 (4.91-6.34)	5.62 (5.04-6.37)	1.07	0.49
HDL cholesterol (mmol/L)	1.40 (1.17-1.67)	1.29 (1.09-1.54)	0.71	< 0.0001	1.39 (1.17-1.67)	1.41 (1.14-1.70)	1.01	0.87	1.39 (1.17-1.67)	1.36 (1.12-1.64)	0.92	0.44
Cystatin C (mg/L)	0.88 (0.80-0.97)	0.92 (0.83-1.02)	1.34	< 0.0001	0.88 (0.80-0.97)	0.89 (0.80-1.01)	1.12	0.07	0.88 (0.80-0.97)	0.91 (0.82-0.98)	1.03	0.76
HbA1c (mmol/mol) †	35.10 (32.60-37.60)	36.20 (33.60-39.60)	1.30	< 0.0001	35.10 (32.60-37.60)	36.00 (33.50-39.00)	1.22	< 0.0001	35.10 (32.60-37.60)	35.10 (32.32-38.30)	1.08	0.39
CRP (mg/L) †	1.25 (0.63-2.56)	1.58 (0.86-3.28)	1.15	0.0001	1.27 (0.64-2.59)	1.37 (0.84-3.27)	1.14	0.01	1.27 (0.64-2.59)	1.34 (0.70-2.93)	1.07	0.47
Rheumatoid factor (IU/ml)	3.16 (3.16-3.16)	3.16 (3.16-3.16)	1.02	0.64	3.16 (3.16-3.16)	3.16 (3.16-3.16)	1.04	0.50	3.16 (3.16-3.16)	3.16 (3.16-3.16)	1.04	0.64
Red cell distribution width (%)	13.30 (12.90-13.80)	13.32 (12.97-13.95)	1.12	0.01	13.30 (12.89-13.80)	13.38 (12.97-14.02)	1.15	0.02	13.30 (12.89-13.80)	13.27 (12.97-13.70)	0.90	0.36
White cell count (10^9 cells/L)	6.60 (5.61-7.80)	6.90 (5.84-8.26)	1.17	0.001	6.63 (5.64-7.80)	6.90 (5.80-8.29)	1.15	0.03	6.63 (5.64-7.80)	6.60 (5.50-7.76)	1.02	0.82
Neutrophil count (10^9 cells/L)	4.00 (3.25-4.90)	4.12 (3.29-5.13)	1.12	0.02	4.01 (3.26-4.92)	4.11 (3.41-5.15)	1.10	0.13	4.01 (3.26-4.92)	4.02 (3.30-5.05)	0.99	0.93
Lymphocyte count (10^9 cells/L)	1.88 (1.52-2.29)	1.90 (1.55-2.40)	1.13	0.009	1.88 (1.52-2.30)	1.92 (1.60-2.36)	1.11	0.12	1.88 (1.52-2.29)	1.85 (1.51-2.53)	1.04	0.68
Monocyte count (10^9 cells/L)	0.45 (0.36-0.56)	0.47 (0.39-0.59)	1.13	0.01	0.45 (0.37-0.57)	0.48 (0.37-0.59)	1.07	0.31	0.45 (0.37-0.57)	0.50 (0.40-0.57)	1.14	0.17

Numbers represent number (%) for categorical variables, mean (SD) for gaussian continuous variables, and median (25-75th percentiles) for skewed variables

*Univariable relative risk ratio per 1 standard deviation for continuous variables and using comparator group for categorical variables.

†Evidence for non-linear association with COVID-19 (see supplementary table 3)

For peer review only - http://bmjopen.bmj.com/site/about/guidelines.xhtml

Table 2. Association of risk factors for COVID-19 and pneumonia in UK Biobank

	In hospital COVID-19				Pneumonia (after 2016)			
	Model 1		Model 2		Model 1		Model 2	
	RR (95% CI)	P	RR (95% CI)	P	RR (95% CI)	P	RR (95% CI)	P
Age (per 5 years)	1.12 (1.05, 1.19)	0.0007	1.04 (0.96, 1.11)	0.32	1.21 (1.11, 1.33)	< 0.0001	1.12 (1.01, 1.24)	0.03
Male Sex	1.36 (1.11, 1.66)	0.003	1.72 (1.25, 2.35)	0.0008	0.58 (0.44, 0.77)	0.0002	0.80 (0.56, 1.16)	0.24
Ethnicity								
White	1 (Reference)	-	1 (Reference)	-	1 (Reference)	-	1 (Reference)	-
Black	2.32 (1.33, 4.04)	0.003	2.00 (1.13, 3.53)	0.02	1.63 (0.51, 5.21)	0.41	1.20 (0.37, 3.89)	0.76
South Asian	1.98 (1.10, 3.55)	0.02	1.73 (0.94, 3.17)	0.08	0.96 (0.31, 3.05)	0.95	0.66 (0.20, 2.14)	0.49
Others	1.83 (1.08, 3.10)	0.03	1.70 (0.99, 2.91)	0.053	0.63 (0.15, 2.55)	0.51	0.52 (0.13, 2.15)	0.37
Deprivation index (per 1 SD)	1.27 (1.16, 1.39)	< 0.0001	1.17 (1.06, 1.29)	0.001	1.10 (0.96, 1.26)	0.18	1.04 (0.90, 1.20)	0.62
Current/former smoker	1.45 (1.19, 1.79)	0.0003	1.39 (1.13, 1.71)	0.002	0.95 (0.72, 1.25)	0.71	0.89 (0.67, 1.19)	0.44
Alcohol drinking status								
Never	1 (Reference)	-	-	-	1 (Reference)	-	-	-
Former	0.90 (0.49, 1.65)	0.74	-	-	1.28 (0.58, 2.79)	0.54	-	-
Current	0.65 (0.43, 1.00)	0.05	-	-	0.66 (0.37, 1.18)	0.16	-	-
BMI (per 1 SD)	1.36 (1.25, 1.48)	< 0.0001	1.28 (1.16, 1.40)	< 0.0001	1.25 (1.11, 1.41)	0.0002	1.16 (1.02, 1.32)	0.03
BMI categories								
Underweight	1.17 (0.16, 8.59)	0.88	-	-	1.43 (0.20, 10.04)	0.73	-	-
Normal	1 (Reference)	-	-	-	1 (Reference)	-	-	-
Overweight	1.43 (1.10, 1.87)	0.008	-	-	1.01 (0.72, 1.42)	0.94	-	-
Obese	2.08 (1.58, 2.74)	< 0.0001	-	-	1.44 (1.01, 2.05)	0.04	-	-
Body fat-free mass (per 1 SD)	1.39 (1.17, 1.64)	0.0002	-	-	1.42 (1.10, 1.84)	0.008	-	-
Body fat mass (per 1 SD)	1.37 (1.25, 1.50)	< 0.0001	-	-	1.27 (1.12, 1.44)	0.0002	-	-
Body fat percent (per 1 SD)	1.58 (1.37, 1.81)	< 0.0001	-	-	1.34 (1.11, 1.62)	0.002	-	-
Systolic blood pressure (per 1 SD)	1.01 (0.91, 1.12)	0.89	-	-	0.94 (0.81, 1.08)	0.39	-	-
Diastolic blood pressure (per 1 SD)	1.06 (0.96, 1.17)	0.25	-	-	0.90 (0.79, 1.04)	0.16	-	-
FEV1 (per 1 SD)	0.76 (0.66, 0.87)	< 0.0001	0.84 (0.73, 0.97)	0.02	0.70 (0.58, 0.86)	0.0005	0.75 (0.61, 0.92)	0.006
FVC (per 1 SD)	0.80 (0.70, 0.93)	0.003	-	-	0.72 (0.59, 0.90)	0.003	-	-
FEV1/FVC (per 1 SD)	0.89 (0.81, 0.98)	0.01	-	-	0.84 (0.74, 0.95)	0.007	-	-
Walking pace								
Slow	1.99 (1.48, 2.68)	< 0.0001	1.53 (1.12, 2.08)	0.007	1.91 (1.26, 2.90)	0.002	1.60 (1.04, 2.46)	0.03
Average	1 (Reference)	-	1 (Reference)	-	1 (Reference)	-	1 (Reference)	-

For peer review only - http://bmjopen.bmj.com/site/about/guidelines.xhtml

Brisk	0.80 (0.64, 1.00)	0.051	0.95 (0.75, 1.20)	0.65	0.78 (0.57, 1.06)	0.11	0.88 (0.64, 1.21)	0.42
Grip strength (per 1 SD)	0.87 (0.75, 1.01)	0.07	0.94 (0.81, 1.10)	0.45	0.85 (0.68, 1.07)	0.16	0.96 (0.76, 1.22)	0.76
Prevalent disease at baseline								
Longstanding illness	1.44 (1.17, 1.77)	0.0005	1.16 (0.93, 1.44)	0.19	1.65 (1.25, 2.18)	0.0004	1.40 (1.04, 1.88)	0.03
Diabetes	1.72 (1.22, 2.43)	0.002	1.24 (0.87, 1.77)	0.23	2.06 (1.29, 3.30)	0.002	1.59 (0.98, 2.59)	0.06
CVD	1.56 (1.09, 2.23)	0.02	1.22 (0.85, 1.76)	0.29	1.64 (1.00, 2.70)	0.052	1.35 (0.81, 2.24)	0.25
Cancer	1.22 (0.84, 1.76)	0.29	1.20 (0.83, 1.74)	0.33	1.72 (1.14, 2.60)	0.009	1.72 (1.14, 2.60)	0.01
Depression	0.96 (0.73, 1.25)	0.74	1.01 (0.77, 1.32)	0.94	0.81 (0.56, 1.18)	0.27	0.86 (0.59, 1.25)	0.42
Asthma	1.19 (0.89, 1.59)	0.23	1.04 (0.78, 1.40)	0.78	1.44 (0.99, 2.09)	0.054	1.25 (0.85, 1.82)	0.25
COPD	2.80 (0.89, 8.82)	0.08	1.81 (0.57, 5.79)	0.31	-	-	-	-
Bronchitis	1.65 (0.82, 3.36)	0.16	1.26 (0.61, 2.58)	0.53	2.23 (0.98, 5.06)	0.055	1.77 (0.77, 4.07)	0.18
Pneumonia	1.57 (0.77, 3.17)	0.21	1.50 (0.74, 3.05)	0.26	1.53 (0.57, 4.14)	0.40	1.47 (0.54, 3.99)	0.45
Other respiratory disease	1.36 (0.43, 4.26)	0.60	1.21 (0.38, 3.82)	0.74	0.84 (0.12, 6.04)	0.86	0.76 (0.10, 5.52)	0.78
Medication at baseline								
Statin	1.53 (1.20, 1.95)	0.0006	1.24 (0.97, 1.59)	0.09	1.08 (0.75, 1.55)	0.69	0.91 (0.62, 1.32)	0.62
BP medication	1.62 (1.28, 2.06)	< 0.0001	1.33 (1.04, 1.70)	0.02	0.96 (0.66, 1.41)	0.85	0.82 (0.55, 1.21)	0.31
Steroid	0.63 (0.09, 4.57)	0.65	0.53 (0.07, 3.83)	0.53	1.20 (0.17, 8.63)	0.86	1.03 (0.14, 7.53)	0.97
Biomarker								
Total cholesterol (per 1 SD)	0.89 (0.81, 0.99)	0.03	0.93 (0.85, 1.03)	0.16	0.94 (0.82, 1.08)	0.40	0.98 (0.85, 1.12)	0.76
HDL cholesterol (per 1 SD)	0.73 (0.65, 0.83)	< 0.0001	0.83 (0.73, 0.95)	0.005	0.88 (0.76, 1.03)	0.11	0.98 (0.84, 1.16)	0.84
Cystatin C (per 1 SD)	1.27 (1.16, 1.39)	< 0.0001	1.13 (1.02, 1.25)	0.02	1.08 (0.94, 1.24)	0.29	0.96 (0.83, 1.12)	0.59
HbA1c (per 1 SD)	1.23 (1.15, 1.32)	< 0.0001	1.14 (1.05, 1.23)	0.001	1.19 (1.07, 1.33)	0.001	1.11 (0.99, 1.25)	0.07
CRP (per 1 SD)	1.14 (1.06, 1.22)	0.0006	1.03 (0.95, 1.13)	0.45	1.11 (1.00, 1.24)	0.04	1.03 (0.91, 1.16)	0.67
Rheumatoid factor (per 1 SD)	1.02 (0.93, 1.12)	0.70	1.01 (0.92, 1.11)	0.82	1.03 (0.91, 1.15)	0.68	1.02 (0.90, 1.15)	0.76
Red cell distribution width (per 1 SD)	1.08 (0.99, 1.19)	0.09	1.03 (0.94, 1.13)	0.55	1.12 (0.99, 1.27)	0.07	1.07 (0.95, 1.22)	0.27
White cell count (per 1 SD)	1.15 (1.05, 1.27)	0.003	1.04 (0.94, 1.15)	0.44	1.15 (1.01, 1.32)	0.03	1.07 (0.93, 1.23)	0.32
Neutrophil count (per 1 SD)	1.12 (1.02, 1.23)	0.02	1.03 (0.93, 1.13)	0.61	1.11 (0.97, 1.27)	0.12	1.04 (0.91, 1.19)	0.57
Lymphocyte count (per 1 SD)	1.11 (1.01, 1.23)	0.02	1.03 (0.94, 1.14)	0.53	1.07 (0.94, 1.23)	0.28	1.02 (0.89, 1.17)	0.76
Monocyte count (per 1 SD)	1.09 (0.99, 1.20)	0.08	1.01 (0.92, 1.12)	0.79	1.11 (0.97, 1.28)	0.12	1.06 (0.92, 1.21)	0.43

Data presented as risk ratios and their 95% CI. continuous exposures were standardised and presented as 1-standard deviation (SD) increment. Variables with associations denoted "-" are excluded from the model due to non-significance in previous model and /or collinearity with another variable. Analyses were adjusted for Model 1 (age, sex, ethnicity and deprivation) and model 2 (model 1 plus BMI, FEV1, and walking pace (and also grip strength and DBP for COVID-19 only))

For peer review only - http://bmjopen.bmj.com/site/about/guidelines.xhtml

Table 3. Population attributable fractions of COVID-19 in the UK Biobank

	COVID-19		Pneumonia after 2016	
	**RR (95% CI)**	**PAF (%)**	**RR (95% CI)**	**PAF (%)**
Current/former smoker	1.39 (1.13 to 1.70)	14.94	0.91 (0.69 to 1.20)	-4.15
High deprivation index	1.45 (1.16 to 1.82)	8.33	1.00 (0.71 to 1.40)	-0.10
Male	1.18 (0.94 to 1.50)	8.00	0.64 (0.46 to 0.90)	-20.65
Age > 65 years	1.18 (0.94 to 1.46)	6.33	1.29 (0.96 to 1.73)	9.88
BMI > 30 kg/m^2	1.29 (1.03 to 1.61)	6.25	1.14 (0.83 to 1.57)	3.17
High HbA1c	1.29 (1.02 to 1.62)	5.26	1.37 (1.00 to 1.88)	6.66
Low HDL cholesterol	1.27 (1.00 to 1.61)	5.15	1.34 (0.95 to 1.91)	6.45
BP medication	1.34 (1.05 to 1.71)	5.11	0.88 (0.60 to 1.29)	-2.19
High Cystatin C	1.24 (0.98 to 1.56)	4.48	1.12 (0.80 to 1.57)	2.39
Slow walking pace	1.66 (1.23 to 2.24)	4.02	1.78 (1.17 to 2.70)	4.69
Ethnicity: Black	1.87 (1.32 to 2.65)	4.00	0.70 (0.34 to 1.45)	- 2.00
Low FEV1	1.15 (0.88 to 1.50)	2.93	1.64 (1.18 to 2.28)	11.23

Estimated using prevalence in the study sample and RR shown in this table.

All factors shown are mutually adjusted. Continuous variables were categorised with the top 20% as the at-risk group.

RR shown in this table may be over adjusted, e.g. cystatin C and HDL cholesterol may be downstream factors of BMI.

For peer review only

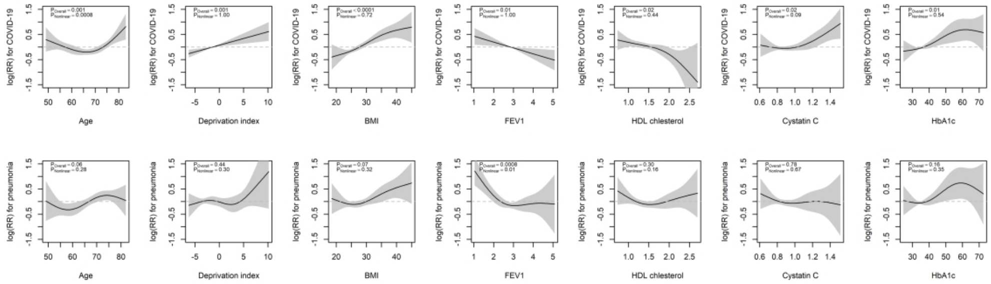

Nonlinear associations of significant continuous variables with COVID-19 and pneumonia

355x110mm (300 x 300 DPI)

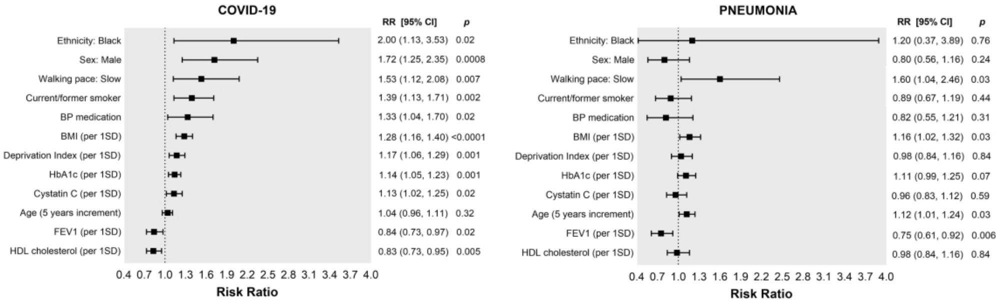

Risk factors for COVID-19 and Pneumonia in the UK Biobank cohort.Data presented as Risk Ratios (RR) and their 95% CI. Analyses were adjusted for age, sex, ethnicity, deprivation, BMI, FEV1, and walking pace (and DBP for COVID-19 only). Continuous exposures were standardised and presented per 1-SD increment (deprivation index SD=3.01, Cystatin C SD=0.14, BMI SD=4.59, HbA1c SD=5.80, FEV1 SD=0.77 and HDL cholesterol SD=0.37).

234x71mm (300 x 300 DPI)

Modifiable and non-modifiable risk factors for COVID-19, and comparison to risk factors for influenza and pneumonia: results from a UK Biobank prospective cohort study

Supplementary Material

Frederick K Ho*, Carlos A Celis-Morales*, Stuart R Gray, S Vittal Katikireddi, Claire L Niedzwiedz, Claire Hastie, Lyn D Ferguson, Colin Berry, Daniel F Mackay, Jason MR Gill, Jill P Pell**, Naveed Sattar MD**, Paul Welsh**

*Joint first authors

**Joint senior

For peer review only

SUPPLEMENTARY MATERIAL

Supplementary Table 1. Univariable association of baseline risk factors pneumonia and influenza occurring any time after baseline

	COVID-19 in all settings				Pneumonia since baseline				Influenza since baseline			
	**No n=235410**	**Yes n=518**	**RR***	**P**	**No n=94015**	**Yes n=2799**	**RR***	**P**	**No n=95452**	**Yes n=1362**	**RR***	**P**
Age (years)	66.53 (8.09)	67.04 (8.93)	1.07	0.15	66.51 (8.06)	67.24 (8.18)	1.09	< 0.0001	66.54 (8.06)	65.63 (8.17)	0.90	< 0.0001
Age categories (years)												
<60	58639 (24.91)	146 (28.19)	1	REF	23107 (24.58)	631 (22.54)	1	REF	23349 (24.46)	389 (28.56)	1	REF
60-64	37569 (15.96)	62 (11.97)	0.66	0.007	14884 (15.83)	370 (13.22)	0.91	0.16	15027 (15.74)	227 (16.67)	0.91	0.25
65-69	42802 (18.18)	68 (13.13)	0.64	0.002	17128 (18.22)	523 (18.69)	1.11	0.06	17392 (18.22)	259 (19.02)	0.90	0.17
70-74	55711 (23.67)	108 (20.85)	0.78	0.049	22584 (24.02)	712 (25.44)	1.15	0.010	23000 (24.10)	296 (21.73)	0.78	0.0009
≥75	40689 (17.28)	134 (25.87)	1.32	0.02	16312 (17.35)	563 (20.11)	1.26	< 0.0001	16684 (17.48)	191 (14.02)	0.69	< 0.0001
Male sex, n (%)	111029 (47.16)	285 (55.02)	1.37	0.0004	44786 (47.64)	834 (29.80)	0.48	< 0.0001	45041 (47.19)	579 (42.51)	0.83	0.0006
Ethnicity, n (%)												
White	224116 (95.20)	455 (87.84)	1	REF	90008 (95.74)	2670 (95.39)	1	REF	91423 (95.78)	1255 (92.14)	1	REF
Black	3045 (1.29)	26 (5.02)	4.18	< 0.0001	879 (0.93)	32 (1.14)	1.22	0.26	899 (0.94)	12 (0.88)	0.97	0.92
South Asian	3502 (1.49)	18 (3.47)	2.52	0.0001	1546 (1.64)	62 (2.22)	1.34	0.02	1560 (1.63)	48 (3.52)	2.20	< 0.0001
Others	4747 (2.02)	19 (3.67)	1.97	0.004	1582 (1.68)	35 (1.25)	0.75	0.09	1570 (1.64)	47 (3.45)	2.15	< 0.0001
Deprivation index (score)	-1.44 (2.98)	-0.50 (3.39)	1.32	< 0.0001	-1.49 (2.89)	-1.19 (3.07)	1.10	< 0.0001	-1.49 (2.89)	-0.98 (3.16)	1.18	< 0.0001
Current/former smoker, n (%)	106773 (45.36)	280 (54.05)	1.42	< 0.0001	42567 (45.28)	1369 (48.91)	1.15	0.0001	43261 (45.32)	675 (49.56)	1.18	0.002
Alcohol drinking status, n (%)												
Never	8885 (3.77)	36 (6.95)	1	REF	3573 (3.80)	154 (5.50)	1	REF	3668 (3.84)	59 (4.33)	1	REF
Former	7464 (3.17)	24 (4.63)	0.79	0.38	2963 (3.15)	140 (5.00)	1.09	0.44	3045 (3.19)	58 (4.26)	1.18	0.37
Current	219061 (93.06)	458 (88.42)	0.52	0.0001	87479 (93.05)	2505 (89.50)	0.67	< 0.0001	88739 (92.97)	1245 (91.41)	0.87	0.31
BMI (kg/m^2)	27.31 (4.56)	29.15 (5.29)	1.40	< 0.0001	27.40 (4.55)	27.88 (5.01)	1.10	< 0.0001	27.41 (4.56)	27.74 (4.81)	1.07	0.009
BMI categories, n (%)												
Underweight	787 (0.33)	1 (0.19)	0.87	0.89	295 (0.31)	11 (0.39)	1.29	0.40	306 (0.32)	0 (0.00)	0.00	0.92
Normal	78044 (33.15)	114 (22.01)	1	REF	30371 (32.30)	873 (31.19)	1	REF	30821 (32.29)	423 (31.06)	1	REF
Overweight	101703 (43.20)	220 (42.47)	1.48	0.0007	40839 (43.44)	1108 (39.59)	0.95	0.21	41388 (43.36)	559 (41.04)	0.98	0.80
Obese	54876 (23.31)	183 (35.33)	2.28	< 0.0001	22510 (23.94)	807 (28.83)	1.24	< 0.0001	22937 (24.03)	380 (27.90)	1.20	0.008
Body fat-free mass (Kg)	53.76 (11.47)	56.39 (11.55)	1.25	< 0.0001	53.84 (11.50)	50.54 (10.66)	0.74	< 0.0001	53.75 (11.50)	52.98 (11.20)	0.93	0.01
Body fat mass (Kg)	24.49 (9.19)	27.48 (10.59)	1.32	< 0.0001	24.64 (9.19)	26.67 (9.89)	1.21	< 0.0001	24.69 (9.21)	25.47 (9.56)	1.08	0.002
Body fat proportion (percent)	31.01 (8.40)	32.27 (8.56)	1.16	0.0007	31.11 (8.40)	34.05 (8.28)	1.40	< 0.0001	31.18 (8.41)	32.09 (8.32)	1.11	< 0.0001
Systolic blood pressure (mmHg)	137.24 (18.07)	138.40 (18.89)	1.07	0.14	137.60 (18.11)	135.90 (17.67)	0.91	< 0.0001	137.59 (18.10)	135.00 (17.69)	0.86	< 0.0001
Diastolic blood pressure (mmHg)	82.07 (9.90)	83.13 (10.49)	1.11	0.02	82.22 (9.90)	81.22 (9.60)	0.91	< 0.0001	82.20 (9.89)	81.31 (10.16)	0.91	0.0010
FEV1 (litres)	2.86 (0.77)	2.75 (0.78)	0.86	0.0007	2.87 (0.78)	2.57 (0.73)	0.67	< 0.0001	2.86 (0.78)	2.73 (0.78)	0.84	< 0.0001
FVC (litres)	3.79 (0.98)	3.68 (0.97)	0.89	0.01	3.80 (0.99)	3.44 (0.91)	0.68	< 0.0001	3.79 (0.99)	3.63 (0.96)	0.85	< 0.0001
FEV1/FVC	0.76 (0.06)	0.75 (0.07)	0.88	0.002	0.76 (0.06)	0.75 (0.07)	0.89	< 0.0001	0.76 (0.06)	0.75 (0.07)	0.95	0.045
Walking pace, n (%)												
Slow	14872 (6.32)	74 (14.29)	2.15	< 0.0001	5895 (6.27)	324 (11.58)	1.74	< 0.0001	6098 (6.39)	121 (8.88)	1.35	0.002
Average	123516 (52.47)	285 (55.02)	1	REF	49425 (52.57)	1529 (54.63)	1	REF	50222 (52.61)	732 (53.74)	1	REF

For peer review only - http://bmjopen.bmj.com/site/about/guidelines.xhtml

Brisk	97022 (41.21)	159 (30.69)	0.71	0.0006	38695 (41.16)	946 (33.80)	0.80	< 0.0001	39132 (41.00)	509 (37.37)	0.89	0.050
Grip strength (Kg)	31.31 (10.93)	31.44 (10.39)	1.01	0.79	31.31 (10.97)	27.46 (9.99)	0.69	< 0.0001	31.22 (10.96)	29.99 (10.96)	0.89	< 0.0001
Prevalent disease at baseline, n (%)												
Longstanding illness	68156 (28.95)	208 (40.15)	1.64	< 0.0001	27777 (29.55)	1211 (43.27)	1.78	< 0.0001	28500 (29.86)	488 (35.83)	1.31	< 0.0001
Diabetes	10959 (4.66)	48 (9.27)	2.09	< 0.0001	4386 (4.67)	209 (7.47)	1.62	< 0.0001	4510 (4.72)	85 (6.24)	1.34	0.009
CVD	11229 (4.77)	44 (8.49)	1.85	< 0.0001	4689 (4.99)	199 (7.11)	1.44	< 0.0001	4805 (5.03)	83 (6.09)	1.22	0.08
Cancer	15841 (6.75)	40 (7.77)	1.16	0.36	6339 (6.76)	245 (8.80)	1.32	< 0.0001	6480 (6.81)	104 (7.66)	1.13	0.22
Depression	45765 (19.44)	84 (16.22)	0.80	0.06	18180 (19.34)	505 (18.04)	0.92	0.09	18417 (19.29)	268 (19.68)	1.02	0.72
CKD stages 3-5	291 (0.12)	4 (0.77)	6.22	0.0003	122 (0.13)	6 (0.21)	1.62	0.23	125 (0.13)	3 (0.22)	1.67	0.37
SLE	274 (0.12)	0 (0.00)	0.00	0.96	115 (0.12)	10 (0.36)	2.77	0.001	123 (0.13)	2 (0.15)	1.14	0.85
Asthma	27705 (11.77)	71 (13.71)	1.19	0.17	10897 (11.59)	570 (20.36)	1.90	< 0.0001	11250 (11.79)	217 (15.93)	1.41	< 0.0001
Sleep apnoea	718 (0.30)	4 (0.77)	2.54	0.06	301 (0.32)	16 (0.57)	1.75	0.02	311 (0.33)	6 (0.44)	1.35	0.46
COPD	535 (0.23)	4 (0.77)	3.40	0.01	220 (0.23)	21 (0.75)	3.03	< 0.0001	231 (0.24)	10 (0.73)	2.96	0.0006
Bronchitis	2514 (1.07)	9 (1.74)	1.64	0.14	1011 (1.08)	80 (2.86)	2.58	< 0.0001	1061 (1.11)	30 (2.20)	1.98	0.0002
Pneumonia	2935 (1.25)	11 (2.12)	1.72	0.08	1115 (1.19)	41 (1.46)	1.23	0.18	1140 (1.19)	16 (1.17)	0.98	0.95
Other respiratory disease	1242 (0.53)	4 (0.77)	1.47	0.45	489 (0.52)	23 (0.82)	1.56	0.03	508 (0.53)	4 (0.29)	0.55	0.23
Medication at baseline, n (%)												
Statin	36295 (15.42)	117 (22.59)	1.60	< 0.0001	14836 (15.78)	561 (20.04)	1.33	< 0.0001	15161 (15.88)	236 (17.33)	1.11	0.15
BP medication	37560 (15.96)	135 (26.06)	1.85	< 0.0001	15249 (16.22)	504 (18.01)	1.13	0.01	15504 (16.24)	249 (18.28)	1.15	0.04
Steroid	876 (0.37)	1 (0.19)	0.52	0.51	334 (0.36)	21 (0.75)	2.05	0.0009	352 (0.37)	3 (0.22)	0.60	0.37
Biomarker at baseline												
Total cholesterol (mmol/L)	5.62 (4.92-6.34)	5.45 (4.71-6.20)	0.84	< 0.0001	5.63 (4.91-6.35)	5.52 (4.80-6.26)	0.92	< 0.0001	5.63 (4.91-6.34)	5.51 (4.83-6.26)	0.92	0.0010
HDL cholesterol (mmol/L)	1.40 (1.17-1.67)	1.29 (1.09-1.53)	0.71	< 0.0001	1.39 (1.17-1.67)	1.42 (1.18-1.70)	1.05	0.004	1.40 (1.17-1.67)	1.37 (1.15-1.65)	0.93	0.01
Cystatin C (mg/L)	0.88 (0.80-0.97)	0.93 (0.83-1.02)	1.35	< 0.0001	0.88 (0.80-0.97)	0.88 (0.79-0.99)	1.05	0.01	0.88 (0.80-0.97)	0.89 (0.80-0.99)	1.08	0.004
HbA1c (mmol/mol)	35.10 (32.60-37.60)	35.90 (33.40-39.38)	1.26	< 0.0001	35.10 (32.60-37.60)	35.60 (33.10-38.50)	1.16	< 0.0001	35.10 (32.60-37.60)	35.30 (32.80-38.20)	1.08	0.0005
CRP (mg/L)	1.25 (0.63-2.56)	1.67 (0.87-3.23)	1.26	< 0.0001	1.26 (0.63-2.57)	1.54 (0.78-3.19)	1.16	< 0.0001	1.26 (0.64-2.58)	1.46 (0.71-3.04)	1.08	0.0005
Rheumatoid factor (IU/ml)	3.16 (3.16-3.16)	3.16 (3.16-3.16)	1.15	< 0.0001	3.16 (3.16-3.16)	3.16 (3.16-3.16)	1.13	< 0.0001	3.16 (3.16-3.16)	3.16 (3.16-3.16)	1.11	< 0.0001
Red cell distribution width (%)	13.30 (12.90-13.80)	13.34 (12.97-13.94)	1.03	0.50	13.30 (12.89-13.80)	13.39 (12.93-13.91)	1.04	0.03	13.30 (12.89-13.80)	13.30 (12.90-13.80)	1.04	0.07
White cell count (10^9 cells/Litre)	6.60 (5.61-7.80)	6.82 (5.82-8.20)	1.14	0.0005	6.62 (5.63-7.80)	6.89 (5.83-8.18)	1.11	< 0.0001	6.63 (5.64-7.80)	6.86 (5.75-8.07)	1.02	0.39
Neutrophil count (10^9 cells/Litre)	4.00 (3.25-4.90)	4.11 (3.30-5.11)	1.15	0.0007	4.00 (3.26-4.91)	4.20 (3.42-5.10)	1.16	< 0.0001	4.01 (3.26-4.92)	4.19 (3.32-5.07)	1.12	< 0.0001
Lymphocyte count (10^9 cells/Litre)	1.88 (1.52-2.29)	1.90 (1.55-2.40)	1.11	0.01	1.88 (1.52-2.29)	1.92 (1.55-2.39)	1.14	< 0.0001	1.88 (1.52-2.29)	1.90 (1.53-2.36)	1.10	0.0003
Monocyte count (10^9 cells/Litre)	0.45 (0.36-0.56)	0.48 (0.38-0.58)	1.11	0.01	0.45 (0.37-0.57)	0.45 (0.37-0.57)	1.10	< 0.0001	0.45 (0.37-0.57)	0.46 (0.37-0.58)	1.07	0.01

Numbers represent number (%) for categorical variables, mean (SD) for gaussian continuous variables, and median (25-75th percentiles) for skewed variables

*Univariable relative risk ratio per 1 standard deviation for continuous variables and using comparator group for categorical variables.

For peer review only - http://bmjopen.bmj.com/site/about/guidelines.xhtml

Supplementary table 2. Sensitivity analysis for pneumonia occurring after 2015.

	Pneumonia (after 2015)				Model 1		Model 2	
	**No n=117181**	**Yes n=767**	**RR**	**P**	**RR (95% CI)**	**P**	**RR (95% CI)**	**P**
Mean (SD) Age	66.52 (8.06)	68.44 (7.87)	1.28	< 0.0001	1.18 (1.12, 1.24)	< 0.0001	1.10 (1.04, 1.17)	0.001
Age categories								
<50	23642 (24.57)	96 (15.79)	1	REF	-	-	-	-
50-54	15174 (15.77)	80 (13.16)	1.30	0.09	-	-	-	-
55-59	17523 (18.21)	128 (21.05)	1.79	< 0.0001	-	-	-	-
60-64	23142 (24.05)	154 (25.33)	1.63	0.0002	-	-	-	-
≥65	16725 (17.38)	150 (24.67)	2.20	< 0.0001	-	-	-	-
Male	45416 (47.21)	204 (33.55)	0.57	< 0.0001	0.56 (0.47, 0.66)	< 0.0001	0.72 (0.58, 0.89)	0.003
Ethnicity								
White	92091 (95.72)	587 (96.55)	1	REF	1 (Reference)	-	1 (Reference)	-
Black	906 (0.94)	5 (0.82)	0.87	0.75	0.88 (0.36, 2.13)	0.78	0.72 (0.29, 1.75)	0.47
South Asian	1600 (1.66)	8 (1.32)	0.79	0.50	0.85 (0.42, 1.71)	0.65	0.66 (0.32, 1.34)	0.25
Others	1609 (1.67)	8 (1.32)	0.78	0.49	0.82 (0.41, 1.66)	0.59	0.72 (0.36, 1.45)	0.36
Mean (SD) deprivation index	-1.49 (2.90)	-1.22 (3.01)	1.09	0.03	1.12 (1.04, 1.22)	0.004	1.07 (0.98, 1.16)	0.11
Current/former smoker	43642 (45.36)	294 (48.36)	1.13	0.14	0.95 (0.72, 1.25)	0.71	1.07 (0.91, 1.26)	0.43
Alcohol drinking status								
Never	3696 (3.84)	31 (5.10)	1	REF	1 (Reference)	-	-	-
Former	3077 (3.20)	26 (4.28)	1.01	0.98	1.28 (0.58, 2.79)	0.54	-	-
Current	89433 (92.96)	551 (90.62)	0.74	0.10	0.66 (0.37, 1.18)	0.16	-	-
Mean (SD) BMI	27.41 (4.56)	28.16 (5.11)	1.16	< 0.0001	1.25 (1.11, 1.41)	0.0002	1.09 (1.01, 1.18)	0.03
BMI categories								
Underweight	304 (0.32)	2 (0.33)	1.10	0.90	1.43 (0.20, 10.4)	0.73	-	-
Normal	31058 (32.28)	186 (30.59)	1	REF	1 (Reference)	-	-	-
Overweight	41705 (43.35)	242 (39.80)	0.97	0.75	1.01 (0.72, 1.42)	0.94	-	-
Obese	23139 (24.05)	178 (29.28)	1.28	0.02	1.44 (1.01, 2.05)	0.04	-	-
Mean (SD) Body fat-free mass	53.76 (11.49)	51.26 (11.04)	0.80	< 0.0001	1.42 (1.10, 1.84)	0.008	-	-
Mean (SD) Body fat mass	24.68 (9.21)	26.95 (10.05)	1.24	< 0.0001	1.27 (1.12, 1.44)	0.0002	-	-

For peer review only - http://bmjopen.bmj.com/site/about/guidelines.xhtml

Mean (SD) Body fat percent	31.18 (8.41)	34.01 (8.33)	1.39	< 0.0001	1.34 (1.11, 1.62)	0.002	-	-
Mean (SD) Systolic blood pressure	137.56 (18.10)	137.18 (18.02)	0.98	0.61	0.94 (0.81, 1.08)	0.39	-	-
Mean (SD) Diastolic blood pressure	82.20 (9.90)	81.48 (9.76)	0.93	0.08	0.90 (0.79, 1.04)	0.16	-	-
Mean (SD) FEV1	2.86 (0.78)	2.56 (0.75)	0.66	< 0.0001	0.70 (0.58, 0.86)	0.0005	0.79 (0.70, 0.89)	< 0.0001
Mean (SD) FVC	3.79 (0.99)	3.43 (0.94)	0.67	< 0.0001	0.72 (0.59, 0.90)	0.003	-	-
Mean (SD) FEV1/FVC	0.76 (0.06)	0.74 (0.07)	0.86	< 0.0001	0.84 (0.74, 0.95)	0.007	-	-
Walking pace								
Slow	50630 (52.63)	324 (53.29)	1.90	< 0.0001	1.91 (1.26, 2.90)	0.002	1.53 (1.18, 2.00)	0.001
Average	6144 (6.39)	75 (12.34)	1	REF	1 (Reference)	-	1 (Reference)	-
Brisk	39432 (40.99)	209 (34.38)	0.83	0.03	0.78 (0.57, 1.06)	0.11	0.96 (0.80, 1.15)	0.65
Mean (SD) grip strength	31.22 (10.96)	27.84 (10.44)	0.72	< 0.0001	0.85 (0.68, 1.07)	0.16	0.96 (0.76, 1.22)	0.76
Prevalent disease at baseline								
Longstanding illness	28729 (29.86)	259 (42.60)	1.74	< 0.0001	1.65 (1.25, 2.18)	0.0004	1.40 (1.04, 1.88)	0.03
Diabetes	4549 (4.73)	46 (7.57)	1.64	0.001	2.06 (1.29, 3.30)	0.002	1.59 (0.98, 2.59)	0.06
CVD	4842 (5.03)	46 (7.57)	1.54	0.005	1.64 (1.00, 2.70)	0.052	1.35 (0.81, 2.24)	0.25
Cancer	6525 (6.80)	59 (9.80)	1.48	0.004	1.72 (1.14, 2.60)	0.009	1.72 (1.14, 2.60)	0.01
Depression	18587 (19.32)	98 (16.12)	0.80	0.047	0.81 (0.56, 1.18)	0.27	0.86 (0.59, 1.25)	0.42
Asthma	11342 (11.79)	125 (20.56)	1.93	< 0.0001	1.44 (0.99, 2.09)	0.054	1.25 (0.85, 1.82)	0.25
COPD	238 (0.25)	3 (0.49)	1.99	0.23	-	-	-	-
Bronchitis	1074 (1.12)	17 (2.80)	2.52	0.0002	2.23 (0.98, 5.06)	0.055	1.77 (0.77, 4.07)	0.18
Pneumonia	1144 (1.19)	12 (1.97)	1.67	0.08	1.53 (0.57, 4.14)	0.40	1.47 (0.54, 3.99)	0.45
Other respiratory disease	507 (0.53)	5 (0.82)	1.56	0.32	0.84 (0.12, 6.04)	0.86	0.76 (0.10, 5.52)	0.78
Medication at baseline								
Statin	15265 (15.87)	132 (21.71)	1.47	< 0.0001	1.08 (0.75, 1.55)	0.69	0.91 (0.62, 1.32)	0.62
BP medication	15636 (16.25)	117 (19.24)	1.23	0.047	0.96 (0.66, 1.41)	0.85	0.82 (0.55, 1.21)	0.31
Steroid	352 (0.37)	3 (0.49)	1.35	0.61	1.20 (0.17, 8.63)	0.86	1.03 (0.14, 7.53)	0.97
Median (IQR) Biomarker at baseline								
Total cholesterol	5.62 (4.91-6.34)	5.55 (4.80-6.26)	0.94	0.10	0.94 (0.82, 1.08)	0.40	0.98 (0.85, 1.12)	0.76
HDL cholesterol	1.39 (1.17-1.67)	1.41 (1.17-1.70)	1.05	0.20	0.88 (0.76, 1.03)	0.11	0.98 (0.84, 1.16)	0.84

For peer review only

For peer review only - http://bmjopen.bmj.com/site/about/guidelines.xhtml

Cystatin C	0.88 (0.80-0.97)	0.90 (0.80-1.01)	1.17	< 0.0001	1.08 (0.94, 1.24)	0.29	0.96 (0.83, 1.12)	0.59
HbA1c	35.10 (32.60-37.60)	35.70 (33.38-38.60)	1.18	< 0.0001	1.19 (1.07, 1.33)	0.001	1.11 (0.99, 1.25)	0.07
CRP	1.27 (0.64-2.59)	1.50 (0.81-3.05)	1.14	< 0.0001	1.11 (1.00, 1.24)	0.04	1.03 (0.91, 1.16)	0.67
Rheumatoid factor	3.16 (3.16-3.16)	3.16 (3.16-3.16)	1.04	0.22	1.03 (0.91, 1.15)	0.68	1.02 (0.90, 1.15)	0.76
Red cell distribution width	13.30 (12.89-13.80)	13.37 (12.95-13.91)	1.10	0.01	1.12 (0.99, 1.27)	0.07	1.07 (0.95, 1.22)	0.27
White cell count	6.63 (5.64-7.80)	6.93 (5.90-8.10)	1.15	0.0002	1.15 (1.01, 1.32)	0.03	1.07 (0.93, 1.23)	0.32
Neutrophil count	4.01 (3.26-4.92)	4.19 (3.52-5.13)	1.12	0.003	1.11 (0.97, 1.27)	0.12	1.04 (0.91, 1.19)	0.57
Lymphocyte count	1.88 (1.52-2.29)	1.92 (1.60-2.35)	1.09	0.02	1.07 (0.94, 1.23)	0.28	1.02 (0.89, 1.17)	0.76
Monocyte count	0.45 (0.37-0.57)	0.46 (0.37-0.58)	1.06	0.13	1.11 (0.97, 1.28)	0.12	1.06 (0.92, 1.21)	0.43

Variables with associations denoted “-“ are excluded from the model due to non-significance in previous model and /or collinearity with another variable.

Model 1 adjusts for age, sex, ethnicity, deprivation

Model 2 adjusted for model 1 plus BMI, FEV1, and walking pace

For peer review only

Supplementary table 3. Linearity of continuous variables with COVID-19 and influenza.

	Covid-19			Pneumonia (after 2016)			Influenza (after 2016)		
	χ^2	df	$P_{nonlinear}$	χ^2	df	$P_{nonlinear}$	χ^2	df	$P_{nonlinear}$
Age	16.32	2.00	0.0003	2.36	1.99	0.30	1.99	2.00	0.37
Deprivation index	0.01	0.07	0.15	2.63	2.00	0.27	2.01	2.00	0.37
BMI	0.85	1.98	0.65	4.93	2.00	0.08	0.00	0.03	0.08
Body fat-free mass	5.44	1.98	0.06	5.45	1.92	0.06	1.32	2.00	0.52
Body fat mass	2.74	1.99	0.25	2.30	1.99	0.31	0.96	2.00	0.62
Body fat percent	0.66	1.97	0.71	1.00	2.00	0.61	0.00	0.02	0.09
Systolic blood pressure	1.65	2.00	0.44	0.84	1.99	0.65	2.24	2.00	0.33
Diastolic blood pressure	2.55	1.99	0.28	0.14	1.51	0.86	1.60	1.97	0.44
FEV1	1.04	1.98	0.59	8.50	1.92	0.01	4.07	1.94	0.12
FVC	0.00	0.03	0.08	8.95	1.95	0.01	1.42	1.99	0.49
FEV1/FVC	3.23	1.98	0.20	3.10	1.83	0.19	0.62	2.00	0.73
Grip strength	7.05	2.00	0.03	2.75	1.93	0.24	0.37	2.00	0.83
Biomarker at baseline									
Total cholesterol	7.40	1.95	0.02	0.94	1.99	0.62	2.50	1.99	0.28
HDL cholesterol	0.00	0.03	0.08	2.69	1.94	0.25	1.89	1.98	0.38
Cystatin C	3.02	1.99	0.22	1.31	1.99	0.52	1.05	1.99	0.59
HbA1c	6.23	2.00	0.04	3.71	2.00	0.16	4.93	2.00	0.08
CRP	11.86	1.99	0.003	7.84	1.99	0.02	5.08	2.00	0.08
Rheumatoid factor	2.22	2.00	0.33	4.24	2.00	0.12	1.70	1.99	0.43
Red cell distribution width	0.55	1.99	0.76	5.07	2.00	0.08	3.64	1.95	0.16
White cell count	5.25	2.00	0.07	1.43	2.00	0.49	0.00	0.03	0.08
Neutrophil count	3.51	1.97	0.17	1.33	2.00	0.51	2.44	1.94	0.28
Lymphocyte count	2.85	1.91	0.23	1.13	2.00	0.57	13.41	2.00	0.001
Monocyte count	0.01	0.04	0.11	1.14	1.76	0.50	9.36	1.91	0.008

For peer review only

Supplementary table 4. Association of risk factors for COVID-19 and pneumonia in UK Biobank with body fat percent replacing BMI in Model 2

	COVID-19		Pneumonia (after 2016)	
	RR (95% CI)	P	RR (95% CI)	P
Age	1.02 (0.95, 1.09)	0.63	1.11 (1.00, 1.23)	0.048
Male	2.72 (1.89, 3.93)	< 0.0001	1.05 (0.67, 1.63)	0.84
Ethnicity				
White	1 (Reference)		1 (Reference)	
Black	2.01 (1.13, 3.55)	0.02	1.22 (0.38, 3.94)	0.74
South Asian	1.60 (0.87, 2.93)	0.13	0.63 (0.19, 2.03)	0.44
Others	1.67 (0.98, 2.86)	0.06	0.51 (0.12, 2.12)	0.36
Deprivation index	1.18 (1.07, 1.29)	0.0009	1.04 (0.90, 1.20)	0.57
Current/former smoker	1.38 (1.12, 1.69)	0.002	0.89 (0.67, 1.18)	0.42
Body fat percent	1.42 (1.23, 1.65)	< 0.0001	1.20 (0.98, 1.46)	0.08
FEV1	0.84 (0.73, 0.97)	0.02	0.75 (0.61, 0.92)	0.005
Walking pace				
Slow	1.61 (1.18, 2.18)	0.002	1.66 (1.08, 2.55)	0.02
Average	1 (Reference)		1 (Reference)	
Brisk	0.95 (0.75, 1.19)	0.64	0.87 (0.63, 1.20)	0.39
grip strength	0.97 (0.83, 1.13)	0.71	0.98 (0.78, 1.24)	0.88
Prevalent disease at baseline				
Longstanding illness	1.16 (0.93, 1.44)	0.18	1.40 (1.04, 1.89)	0.02
Diabetes	1.30 (0.91, 1.85)	0.15	1.65 (1.02, 2.68)	0.04
CVD	1.22 (0.84, 1.76)	0.29	1.35 (0.81, 2.25)	0.25
Cancer	1.20 (0.83, 1.74)	0.33	1.72 (1.13, 2.60)	0.01
Depression	1.01 (0.77, 1.32)	0.94	0.86 (0.59, 1.25)	0.42
Asthma	1.05 (0.78, 1.41)	0.76	1.25 (0.86, 1.83)	0.25
COPD	1.77 (0.55, 5.67)	0.34	-	-
Bronchitis	1.24 (0.60, 2.55)	0.56	1.75 (0.76, 4.03)	0.19
Pneumonia	1.50 (0.74, 3.06)	0.26	1.46 (0.54, 3.98)	0.46
Other respiratory disease	1.20 (0.38, 3.79)	0.76	0.75 (0.10, 5.48)	0.78
Medication at baseline				
Statin	1.25 (0.98, 1.61)	0.08	0.92 (0.63, 1.34)	0.67
BP medication	1.35 (1.05, 1.72)	0.02	0.83 (0.56, 1.23)	0.35
Steroid	0.52 (0.07, 3.82)	0.52	1.03 (0.14, 7.51)	0.98
Biomarker				
Total cholesterol	0.92 (0.83, 1.01)	0.09	0.97 (0.85, 1.11)	0.67
HDL cholesterol	0.82 (0.72, 0.93)	0.002	0.97 (0.83, 1.14)	0.70
Cystatin C	1.14 (1.03, 1.26)	0.009	0.97 (0.84, 1.13)	0.72
HbA1c	1.15 (1.06, 1.24)	0.0003	1.13 (1.00, 1.26)	0.04
CRP	1.04 (0.95, 1.13)	0.38	1.04 (0.92, 1.17)	0.56
Rheumatoid factor	1.01 (0.92, 1.11)	0.85	1.02 (0.90, 1.15)	0.77
Red cell distribution width	1.04 (0.95, 1.14)	0.43	1.08 (0.95, 1.23)	0.23
White cell count	1.04 (0.94, 1.15)	0.43	1.08 (0.94, 1.23)	0.29
Neutrophil count	1.03 (0.93, 1.13)	0.62	1.04 (0.91, 1.20)	0.54
Lymphocyte count	1.03 (0.94, 1.14)	0.51	1.03 (0.90, 1.17)	0.71
Monocyte count	1.01 (0.92, 1.12)	0.77	1.06 (0.92, 1.22)	0.41

Data presented as risk ratios and their 95% CI. continuous exposures were standardised and presented as 1-standard deviation (SD) increment.

Adjusted for age, sex, ethnicity, deprivation, body fat %, FEV1, and walking pace

For peer review only - http://bmjopen.bmj.com/site/about/guidelines.xhtml

Supplementary Figure 1. CONSORT diagram.

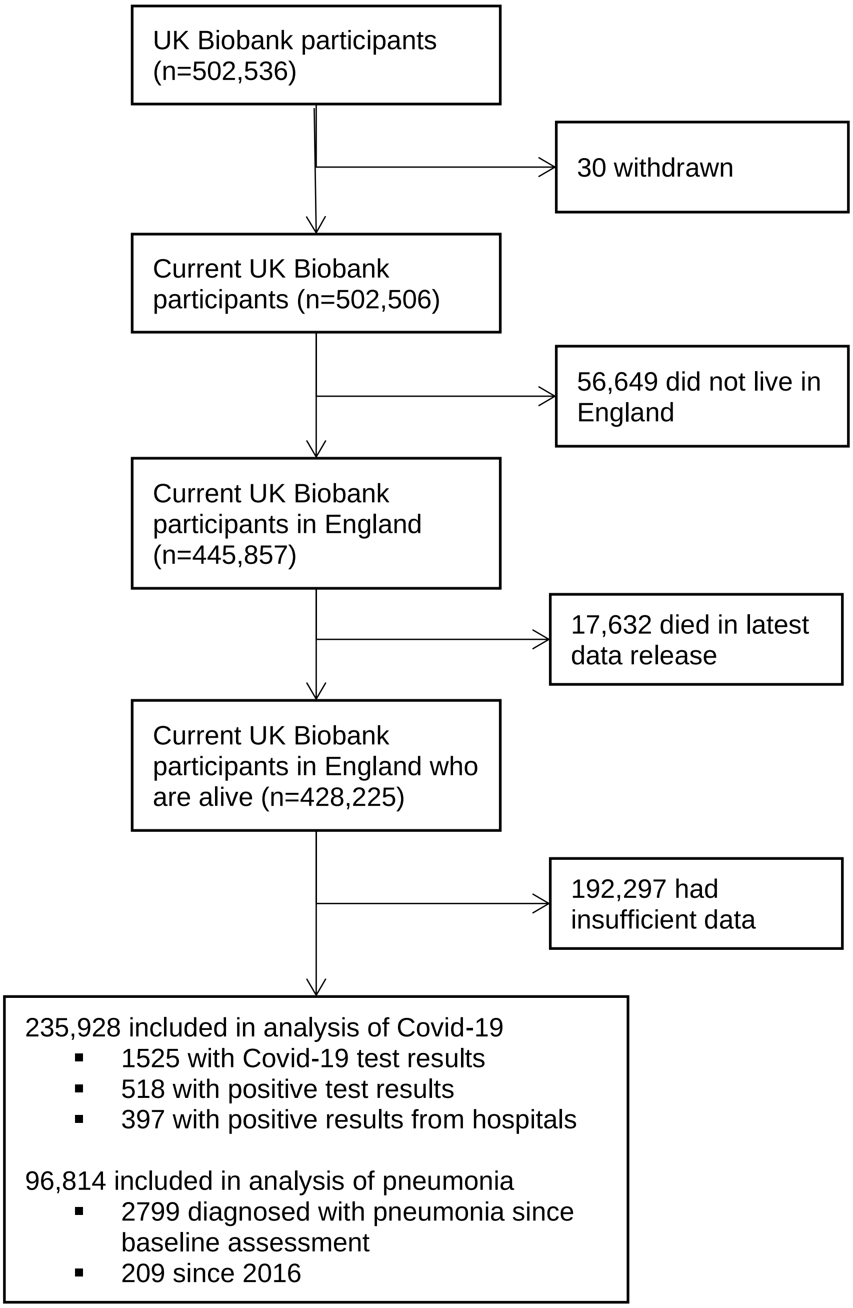

For peer review only - http://bmjopen.bmj.com/site/about/guidelines.xhtml

STROBE Statement—Checklist of items that should be included in reports of ***cohort studies***

	Item No	Recommendation	Page No
**Title and abstract**	1	(*a*) Indicate the study's design with a commonly used term in the title or the abstract	2
		(*b*) Provide in the abstract an informative and balanced summary of what was done and what was found	2
**Introduction**			
Background/rationale	2	Explain the scientific background and rationale for the investigation being reported	4
Objectives	3	State specific objectives, including any prespecified hypotheses	4
**Methods**			
Study design	4	Present key elements of study design early in the paper	5-6
Setting	5	Describe the setting, locations, and relevant dates, including periods of recruitment, exposure, follow-up, and data collection	5-6
Participants	6	(*a*) Give the eligibility criteria, and the sources and methods of selection of participants. Describe methods of follow-up<br>(*b*) For matched studies, give matching criteria and number of exposed and unexposed	5
Variables	7	Clearly define all outcomes, exposures, predictors, potential confounders, and effect modifiers. Give diagnostic criteria, if applicable	6-7
Data sources/ measurement	8*	For each variable of interest, give sources of data and details of methods of assessment (measurement). Describe comparability of assessment methods if there is more than one group	6-7
Bias	9	Describe any efforts to address potential sources of bias	6-7
Study size	10	Explain how the study size was arrived at	Figure s1
Quantitative variables	11	Explain how quantitative variables were handled in the analyses. If applicable, describe which groupings were chosen and why	7 Table 1
Statistical methods	12	(*a*) Describe all statistical methods, including those used to control for confounding<br>(*b*) Describe any methods used to examine subgroups and interactions<br>(*c*) Explain how missing data were addressed<br>(*d*) If applicable, explain how loss to follow-up was addressed<br>(*e*) Describe any sensitivity analyses	7-8
**Results**			
Participants	13*	(a) Report numbers of individuals at each stage of study—eg numbers potentially eligible, examined for eligibility, confirmed eligible, included in the study, completing follow-up, and analysed<br>(b) Give reasons for non-participation at each stage<br>(c) Consider use of a flow diagram	9 Fig s1
Descriptive data	14*	(a) Give characteristics of study participants (eg demographic, clinical, social) and information on exposures and potential confounders<br>(b) Indicate number of participants with missing data for each variable of interest<br>(c) Summarise follow-up time (eg, average and total amount)	Fig s1
Outcome data	15*	Report numbers of outcome events or summary measures over time	9

For peer review only - http://bmjopen.bmj.com/site/about/guidelines.xhtml

Main results	16	(*a*) Give unadjusted estimates and, if applicable, confounder-adjusted estimates and their precision (eg, 95% confidence interval). Make clear which confounders were adjusted for and why they were included<br>(*b*) Report category boundaries when continuous variables were categorized<br>(*c*) If relevant, consider translating estimates of relative risk into absolute risk for a meaningful time period	9-12
Other analyses	17	Report other analyses done—eg analyses of subgroups and interactions, and sensitivity analyses	Table s1-s4
**Discussion**			
Key results	18	Summarise key results with reference to study objectives	12
Limitations	19	Discuss limitations of the study, taking into account sources of potential bias or imprecision. Discuss both direction and magnitude of any potential bias	13-14
Interpretation	20	Give a cautious overall interpretation of results considering objectives, limitations, multiplicity of analyses, results from similar studies, and other relevant evidence	14
Generalisability	21	Discuss the generalisability (external validity) of the study results	13-14
**Other information**			
Funding	22	Give the source of funding and the role of the funders for the present study and, if applicable, for the original study on which the present article is based	15

*Give information separately for exposed and unexposed groups.

Note: An Explanation and Elaboration article discusses each checklist item and gives methodological background and published examples of transparent reporting. The STROBE checklist is best used in conjunction with this article (freely available on the Web sites of PLoS Medicine at http://www.plosmedicine.org/, Annals of Internal Medicine at http://www.annals.org/, and Epidemiology at http://www.epidem.com/). Information on the STROBE Initiative is available at http://www.strobe-statement.org.

For peer review only

For peer review only - http://bmjopen.bmj.com/site/about/guidelines.xhtml
